# Evolutionary diversification and characterization of the eubacterial gene family encoding DXR type II, an alternative isoprenoid biosynthetic enzyme

**DOI:** 10.1186/1471-2148-13-180

**Published:** 2013-09-03

**Authors:** Lorenzo Carretero-Paulet, Agnieszka Lipska, Jordi Pérez-Gil, Félix J Sangari, Victor A Albert, Manuel Rodríguez-Concepción

**Affiliations:** 1 Institute for Plant Molecular and Cell Biology - IBMCP (CSIC-UPV), Integrative Systems Biology Group, C/ Ingeniero Fausto Elio s/n., Valencia 46022, Spain; 2 Department of Biological Sciences, SUNY-University at Buffalo, North Campus. 109 Cooke Hall, Buffalo, NY 14260, USA; 3 Centre for Research in Agricultural Genomics (CRAG), CSIC-IRTA-UAB-UB, Campus UAB, Bellaterra, Barcelona 08193, Spain; 4 Department of Molecular Biology, Universidad de Cantabria and Instituto de Biomedicina y Biotecnología de Cantabria (IBBTEC), UC-CSIC-SODERCAN, Avda. de los Castros s/n, Santander E-39005, Cantabria, Spain

**Keywords:** DXR-II, Isoprenoid metabolism, Horizontal gene transfer, Gene loss, Functional divergence

## Abstract

**Background:**

Isoprenoids constitute a vast family of natural compounds performing diverse and essential functions in all domains of life. In most eubacteria, isoprenoids are synthesized through the methylerythritol 4-phosphate (MEP) pathway. The production of MEP is usually catalyzed by deoxyxylulose 5-phosphate reductoisomerase (DXR-I) but a few organisms use an alternative DXR-like enzyme (DXR-II).

**Results:**

Searches through 1498 bacterial complete proteomes detected 130 sequences with similarity to DXR-II. Phylogenetic analysis identified three well-resolved clades: the DXR-II family (clustering 53 sequences including eleven experimentally verified as functional enzymes able to produce MEP), and two previously uncharacterized NAD(P)-dependent oxidoreductase families (designated DLO1 and DLO2 for DXR-II-like oxidoreductases 1 and 2). Our analyses identified amino acid changes critical for the acquisition of DXR-II biochemical function through type-I functional divergence, two of them mapping onto key residues for DXR-II activity. DXR-II showed a markedly discontinuous distribution, which was verified at several levels: taxonomic (being predominantly found in Alphaproteobacteria and Firmicutes), metabolic (being mostly found in bacteria with complete functional MEP pathways with or without DXR-I), and phenotypic (as no biological/phenotypic property was found to be preferentially distributed among DXR-II-containing strains, apart from pathogenicity in animals). By performing a thorough comparative sequence analysis of GC content, 3:1 dinucleotide frequencies, codon usage and codon adaptation indexes (CAI) between DXR-II sequences and their corresponding genomes, we examined the role of horizontal gene transfer (HGT), as opposed to an scenario of massive gene loss, in the evolutionary origin and diversification of the DXR-II subfamily in bacteria.

**Conclusions:**

Our analyses support a single origin of the DXR-II family through functional divergence, in which constitutes an exceptional model of acquisition and maintenance of redundant gene functions between non-homologous genes as a result of convergent evolution. Subsequently, although old episodic events of HGT could not be excluded, the results supported a prevalent role of gene loss in explaining the distribution of DXR-II in specific pathogenic eubacteria. Our results highlight the importance of the functional characterization of evolutionary shortcuts in isoprenoid biosynthesis for screening specific antibacterial drugs and for regulating the production of isoprenoids of human interest.

## Background

**Figure 1:**
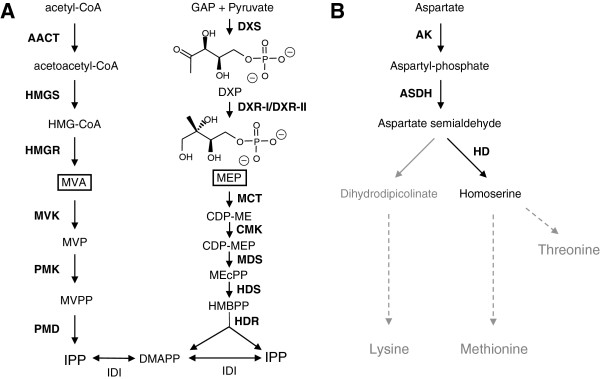
**Isoprenoid and amino acid biosynthetic pathways. A)** Pathways for the biosynthesis of isoprenoid precursors. On the left, MVA pathway is represented. Enzymes are indicated in bold: AACT, acetoacetyl-CoA thiolase; HMGS, HMG-CoA synthase; HMGR, HMG-CoA reductase; MVK, mevalonate kinase; PMVK, 5-phosphomevalonate kinase; DPMD, 5-diphosphomevalonate decarboxylase; IDI, IPP/DMAPP isomerase. On the right, MEP pathway steps are described: GAP, D-glyceraldehyde 3-phosphate; DXP, 1-deoxy-D-xylulose 5-phosphate; MEP, 2-C-methyl-D-erythritol 4-phosphate; CDP-ME,4-diphosphocytidyl-2-C-methyl-D-erythritol; MEcPP, 2-C-methyl-D-erythritol 2,4-cyclodiphosphate; HMBPP, 4-hydroxy-3-methylbut-2-enyl diphosphate; IPP, isopentenyl diphosphate; DMAPP, dimethylallyl diphosphate. Enzymes are indicated in bold: DXS, DXP synthase; DXR, DXP reductoisomerase; MCT, MEP cytidylyltransferase; CMK, CDPME kinase; MDS, ME-cPP synthase; HDS, HMBPP synthase; HDR, HMBPP reductase; IDI, IPP isomerase. **B)** Amino acid biosynthesis. The common pathway (CP) is highlighted in black and enzymes indicated in bold: AK, aspartokinase; ASDH, aspartate semialdehyde dehydrogenase; HD, homoserine dehydrogenase. Solid arrows indicate single catalytic steps and dashed arrows mark multiple steps.

Isoprenoids constitute the largest family of natural compounds both at a structural and functional level [1-3]. They are found in all the three domains of life (bacteria, archaea, and eukaryotes). Despite their diversity in structures and functions, all isoprenoids derive from the common five-carbon precursors isopentenyl diphosphate (IPP) and its isomer dimethylallyl diphosphate (DMAPP). IPP can be synthesized through two independent metabolic pathways, the mevalonate (MVA) pathway, or the more recently elucidated methylerythritol 4-phosphate (MEP) pathway [4] (Figure 1). In most eubacteria, isoprenoids are synthesized through the MEP pathway, while a few species use the MVA pathway, both pathways, or none, the latter obtaining their isoprenoids from host cells [5-8]. Previous analysis suggested that eukaryotes have inherited MEP and MVA pathways genes from eubacteria and archaebacteria, respectively, as reflected by their phylogenetic distribution [5]. In plants, plastidial IPP and DMAPP are synthesized through the MEP pathway, whereas cytosolic and mitochondrial isoprenoids are synthesized through the MVA pathway [4,9]. Non-photosynthetic simpler plastid-bearing organisms, such as the apicomplexan protists, solely use the MEP pathway [10]. In contrast, in yeast and animals, all isoprenoids are synthesized through the MVA pathway [11]. The lack of MEP pathway enzymes in non-plastid bearing eukaryotes suggests that these genes were acquired through gene transfer to the nucleus from the eubacterial endosymbiotic ancestors that gave rise to plastids [5,12].

Isoprenoids are essential in all eubacteria in which they have been studied, playing key roles in several core cellular functions e.g. ubiquinones and menaquinones, which act as electron carriers of the aerobic and anaerobic respiratory chains respectively, and dolichols, which are required for cell wall peptidoglycan synthesis [13]. Because of the essential role of the MEP pathway in most eubacteria and its absence from animals, it has been proposed as a promising new target for the development of novel antibiotics [14,15]. Besides that, many isoprenoids also have substantial industrial, pharmacological, and nutritional interest [16]. Therefore, understanding the biochemical and genetic plasticity of isoprenoid biosynthesis in bacteria is crucial to attempt its pharmacological block or to be used in biofactories for the production of isoprenoids of human interest.

The occurrence of alternative enzymes for isoprenoid biosynthesis in specific bacterial lineages has been previously reported [17]. The enzyme 3-hydroxy-3-methyl-glutaryl-CoA reductase (HMGR), which catalyzes the rate-limiting step of the MVA pathway, is structurally distant from its archaebacterial and eukaryotic homologs in most eubacteria [8,18,19]. Similarly, two different classes of isopentenyl diphosphate isomerase (IDI), the enzyme catalyzing the isomerization of IPP to produce DMAPP, have been identified in bacteria: type I IDI (similar to its animal, fungi and plant counterparts) and type II IDI, acquired from archaebacteria and apparently unrelated to the latter [20-22]. Although IDI activity is only essential in organisms dependent on the MVA pathway for IPP and isoprenoid biosynthesis, both types of IDI have been identified in bacterial strains dependent on the MEP pathway [7].

We recently reported the occurrence of a group of bacteria harbouring the entire set of enzymes of the MEP pathway with the exception of 1-deoxy-d-xylulose 5-phosphate (DXP) reductoisomerase (DXR), the enzyme catalyzing the NADPH-dependent production of MEP from DXP in the first committed step of the pathway. In these species, a novel family of previously uncharacterized oxidoreductases related to homoserine dehydrogenases (HD) involved in the common pathway (CP) of amino acid biosynthesis (Figure 1), was found to perform the DXR biochemical reaction [23]. This alternative enzyme, referred to as DXR-like (DRL) or DXR type II (DXR-II) to distinguish it from the canonical DXR (renamed DXR-I), displayed a markedly discontinuous distribution. DXR-II was found forming single or multigene families in bacterial strains from diverse taxonomic groups, independent of the presence or absence of a DXR-I sequence in their genome [23].

Different evolutionary scenarios might explain DXR-II emergence and evolutionary diversification. In this study we examined how the DXR-II family emerged through functional divergence from related oxidoreductase families and identified amino acid changes critical for the acquisition of its specific biochemical function. Furthermore, we assess the contrasting roles of horizontal gene transfer (HGT) and massive gene loss, major forces in microbial genome evolution known to affect other genes involved in IPP and isoprenoid biosynthesis [24], in the discontinuous distribution of DXR-II across eubacteria.

## Results

### DXR-IIs cluster into a single clade closely related to two uncharacterized oxidoreductase families

**Figure 2:**
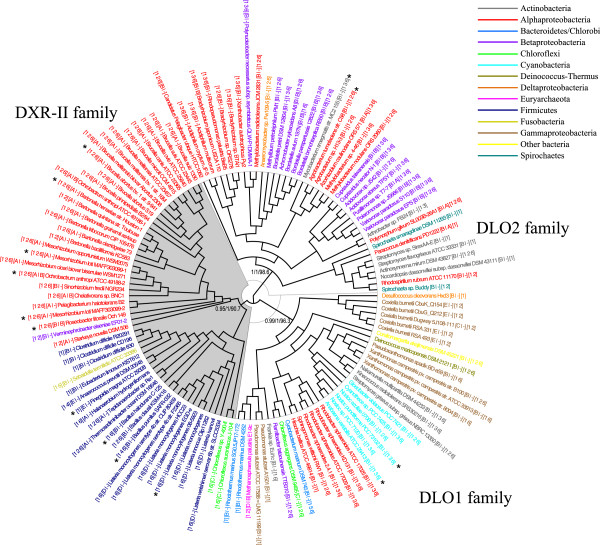
**Phylogeny of DXR-II and DLO related sequences.** ML cladogram depicting the evolutionary relationships among 53 DXR-II and 77 related protein sequences. Three clades defining main families are indicated. Statistical support on relevant clades is indicated by values next to nodes (ML aLRT support values/BA posterior probabilities/NJ bootstrap values). Sequence names are colored according to taxonomical groups (see legend). Sequence names include the bacterial strain name, followed by two pairs of square brackets: the first pair encloses the classification of the given bacterial strain according to the distribution of enzymes of the i) MEP and MVA pathways, left side of the vertical bar (i.e. classes A, +MEP pathway enzymes –DXR; B, +MEP pathway enzymes + DXR; C, -MEP + MVA pathway enzymes -DXR; D, +MEP + MVA pathway enzymes + DXR; E, -MEP -MVA pathway enzymes -DXR) and ii) CP pathway, right side of the vertical bar (i.e. A, complete CP pathway; B, incomplete CP pathway –AK_HD). The second pair of brackets represents the INTERPRO protein functional domains found i.e. 1, NAD(P)-binding domain (IPR016040); 2, Aspartate/homoserine dehydrogenase, NAD-binding (IPR005106); 3, Oxidoreductase, N-terminal (IPR000683); 4, Dihydrodipicolinate reductase, N-terminal (IPR000846); 5, Quinate/shikimate 5-dehydrogenase/glutamyl-tRNA reductase (IPR006151); 6, SAF domain (IPR013974). Asterisks indicate sequences for which DXR-II activity was previously tested through complementation assays [23] and Additional file 5.

The complete proteomes of 1489 eubacterial strains were screened for the occurrence of DXR-II sequences using the protein sequence from *Brucella melitensis biovar abortus 2308* DXR-II (formerly *Brucella abortus* 2308, gene id: 83269188) as a query [23]. To reduce false positives caused by hits corresponding to distantly related sequences, we applied a best reciprocal hit criterion i.e. orthology was assumed only if two genes in each different genome are each other’s best hit [25]. Indeed, eight sequences were not confirmed as reciprocal best hits, including two identified in a previous survey conducted following a unidirectional BLAST search approach [23], and these were consequently discarded from further analyses. 128 sequence hits were identified in as many bacterial strains (Table 1), belonging to a wide variety of the main bacterial taxonomic groups (Figure 2). Among these, two bacterial strains (*Mesorhizobium loti MAFF303099* and *Ochrobactrum anthropi ATCC 49188*) had been previously shown to code for additional functional DXR-II paralogs [23] that were not identified by our analysis, specifically designed to identify co-orthologs in genome wide scans, but were added to the final dataset (Table 1).

**Table 1:** List of DXR-II and DLO related sequences examined in this study

	***Bacterial strain***	**UID**	**GenBank and RefSeq**		***Bacterial strain***	**UID**	**GenBank and RefSeq**
**DXR-II**	*Anaerococcus prevotii DSM 20548*	59219	gi|257066990|ref|YP_003153246.1	**DLO1**	*Frankia sp. EuI1c*	42615	gi|312199021|ref|YP_004019082.1
	*Bacillus clausii KSM-K16*	58237	gi|56965002|ref|YP_176733.1		*Gloeobacter violaceus PCC 7421*	58011	gi|37521773|ref|NP_925150.1
	*Bacillus halodurans C-125*	57791	gi|15613337|ref|NP_241640.1		*Hirschia baltica ATCC 49814*	59365	gi|254294497|ref|YP_003060520.1
	*Bacillus pumilus SAFR-032*	59017	gi|157692210|ref|YP_001486672.1		*Kineococcus radiotolerans SRS30216*	58067	gi|152964541|ref|YP_001360325.1
	*Bartonella bacilliformis KC583*	58533	gi|121601844|ref|YP_989368.1		*Methanosphaerula palustris E1-9c*	59193	gi|219852978|ref|YP_002467410.1
	*Bartonella clarridgeiae 73*	62131	gi|319898668|ref|YP_004158761.1		*Nakamurella multipartita DSM 44233*	59221	gi|258653356|ref|YP_003202512.1
	*Bartonella grahamii as4aup*	59405	gi|240851045|ref|YP_002972445.1		*Nostoc azollae 0708*	49725	gi|298491811|ref|YP_003721988.1
	*Bartonella henselae str. Houston-1*	57745	gi|49475991|ref|YP_034032.1		*Nostoc punctiforme PCC 73102*	57767	gi|186681545|ref|YP_001864741.1
	*Bartonella quintana str. Toulouse*	57635	gi|49474558|ref|YP_032600.1		*Nostoc sp. PCC 7120*	57803	gi|17230323|ref|NP_486871.1
	*Bartonella tribocorum CIP 105476*	59129	gi|163868831|ref|YP_001610057.1		*Pseudomonas stutzeri A1501*	58641	gi|146282531|ref|YP_001172684.1
	*Brucella abortus bv. 1 str. 9-941*	58019	gi|62317206|ref|YP_223059.1		*Pseudomonas stutzeri ATCC 17588 = LMG 11199*	68749	gi|339494143|ref|YP_004714436.1
	*Brucella abortus S19*	58873	gi|189022468|ref|YP_001932209.1		*Pseudoxanthomonas spadix BD-a59*	75113	gi|357416048|ref|YP_004929068.1
	*Brucella canis ATCC 23365*	59009	gi|161621022|ref|YP_001594908.1		*Ramlibacter tataouinensis TTB310*	68279	gi|337280130|ref|YP_004619602.1
	*Brucella melitensis ATCC 23457*	59241	gi|225686729|ref|YP_002734701.1		*Rhodobacter sphaeroides 2.4.1*	57653	gi|77463590|ref|YP_353094.1
	*Brucella melitensis biovar Abortus 2308*	62937	gi|83269188|ref|YP_418479.1		*Rhodobacter sphaeroides ATCC 17025*	58451	gi|146278215|ref|YP_001168374.1
	*Brucella melitensis bv. 1 str. 16 M*	57735	gi|17988671|ref|NP_541304.1		*Rhodobacter sphaeroides ATCC 17029*	58449	gi|126462422|ref|YP_001043536.1
	*Brucella microti CCM 4915*	59319	gi|256015731|ref|YP_003105740.1		*Rhodobacter sphaeroides KD131*	59277	gi|221639432|ref|YP_002525694.1
	*Brucella ovis ATCC 25840*	58113	gi|148558391|ref|YP_001257886.1		*Rhodothermus marinus DSM 4252*	41729	gi|268316714|ref|YP_003290433.1
	*Brucella pinnipedialis B2/94*	71131	gi|340792737|ref|YP_004758201.1		*Rhodothermus marinus SG0.5JP17-172*	72767	gi|345303494|ref|YP_004825396.1
	*Brucella suis 1330*	57927	gi|23500696|ref|NP_700136.1		*Sphingomonas wittichii RW1*	58691	gi|148557435|ref|YP_001265017.1
	*Brucella suis ATCC 23445*	59015	gi|163845083|ref|YP_001622738.1		*Streptomyces griseus subsp. griseus NBRC 13350*	58983	gi|182439707|ref|YP_001827426.1
	*Chelativorans sp. BNC1*	58069	gi|110636013|ref|YP_676221.1		*Xanthomonas campestris pv. campestris str. 8004*	57595	gi|77761197|ref|YP_243248.2
	*Chloroflexus aurantiacus J-10-fl*	57657	gi|163846900|ref|YP_001634944.1		*Xanthomonas campestris pv. campestris str. ATCC 33913*	57887	gi|77747863|ref|NP_637377.2
	*Chloroflexus sp. Y-400-fl*	59085	gi|222524722|ref|YP_002569193.1		*Xanthomonas campestris pv. campestris str. B100*	61643	gi|188991706|ref|YP_001903716.1
	*Clostridium difficile 630*	57679	gi|126700028|ref|YP_001088925.1	**DLO2**	*Achromobacter xylosoxidans A8*	59899	gi|311109080|ref|YP_003981933.1
	*Clostridium difficile CD196*	41017	gi|260683992|ref|YP_003215277.1		*Acidiphilium cryptum JF-5*	58447	gi|148260557|ref|YP_001234684.1
	*Clostridium difficile R20291*	40921	gi|260687652|ref|YP_003218786.1		*Acidiphilium multivorum*	63345	gi|326403752|ref|YP_004283834.1
	*Eubacterium limosum KIST612*	59777	gi|310828050|ref|YP_003960407.1		*Acidovorax ebreus TPSY*	59233	gi|222110742|ref|YP_002553006.1
	*Finegoldia magna ATCC 29328*	58867	gi|169824217|ref|YP_001691828.1		*Acidovorax sp. JS42*	58427	gi|121594656|ref|YP_986552.1
	*Halanaerobium hydrogeniformans*	60191	gi|312144614|ref|YP_003996060.1		*Actinosynnema mirum DSM 43827*	58951	gi|256377798|ref|YP_003101458.1
	*Listeria innocua Clip11262*	61567	gi|16799625|ref|NP_469893.1		*Agrobacterium sp. H13-3*	63403	gi|332715931|ref|YP_004443397.1
	*Listeria ivanovii*	73473	gi|347547952|ref|YP_004854280.1		*Agrobacterium tumefaciens str. C58*	57865	gi|15891768|ref|NP_357440.1
	*Listeria monocytogenes*	43671	gi|284800826|ref|YP_003412691.1		*Anaeromyxobacter sp. Fw109-5*	58755	gi|153005951|ref|YP_001380276.1
	*Listeria monocytogenes 08-5923*	43727	gi|284994012|ref|YP_003415780.1		*Arthrobacter sp. FB24*	58141	gi|116672147|ref|YP_833080.1
	*Listeria monocytogenes EGD-e*	61583	gi|16802589|ref|NP_464074.1		*Azorhizobium caulinodans ORS 571*	58905	gi|158423518|ref|YP_001524810.1
	*Listeria monocytogenes HCC23*	59203	gi|217965360|ref|YP_002351038.1		*Bordetella avium 197 N*	61563	gi|187476836|ref|YP_784860.1
	*Listeria monocytogenes serotype 4b str. CLIP 80459*	59317	gi|226223175|ref|YP_002757282.1		*Bordetella bronchiseptica RB50*	57613	gi|33599421|ref|NP_886981.1
	*Listeria monocytogenes serotype 4b str. F2365*	57689	gi|46906791|ref|YP_013180.1		*Bordetella parapertussis 12822*	57615	gi|33595139|ref|NP_882782.1
	*Listeria welshimeri serovar 6b str. SLCC5334*	61605	gi|116871936|ref|YP_848717.1		*Bordetella petrii DSM 12804*	61631	gi|163858833|ref|YP_001633131.1
	*Mesorhizobium ciceri biovar biserrulae WSM1271*	62101	gi|319781195|ref|YP_004140671.1		*Bradyrhizobium japonicum USDA 110*	57599	gi|27382926|ref|NP_774455.1
	*Mesorhizobium loti MAFF303099 (1)*	57601	gi|13473132|ref|NP_104699.1		*Bradyrhizobium sp. BTAi1*	58505	gi|148252763|ref|YP_001237348.1
	*Mesorhizobium loti MAFF303099 (2)*	57601	gi|13475431|ref|NP_106995.1		*Bradyrhizobium sp. ORS278*	58941	gi|146343223|ref|YP_001208271.1
	*Mesorhizobium opportunistum WSM2075*	40861	gi|337266026|ref|YP_004610081.1		*Candidatus Pelagibacter ubique HTCC1062*	58401	gi|71083552|ref|YP_266271.1
	*Ochrobactrum anthropi ATCC 49188 (1)*	58921	gi|153008718|ref|YP_001369933.1		*Cupriavidus necator N-1*	68689	gi|339328796|ref|YP_004688488.1
	*Ochrobactrum anthropi ATCC 49188 (2)*	58921	gi|153011435|ref|YP_001372649.1		*Cupriavidus taiwanensis*	61615	gi|194292943|ref|YP_002008850.1
	*Pelagibacterium halotolerans B2*	74393	gi|357386128|ref|YP_004900852.1		*Methylibium petroleiphilum PM1*	58085	gi|124268433|ref|YP_001022437.1
	*Roseobacter litoralis Och 149*	54719	gi|339504759|ref|YP_004692179.1		*Methylobacterium nodulans ORS 2060*	59023	gi|220926646|ref|YP_002501948.1
	*Sebaldella termitidis ATCC 33386*	41865	gi|269122365|ref|YP_003310542.1		*Methylobacterium radiotolerans JCM 2831*	58845	gi|170751253|ref|YP_001757513.1
	*Sinorhizobium fredii NGR234*	59081	gi|227820170|ref|YP_002824141.1		*Methylobacterium sp. 4-46*	58843	gi|170738904|ref|YP_001767559.1
	*Starkeya novella DSM 506*	48815	gi|298294348|ref|YP_003696287.1		*Mycobacterium smegmatis str. MC2 155*	57701	gi|118472915|ref|YP_885297.1
	*Tepidanaerobacter sp. Re1*	66873	gi|332798945|ref|YP_004460444.1		*Nocardiopsis dassonvillei subsp. dassonvillei DSM 43111*	49483	gi|297561288|ref|YP_003680262.1
	*Thermosediminibacter oceani DSM 16646*	51421	gi|302389988|ref|YP_003825809.1		*Paracoccus denitrificans PD1222*	58187	gi|119386102|ref|YP_917157.1
	*Verminephrobacter eiseniae EF01-2*	58675	gi|121609190|ref|YP_996997.1		*Polaromonas sp. JS666*	58207	gi|91787595|ref|YP_548547.1
**DLO1**	*Anabaena variabilis ATCC 29413*	58043	gi|75907337|ref|YP_321633.1		*Polymorphum gilvum SL003B-26A1*	65447	gi|328544682|ref|YP_004304791.1
	*Chloroflexus aggregans DSM 9485*	58621	gi|219849032|ref|YP_002463465.1		*Polynucleobacter necessarius subsp. asymbioticus QLW-P1DMWA-1*	58611	gi|145589731|ref|YP_001156328.1
	*Coraliomargarita akajimensis DSM 45221*	47079	gi|294053940|ref|YP_003547598.1		*Pusillimonas sp. T7-7*	66391	gi|332284324|ref|YP_004416235.1
	*Coxiella burnetii CbuG_Q212*	58893	gi|212211864|ref|YP_002302800.1		*Rhodopseudomonas palustris BisB5*	58441	gi|91978550|ref|YP_571209.1
	*Coxiella burnetii CbuK_Q154*	58895	gi|212217809|ref|YP_002304596.1		*Rhodospirillum rubrum ATCC 11170*	57655	gi|83594471|ref|YP_428223.1
	*Coxiella burnetii Dugway 5 J108-111*	58629	gi|154707185|ref|YP_001423500.1		*Spirochaeta smaragdinae DSM 11293*	51369	gi|302337774|ref|YP_003802980.1
	*Coxiella burnetii RSA 331*	58637	gi|161830312|ref|YP_001597660.1		*Spirochaeta sp. Buddy*	63633	gi|325972507|ref|YP_004248698.1
	*Coxiella burnetii RSA 493*	57631	gi|29655123|ref|NP_820815.1		*Streptomyces flavogriseus ATCC 33331*	40839	gi|357414986|ref|YP_004926722.1
	*Cyanothece sp. PCC 7425*	59435	gi|220910534|ref|YP_002485845.1		*Streptomyces sp. SirexAAcpoE*	72627	gi|345003166|ref|YP_004806020.1
	*Cyclobacterium marinum DSM 745*	71485	gi|343084038|ref|YP_004773333.1		*Variovorax paradoxus EPS*	62107	gi|319794630|ref|YP_004156270.1
	*Deinococcus maricopensis DSM 21211*	62225	gi|320332781|ref|YP_004169492.1		*Variovorax paradoxus S110*	59437	gi|239816446|ref|YP_002945356.1
	*Desulfococcus oleovorans Hxd3*	58777	gi|158521221|ref|YP_001529091.1		*Xanthobacter autotrophicus Py2*	58453	gi|154244830|ref|YP_001415788.1

*UID* (taxonomy) Unique IDentifier.

Using the amino acid sequence alignment of the resulting full dataset of 130 hits (Additional file 1), a maximum likelihood (ML) phylogenetic analysis was performed (Figure 2 and Additional file 2). Alternative methods of phylogenetic inference (Bayesian -Additional file 3- and neighbor joining -Additional file 4) were also implemented, resulting in trees with almost identical topologies (unpublished data). Three main clades were consistently retrieved with high support values (Figure 2). A clade grouping 53 sequences, including 11 encoding for functional DXR-II as shown in complementation assays in [23] and Additional file 5, was designated as the DXR-II family and likely corresponds to actual DXR-II sequences (Figure 2). The remaining 77 sequences cluster into two additional clades and might not be true functional DXR-II sequences (Figure 2). As such, these were tentatively designated DLO1 and DLO2, for DXR-II-Like 1 and 2 Oxidoreductases. Indeed, four representative sequences belonging to the DLO1 and 2 families had also been previously tested for DXR-II activity, failing to complement the DXR defective mutant (Figure 2) [23].

DXR-II and DLO sequences showed similarity to NAD(P)-dependent oxidoreductases, and particularly to HD enzymes, at a sequence [23] and structural level [26]. Correspondingly, searches for INTERPRO functional domains identified the NAD-binding domain with a core Rossmann-type fold at the N-terminal region of every single protein sequence (domain 1; Figure 2). Up to five additional domains could also be found in DXR-II and DLO proteins. To examine whether these protein domains were differentially distributed across the DXR-II, DLO1, and DLO2 families, we mapped the architecture of protein domains onto the corresponding tree (Figure 2). Most sequences from the DXR-II family shared NAD-binding (domain 1) and SAF (domain 6) domains, while a significant fraction also included N-terminal NAD/NADP-binding domains of aspartate/homoserine dehydrogenase (domain 2). However, no common domain architecture was shared among proteins within families DLO1 and DLO2.

### The DXR-II family emerged through functional divergence

Phylogenetic analysis revealed the shared ancestry of all functional DXR-II, supporting their common evolutionary origin, and suggested the functional divergence of this family from related oxidoreductases through the acquisition of DXR-II specific biochemical activity. To examine the role of specific amino acid substitutions in functional specialization of DXR-II protein sequences, two different statistical approaches under a ML framework were followed. The first one permits the detection of amino acid sites subjected to different evolutionary rates between families under examination, i.e., highly conserved in a family but variable in the other (type-I functional divergence) [27]. The second approach relies on site-specific shifts of amino acid physiochemical properties in positions otherwise highly conserved in each family (type-II functional divergence) [28].

Given the ML tree topology (Figure 2), the ML estimates of the theta (θ) coefficients for type-I functional divergence between the DXR-II family and families DLO1 and DLO2 were statistically significant in both cases (Table 2). This implies that structural and/or functional selective constraints at some sites have shifted significantly after the divergence of DXR-II from both DLO families. In contrast, the corresponding tests did not support type-II functional divergence (Table 2). Moreover, 28 and 34 specific amino acid residues, including 8 and 11 with high posterior probabilities, were predicted as responsible for type-I functional divergence of DXR-II from DLO families 1 and 2, respectively (Table 2). Interestingly, seven sites detected as key for functional divergence were shared in analyses between the DXR-II family and both the DLO1 and DLO2 families.

**Table 2:** Analysis of functional divergence

**Functional divergence**	**Families**	**Coefficient θ ± SE**	**Critical amino acid sites (Q**_**k**_ > 0.7; *, Q_**k**_ **> 0.95)**
**Type I**	**DXR-II *vs *DLO1**	**θ**_1_ = 0.277 ± 0.045 (LRT = 83.233; p = 7.292E-20 )	**35**, 46, 118, 121, 146, 161*, 176, 198*, 205*, 218, 229, 234, 237, 247*, 265, 282*, **291**, 297, **310**, 340, 342, 351*, 353*, 376, 404*, **410**, 422, 424, 429
	**DXR-II *vs *DLO2**	**θ**_1_ = 0.253 ± 0.043 (LRT = 114.991; p = 7.907E-27 )	**35**, 47, 64, 122*, 128, 133, 197*, 202, **205**, 210, 239, 248, 250*, 253*, 258*, 260, **282**, **291**, 296, 305, 310*, 311*, 314, 320, 324*, 330*, 346, 351*, 359, 383*, 410*, 413, 428*, 432
**Type II**	**DXR-II *vs *DLO1**	**θ**_2_ = −0.998 ± 0.487	
	**DXR-II *vs *DLO2**	**θ**_2_ = −1.115 ± 0.575	

p = posterior probability values; *SE* Standard Error. LRT and resulting p-values are shown in parentheses. Critical amino acid sites detected as related to functional divergence with Q_k_ > 70% (*, Q_k_ > 95%) are listed. Seven sites predicted as related to functional divergence of DXR-II from both families DLO1 and DLO2 are indicated in bold. Numbering refers to *Brucella melitensis biovar abortus 2308* DXR-II protein sequence.

These sites were mapped onto the corresponding amino acid sequence alignment (Additional file 1 and Additional file 6: Table S1). At many of these sites, amino acid residues are highly conserved in DXR-II sequences, but are variable in the DLO1 (e.g. positions 161 and 429 in *B. melitensis biovar abortus 2308* DXR-II), the DLO2 (e.g. positions 210, 248 and 324), or both the DLO1 and the DLO2 (e.g. positions 35, 64, 118, 121, 122, 133, 197, 229, 250, 291, 320, 330, 346, 351, 353, 413, 428, 429, 432) families, likely reflecting a change in their functional roles. Some apparently represented minor changes, as they involved amino acids with similar physicochemical features (e.g. positions 291 or 428). Some others involved radical amino acid changes, such as position 121, occupied by the highly conserved Gly in DXR-II proteins, but also by the unrelated Ala and Ser amino acids in DLO1 and DLO2 proteins. Another example is position 229, filled by the absolutely conserved polar amino acid Thr in DXR-II proteins, but replaced by the highly hydrophobic Leu, Ile and Val amino acids in DLO1 or the physicochemically unrelated Pro, Ser and Ala residues in DLO2. Likewise, position 250, with a basic polar His found in all but four DXR-II proteins was replaced by different hydrophobic amino acids, and finally position 351, with a conserved Val in most DXR-II proteins was substituted by different physicochemically unrelated amino acids in DLO1 and DLO2 proteins.

**Figure 3:**
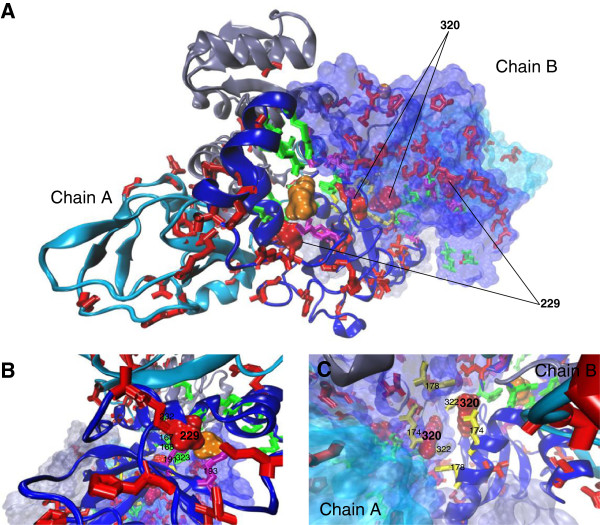
**3D architecture of DXR-II showing relevant and functional divergence residues. A**, view of the DXR-II dimer. Chain A is represented as cartoon backbone highlighting secondary structures and chain B as its molecular surface equivalent. **B**, Close-up view of the active site and of residues participating in substrate/fosmidomycin binding, including position 229, also predicted as related to functional divergence. **C**, Close-up view of the cavity at the dimer interface highlighting conserved residues involved in interactions between the two subunits of the DXR-II dimer, including position 320, also predicted as related to functional divergence. The N-terminal, central and C-terminal domains are shown in grey, blue and cyan, respectively. Residues predicted as involved in functional divergence of DXR-II are shown in red. Residues identified as involved in dimerization, fosmidomycin/substrate binding and the active site are shown in yellow, violet and green, respectively. The competitive inhibitor fosmidomycin is colored in orange. Molecular graphics were produced with VMD 1.9.1 [29] on the basis of the crystal structure of *B. abortus* DXR-II (pdb: 3upy) [26].

To gain further insights into their putative functional impact, the amino acid changes detected as related to functional divergence of DXR-II were mapped onto the three-dimensional structure of *B. melitensis biovar abortus 2308* DXR-II in its apo form and in complex with the competitive inhibitor fosmidomycin (Figure 3) [26]. Predicted sites were mostly distributed through the middle catalytic domain, but some were also found in the COOH-terminal and NH2-terminal NADP-binding domains (Figure 3A). Two predicted sites corresponded to the conserved residues 229 and 320, identified as important for DXR-II activity [26]. Thr229, together with Lys191 and Lys193, serve to anchor fosmidomycin, presumably participating in the proper binding of the substrate (Figure 3B). Arg320 is located in a cavity at the dimer interface and, together with positions Glu174, Phe178 and Tyr322, may be involved in interactions between the two subunits of the DXR-II dimer (Figure 3C).

### DXR-IIs show a discontinuous taxonomic, metabolic and phenotypic distribution among eubacteria

The markedly scattered distribution of sequences belonging to the DXR-II family across higher order eubacterial taxonomic groups was previously observed [23]. In this up-to-date survey, DXR-IIs were found as encoded by the genomes of free-living eubacteria strains mostly from Alphaproteobacteria (26 strains, mainly from the genera *Brucella*, 11, and *Bartonella*, 6) and Firmicutes (21 strains, mainly from the genus Listeria, 9). However, genes coding for functional DXR-II representatives were also found in the genomes of three additional distantly related bacterial taxonomic lineages i.e. the Chloroflexi, Betaproteobacteria and Fusobacteria (Figure 2). Within the DXR-II family, Alphaproteobacteria, Firmicutes and Chloroflexi sequences clustered into separate subclades, while the single Betaproteobacteria and Fusobacteria representatives grouped within the Alphaproteobacteria and Firmicutes subclades, respectively (Figure 2).

We examined the distribution of functional DXR-II at lower taxonomical levels. For example, the occurrence of discontinuities was evident when we mapped DXR-II onto a tree depicting the evolutionary relationships of 72 alphaproteobacterial species (Additional file 7) [30]. *DXR-II* genes could only be found in the genomes of 25 strains among the 64 with fully sequenced genomes represented in the tree. They mainly belong to the order Rhizobiales, although significant hits were also retrieved from other taxonomic ranks, such as Rhodospirillales or Rhodobacteraceae. Within these alphaproteobacterial groups, strains whose genomes contained genes both encoding and not encoding DXR-II and/or DXR-I could be found. Discontinuities in DXR-II distribution could be appreciated with, e.g., the closely related pairs of Rhodospirillales species *Magnetospirillum magneticum* AMB-1*/Rhodospirillum rubrum* ATCC 11170 and *Acidiphilium cryptum* JF-5*/Gluconobacter oxydans* 621H. More strikingly, we have retrieved a DXR-II sequence only in one out of the five examined genomes of strains from *Rhodopseudomonas palustris* (strain BisB5), a feature perhaps related to the metabolical versatility attributed to this species [31] (Additional file 7). A similar patchy distribution of DXR-II was observed when DXR-II and DXR-I were mapped onto a phylogeny of Firmicutes (Additional file 8) [32].

Searches for enzymes of the MEP and MVA pathways of IPP and isoprenoid biosynthesis were also performed (Additional file 6: Table S2). The 51 DXR-II-containing eubacterial strains were classified according to the distribution of enzymes of these pathways, revealing the occurrence of multiple patterns (Figure 2 and Additional file 6: Table S3). The majority of surveyed eubacterial genomes contained genes coding for enzymes of the MEP pathway, but a significant number of them had lost one or more of these enzymes. DXR-I would have been preferentially lost among Alphaproteobacterial strains, but some losses were also found in Firmicutes and Chloroflexi (class A). These species would then exclusively rely on DXR-II for IPP biosynthesis through the MEP pathway. A group, mainly composed of Firmicutes strains showed genes encoding both DXR-II and DXR-I (class B). A significant number of genomes also encoded for enzymes of the MVA pathway. Some of these strains would then use solely the MVA pathway for isoprenoid biosynthesis, such as the two Chloroflexi representatives (class C). DXR-II activity has been experimentally shown from one of these strains, *Chloroflexus* auranticus J-10-fl, by complementation assays (Additional file 5). Most of them also have a complete and functional MEP pathway, such as *Listeria monocytogenes* (class D) [6]. Finally, in the genomes of two Firmicutes strains (*Anaerococcus prevotii* DSM 20548 and *Finegoldia magna* ATCC 29328) no genes encoding enzymes from the MEP (apart from DXR-II) or the MVA pathways could be found (class E). Interestingly however, DXR-II activity had been confirmed experimentally for the latter [23].

Similarly, the distribution of DXR-II was compared to that of enzymes of the CP pathway of amino acid biosynthesis. The CP represents three enzymatic steps. The first is the phosporylation of aspartate, carried out by AK leading to β-aspartyl-phosphate, which in turn is oxidized by an ASDH to aspartate semialdehyde. Subsequently, HD catalyses the reduction of aspartate beta-semialdehyde into homoserine, in the third and last step of the CP pathway (Figure 1). The evolutionary diversification of enzymes of the CP in bacteria is known to have been shaped by gene duplication and fusion events, resulting in bifunctional AK_HD proteins [33]. Most genomes of the 51 DXR-II-containing strains encoded AK and HD. The genomes of five strains also showed bifunctional *AK_HD* genes, while the genomes of only three Alphaproteobacteria strains encoded for ASDH and were believed to have functional CP (class B) (Figure 1 and Additional file 6: Table S3). However, none of the genomes of DXR-II-containing strains encoded the complete set of enzymes of the CP (class A, AK, HD, AK_HD and ASDH).

We next examined the distribution of biological properties across DXR-II-containing bacterial strains. For this purpose, we projected the data contained in the NCBI’s Microbial Organism Information Page onto the original set of 1489 bacterial strains, after correcting for ambiguities and redundancies. The database, available for download at http://ftp.ncbi.nlm.nih.gov/genomes/genomeprj_archive/, included categories related to the ecological requirements of the organism (e.g. habitat, oxygen requirement, salinity, temperature range, optimal temperature), morphological features (e.g., shape, arrangement, endospores and motility) and additional phenotypic traits (e.g., Gram stain, dinucleotide GC content, genome size and pathogenicity). The distribution of properties across DXR-II- and non DXR-II-containing eubacterial strains is shown in Table 3. To test whether any of these biological properties were differentially represented in the subset of 51 eubacterial strains containing DXR-II regarding the remaining non-DXR-II harbouring strains, we performed Fishers’ exact tests. According to these tests, none of the categories related to the ecological requirements of the organism showed a biased representation among DXR-II-containing strains, suggesting that these organisms may not live in shared habitats. A similar unbiased pattern of distribution was found for additional morphological and phenotypic features (Table 3). Only the category “pathogenic in animals” showed a significant overrepresentation among DXR-II-containing strains (Table 3). Similarly, for quantitative properties, such as genome size, GC content and optimal growth temperature, a Student’s T test was performed to assess significance of the differences between means. Again, none of the tests were significant (Table 3).

**Table 3:** Distribution of biological properties in DXR-II and non-DXR-II containing bacterial strains and statistical tests of enrichment

**Biological properties**		**Number of strains**		**p-value**
		**DXR-II**	**Non-DXR-II**	
**Habitat**		41	1166	
	Host-associated	18	383	0.36
	Multiple	16	330	0.33
	Specialized	3	148	ND
	Terrestrial	2	94	ND
	Aquatic	2	211	ND
**Oxygen Req**		39	1137	
	Facultative	15	404	0.76
	Aerobic	15	413	0.88
	Anaerobic	9	284	ND
**Salinity**		7	245	
	Non-halophilic	6	171	ND
	Moderate halophilic	1	30	ND
**Temp. range**		38	1202	
	Mesophilic	36	1013	0.64
	Thermophilic	2	107	ND
**Optimal temp. **^**a**^		38.61 (18)	41.21 (555)	0.27
**Genome Size **^**a**^		3.73 (48)	3.59 (1456)	0.50
**GC Content **^**a**^		48.23 (45)	48.63 (1193)	0.84
**Shape**		43	1239	
	Rod	29	794	0.90
	Coccobacillus	6	21	ND
	Coccus	5	188	ND
	Filament	2	20	ND
	Short rod	1	2	ND
**Arrangment**		35	899	
	Singles	17	501	0.77
	Pairs	9	209	ND
	Chains	4	107	ND
	Groups	3	3	ND
	Filaments	2	22	ND
**Endospores**		18	626	
	Yes	6	121	ND
	No	12	505	0.71
**Motility**		27	947	
	Yes	22	579	0.37
	No	5	365	ND
**Gram Stain**		39	1050	
	-	22	704	0.60
	+	17	344	0.35
**Pathogenic in**		42	1008	
	Animal	15	181	**0.04**
	Human	14	264	0.50
	No	13	521	0.11

P-values resulting from Fisher’s exact tests are shown for categories represented in at least 10 bacterial strains. Test significant at p < 0.05 is shown in bold type. ^a^, for these quantitative properties, the average value (number of strains is shown between parentheses) and p-values resulting from Student’s T tests performed to assess significance of the differences between means are shown.

### Comparative sequence-based analysis of HGT in DXR-II evolution

The markedly discontinuous phylogenetic distribution shown by DXR-II might be explained by recurrent events of HGT occurring between unrelated bacterial strains. So long as the DXR-II sequence retains sequence features of the donor strain significantly distinct from that of the genome of the recipient strain, they could be inferred as being acquired by HGT. Consequently, comparative nucleotide sequences analyses of DXR-II against their host genomes could yield clues about their origin and the putative role of HGT in the distribution of DXR-II across eubacteria.

Several methods and criteria were applied to identify signatures of HGT (please see Methods for a complete description). Firstly, GC content at the three codon positions, as well as the total, was estimated. As previously observed [34,35], GC content was relatively constant among genes of a particular species’ genomes, although displaying wide variation among species (Additional file 6: Table S4). This was particularly evident at the third codon position, as the majority of these sites are synonymous and, consequently, differences due to mutational biases are higher. In contrast, the first and second codon positions appear to be more conserved between genomes and are, consequently, less informative (Additional file 6: Table S4). The GC contents of all DXR-II coding sequences were compared to the mean for all genes encoded by the corresponding genomes. DXR-II from both Chloroflexi representatives and the single Fusobacteria representative *Sebaldella termitidis* ATCC 33386 showed significantly lower GCt and GC3 content regarding the respective mean for all genes in the genome (Additional file 6: Table S4). A fourth bacterial strain, *Rhizobium* NGR234, showed higher GCt and GC3 content (Additional file 6: Table S4).

Secondly, we examined for biases in dinucleotide relative frequencies, a remarkably stable property of the DNA of an organism claimed to constitute a ‘genomic signature’ that can discriminate sequences from different organisms [36]. We focused on the dinucleotide biases at third and first (3:1) codon positions, which are less sensitive to selective constrains [37]. Consequently, the 3:1 dinucleotide frequencies were calculated for all DXR-II coding sequences and for the entire set of genes in the corresponding genomes. They both showed significant variation across organisms, and therefore could be used as such genomic signatures. Significance of the differences between *DXR-II* genes and their genomes were examined by calculating the dinucleotide relative abundance difference or σ difference (Additional file 6: Table S5) [36]. Pairwise co-variation was further assessed through the Spearman and Kendall rank tests (Additional file 6: Table S5). In all but one example, both Spearman’s ρ and Kendall’s τ correlation coefficients indicated strong positive correlation. An exception was provided by *Halanaerobium hydrogeniformans*, which showed negative correlation. All tests revealed significant covariation of 3:1 dinucleotide frequencies of DXR-II with the frequencies of the corresponding genomes, contrary to the expectations of HGT.

Next, we estimated relative synonymous codon usages (RSCU) values, which provide with a simple effective measure of synonymous codon usage bias. Differences in RSCU between *DXR-II* genes and all other genes in each corresponding genome were assessed by means of χ^2^ tests (Additional file 6: Table S6) [34]. Chloroflexi strains and *S. termitidis ATCC 33386* showed the higher χ^2^ statistic values, revealing higher variation. However, none of the tests was significant, indicating that *DXR-II* genes have a codon usage patterns consistent with that of their corresponding genomes, and therefore unlikely to reflect HGT.

Finally, we examined the degree of bias in codon usage of *DXR-II* genes towards the codon usage of the most expressed genes by comparing Codon Adaptation Index (CAI) values. A significant deviation from the average CAI of the genome was found in strains of Chloroflexi and *S. termitidis ATCC 33386* (Additional file 6: Table S7)*.*

## Discussion and conclusions

The structural and functional diversity of isoprenoids correlates with the existence of a wide biochemical and genetic plasticity for their biosynthesis [17]. In eubacteria, this is commonly achieved through the use of alternative metabolic pathways and enzymatic steps in specific lineages. Interesting examples are provided by HMGR and IDI, which are encoded by at least two distinct gene families in bacteria. In this paper we focus in DXR-II, recently characterized as an alternative family to DXR-I in performing the second step of the MEP pathway of isoprenoid biosynthesis in a selected group of eubacteria [23].

Apart from the NAD-binding domain with a core Rossmann-type fold found at the N-terminal region of all oxidoreductases, no significant similarity at the sequence level was observed between DXR-I and DXR-II to infer homology [23]. Correspondingly, the recent determination of the DXR-II crystal structure showed only slight structural relationship with DXR-I proteins and revealed a unique arrangement of the active site [26]. Examples of enzymes catalyzing identical reactions through the same catalytic mechanisms but showing structurally unrelated active sites are known outside the isoprenoid field [38-41]. In some of these though, key catalytic residues may be conserved between functionally redundant enzymes, as also reported for DXR-I and DXR-II [26]. DXR-I and DXR-II likely represent analogous genes that evolved redundant biochemical functions through mechanistic convergence.

Our results support the emergence of the DXR-II family through type I, but not type II, functional divergence from DLO1 and DLO2 families of previously uncharacterized oxidoreductases. These data suggest that DXR-II acquired additional structural and/or functional constraints rather than shifted constraints in amino acids that were already ancestrally constrained. Amino acid changes critical for functional divergence and acquisition of DXR-II biochemical activity were predicted, many of them corresponding to positions highly conserved in DXR-II, but otherwise variable in DLO1 and/or DLO2. Interestingly, two of these predicted amino acids, Thr229 and Arg320, had been previously identified for their role in fosmidomycin/substrate binding and in dimerization, respectively [26], suggesting that functional shifts in a limited number of amino acid positions could be at the origin of the acquisition of DXR-II biochemical activity.

It could be assumed that the MEP pathway is the ancestral route for IPP and isoprenoid biosynthesis in eubacteria, including the membrane-associated hopanoids, which are among the oldest known biomolecules [42]. The entire set of genes encoding for enzymes involved in the MEP pathway, including DXR-I, has been found widespread in all eubacterial taxonomic groups [5]. In a significant number of DXR-II-containing eubacterial genomes (31), including those from closely related strains, DXR-I has been lost. This raises the question of how DXR-II evolved in DXR-I containing strains, as acquisition of redundant biochemical activities should not be favoured by evolution. The DXR-II family could have emerged under an ecological context that conferred a selective advantage to the emergence and maintenance of a functionally redundant enzyme, e.g. when gene dosage is selectively advantageous. Due to the wide and diverse functions played by isoprenoids and their essential role for cell viability, critical situations in which their biosynthesis was absolutely required may have occurred multiple times throughout eubacterial evolution. Emergence of the DXR-II family should have occurred at an early time in evolution, as supported by the scattered distribution of DXR-II and related oxidoreductases from DLO1 and DLO2 families in distantly related lineages of eubacteria. After relaxation of that burst in selective constraints for isoprenoid biosynthesis, some strains could then have lost one redundant enzyme, commonly DXR-II, which shows less catalytic activity in vitro [26]. In addition, maintenance of DXR-II, which shows less sensitivity to inhibition by fosmidomycin than DXR-I [26], might have provided a selective advantage in bacterial strains sharing the same ecological niches as those naturally producing the antibiotic (e.g. *Streptomyces* species [43]).

The taxonomic distribution of DXR-II across eubacteria showed a marked discontinuity, which was also verified at the metabolic and phenotypic level. Although most genes encoding DXR-II were found in eubacteria with the MEP pathway, their occurrence was not linked to a unique pattern of distribution of enzymes of the MEP or MVA pathways. Similarly, HD, the oxidoreductase family that showed the highest level of similarity with DXR-II, was found in most DXR-II-containing bacterial strains, but not all. In addition, examination of the distribution of biological properties across DXR-II-containing strains showed maintenance of DXR-II in the genomes was not linked to a unique pattern of ecological or phenotypic traits. The only exception was ‘pathogenic in animals’, significantly enriched among DXR-II-containing strains, reflecting the occurrence of DXR-II among pathogenic strains of *Brucella*, *Bartonella*, *Listeria* and *Clostridium*[44-47].

The outstanding phylogenetic discontinuity in DXR-II distribution across eubacteria could be explained through two alternative, though not mutually exclusive, evolutionary mechanisms, i.e., gene gain through HGT or gene loss. HGT is known to have shaped the evolution of multiple metabolic pathways, including IPP and isoprenoid biosynthesis [8,24,48]. However, a unique event of HGT cannot properly explain DXR-II phylogeny. According to our phylogenetic analysis, such HGT events should instead have occurred at different time points throughout eubacterial evolution, e.g. between the Alphaproteobacteria and Firmicutes phyla, between the Alphaproteobacteria and Betaproteobacteria classes within the proteobacteria phylum, between Firmicutes and specific Chloroflexi strains or between Firmicutes and specific Fusobacteria. More recently, HGT should also have occurred between closely related Alphaproteobacteria or Firmicute strains. If this was the case, HGT events should have left a signature of atypical sequence features in *DXR-II* genes, provided they were recent enough and occurring between distantly taxonomically related donor and acceptor bacterial strains [34,35]. Weak signatures of HGT were found only in Chloroflexi and the Fusobacterium *S. termitidis ATCC 33386* at the level of GC content and CAI values. However, no biases in dinucleotide frequencies or codon usage were observed in any strain comparison. These results suggested that HGT events were not at the origin of all discontinuities, or were so ancient that *DXR-II* genes ameliorated their sequence to specific base composition and codon usage of the host genome, making them indistinguishable from ancestral sequences [34,35].

Consequently, although old episodic events of HGT cannot be excluded, the alternative hypothesis of recurrent *DXR-II* (or eventually *DXR-I*) gene loss is more likely to explain DXR-II phylogeny. This mechanism has been traditionally considered less parsimonious, as it involves a complex ancestor and gene loss events occurring independently at multiple evolutionary lineages. However, recent works suggests that, on average, gene loss might be a more likely event than gene gain through HGT [49-51].

The DXR-I/DXR-II model constitutes an exceptional natural model to experimentally test the emergence and maintenance of redundant gene function between non-homologous genes as a result of convergent evolution, as opposed to their emergence from intragenomic duplicates, or paralogs. Furthermore, our results highlight the importance of the functional characterization of evolutionary shortcuts in isoprenoid biosynthesis for screening specific antibacterial drugs and for regulating the production of isoprenoids of human interest.

## Methods

### Sequence and phylogenetic analysis

Sequence databases from the whole sequenced genomes of 1489 bacterial strains were downloaded from the NCBI. Orthologs of enzymes from the MEP and MVA pathways for IPP biosynthesis, as well as for enzymes of the CP of amino acid biosynthesis (Figure 1), were defined as the best reciprocal hits resulting from all-against-all local BLASTP-searches with an E-value cutoff of 1E-5 and a bit score cutoff of 50 [52] using selected previously characterized sequences as queries (Additional file 6: Table S2). Only hits corresponding to full-length sequences were considered. Resulting hits were scanned for the presence of INTERPRO domains.

Phylogenetic analysis was performed on the basis of an alignment of protein sequences obtained using MUSCLE [53]. Maximum Likelihood (ML) phylogenetic reconstruction was carried out in PhyML v3.0 [54] using the LG protein evolution model [55] and heterogeneity of amino acid substitution rates corrected using a γ-distribution (G) with eight categories plus a proportion of invariant sites (I), selected by ProtTest v2.4 as the best-fitting amino acid substitution model according to the Akaike information criterion [56]. Starting phylogenetic trees were constructed using the modified program BIONJ. Tree topology searching was optimized using the subtree pruning and regrafting option. The statistical support of the retrieved topology was assessed using the Shimodaira-Hasegawa-like approximate likelihood ratio test (aLRT) [57].

Bayesian analysis was conducted in MrBayes v3.1.2 [58] using the WAG model [59] plus G with eight categories plus I. Searches were run using four Markov (MCMC) chains of length 1000000 generations sampling every 100th tree. Once stationary phase was reached (determined by the average standard deviation of split sequences approaching 0, which reflects convergence of independent tree samples), the first 2500 trees were discarded as burn-in, and a 50% majority-rule consensus tree was then constructed to evaluate Bayesian posterior probabilities on clades. Neighbor Joining phylogenetic analysis was performed in MEGA 5.0 [60]. The evolutionary distances for Neighbor Joining phylogenetic reconstruction were computed using the Poisson correction method. To obtain statistical support on the resulting clades, a bootstrap analysis with 1000 replicates was performed. Resulting trees were represented and edited using FigTree v1.3.1.

### Analysis of functional divergence

The analysis of functional divergence was performed using DIVERGE v2.0 [61]. DIVERGE performs the ML estimation of the theta (θ) type-I and type-II coefficients of functional divergence, based on the occurrence of altered selective constraints or radical shifts of physicochemical properties, respectively [27,28]. θ value indicates the extent of functional divergence, ranging from 0, no functional divergence to 1, representing maximum divergence. Functional divergence can be explicitly tested by comparing the fit of a model allowing for functional divergence versus a null model in which functional divergence is not permitted (θ = 0). A Likelihood Ratio Test (LRT) is then used to examine the significance of differences between the lnL values of the two nested models (calculated as 2ΔLnL -twice the difference between their lnL values) [62]. As the LRT asymptotically follows a χ^2^ distribution with a number of degrees of freedom equal to one, i.e. the differences in number of parameters between the models being compared (θ), a p-value for the fitting of the model accounting for functional divergence can be computed. DIVERGE also uses a site-specific profile to estimate the posterior probabilities (Q_k_) of individual amino acid sites to be critical for functional divergence.

### G + C% content, dinucleotide frequencies, codon usage, and CAI analyses

The following sequence features i) GC% content at three codon positions and total (GC1, GC2, GC3 and GCt), ii) dinucleotide frequencies at 3:1 codon sites (third base and first base of the succeeding codon) and iii) the relative synonymous codon usages (RSCU) were extracted for individual DXR-II sequences and the rest of genes in the corresponding genomes through PERL and R scripts using cpan and bioperl modules. Codon Adaptation Indexes (CAI) [63] for individual genes and genomes were calculated using the method depicted in [64] as implemented in DAMBE software [65]. Comparative analyses of these sequence features between *DXR-II* genes and the rest of genes in the genome were performed and differences assessed using different statistical tests.

i) Differences in G and C nucleotides content were considered as significant when GC% deviated by two or more standard errors (SEs) regarding the respective means for all genes in the genome or deviations at first and third codon position were of the same sign and at least one was higher two or more SEs [35,66].

ii) Dinucleotide relative frequencies were calculated as:

$$\rho_{\mathrm{XY}}^{*}=\frac{f_{\mathrm{XY}}}{f_{X}f_{Y}}$$

Where *f*_X_ denotes the frequency of the mononucleotide X and *f*_XY_ the frequency of the dinucleotide XY. The array of *ρ*_XY_ dinucleotide values define the genomic signature of a given species’ genome [36]. A simple way to compute differences in dinucleotide relative frequencies between a given gene (*f*) and the value of the entire genome (*g*) is through the absolute difference (σ difference) calculated as:

$$\sigma^{*}\left(f,g\right)=\frac{1}{16}\sum_{\mathrm{XY}}\left|\rho_{\mathrm{XY}}^{*}\left(a\right)-\rho_{\mathrm{XY}}^{*}\right)\left(\left(b\right)\right|$$

averaged over all 16 dinucleotides [67]. Furthermore, pairwise covariation of the 3:1 dinucleotide differences were assessed using the Spearman’s rank correlation coefficient ρ [68] and the Kendall’s rank correlation coefficient τ [69]. Both are nonparametric statistics allowing testing for dependence between two variables.

iii) RSCU provides with a simple effective measure of synonymous codon usage bias, in which codon frequencies are normalized by the frequency expected under the assumption of equal usage of synonymous codons for a given amino acid [70].

$$\mathrm{RSC}U_{i}=\frac{X_{i}}{\frac{1}{n}\sum_{i=1}^{n}X_{i}}$$

For synonymous codon i of an n-fold degenerate amino acid, where X is the number of occurrences of codon i, and n the number of synonymous codons encoding for a given amino acid i.e. 1, 2, 3, 4, or 6. In the absence of any codon usage bias (i.e. all synonymous codons are used equally), the RSCU value would be 1. A codon that is used less or more frequently than expected will have an RSCU value < or > than 1, respectively. Start, stop and tryptophan codons were excluded from the analysis. To measure bias in synonymous codon usage between *DXR-II* and all genes in the genome, a χ^2^ test of RSCU with 41 degrees of freedom was implemented [34].

iv) CAI was used as an alternative method to determine the degree of bias in the synonymous codon usage of the *DXR-II* gene regarding the optimal codon usage in the genome [34,63]. RSCU was firstly determined for all genes in each species genome, and subsequently used as reference set for the frequencies of the optimal codons in each species [65]. CAI is calculated as

$$\mathrm{CAI}=\frac{\mathrm{CA}I_{\mathrm{obs}}}{\mathrm{CA}I_{\max}}$$

where CAI_obs_ is the mean of the RSCUs for all codons in a particular gene, and CAI_max_ is the mean of the RSCU for the most frequently used codons for an amino acid in a genome. CAI ranges from 0 to 1, being 1 if the gene only uses the most frequently used synonymous codons in the reference set. Differences in CAI between *DXR-II* and all genes in the genome were considered as significant if higher than 1.5 times the SE.

## Availability of supporting data

The multiple sequence alignment and the phylogenetic tree-files supporting the results of this article have been deposited and are publicly available in the TreeBASE repository under accession numbers: S14611 (http://purl.org/phylo/treebase/phylows/study/TB2:S14611).

## Abbreviations

AK: Aspartokinase; ASDH: Aspartate semialdehyde dehydrogenase; CAI: Codon adaptation index; CP: Common pathway; HGT: Horizontal gene transfer; DLO: DXR-II-Like oxidoreductases; DXR: DeoxyXylulose 5-phosphate reductoisomerase; DMAPP: DiMethylAllyl diphosphate; DXR like: DXR-II; HD: Homoserine dehydrogenase; HMGR: Hydroxy-3-Methyl-Glutaryl-CoA Reductase; IDI: IPP isomerase; IPP: Isopentenyl diphosphate; LRT: Likelihood ratio test; MEP: methylerythritol 4-phosphate; MVA: Mevalonate; ML: Maximum likelihood; RSCU: Relative synonymous codon usage; UID: (taxonomy) Unique Identifier.

## Competing interests

The authors declare that they have no competing interests.

## Authors’ contributions

LCP and AL collected data. LCP, AL and JPG analysed data. LCP, AL, JPG, FJS, VAA and MRC contributed to the interpretation of the data. LCP and MRC conceived the study and participated in its design. LCP wrote the manuscript with significant contributions by JPG, FJS, VA and MRC. All authors have read and approved the final manuscript.

## Supplementary Material

Additional file 1 **Multiple alignment of 130 DRL and DLO related protein sequences.** Positions conserved in 100%, 70% or 40% of the sequences are shown in black, dark grey and light grey, respectively. Strain names are grouped as DXR-II, DLO1 (grey shadow) and DLO2. Click here for file

Additional file 2 **ML phylogeny of DXR-II and DLO related sequences.** ML cladogram depicting the evolutionary relationships among 53 DXR-II and 77 related protein sequences. Statistical support for clades (ML aLRT support values) is indicated next to nodes. Click here for file

Additional file 3 **Bayesian phylogeny of DXR-II and DLO related sequences.** Bayesian cladogram depicting the evolutionary relationships among 53 DXR-II and 77 related protein sequences. Statistical support for clades (posterior probabilities) is indicated next to nodes. Click here for file

Additional file 4 **Neighbor Joining phylogeny of DXR-II and DLO related sequences.** Neighbor Joining cladogram depicting the evolutionary relationships among 53 DXR-II and 77 related protein sequences. Statistical support for clades (bootstrap values) is indicated next to nodes. Click here for file

Additional file 5 **Complementation of DXR-deficient *E. coli* cells with putative DXR-II sequences from *Chloroflexus auranticus* J-10-fl.** The putative DXR-II sequences were PCR-amplified from genomic DNA and cloned into pJET1.2. The corresponding constructs and positive and negative controls (C-, empty vector; C+, DXR-II (YP_418479.1) from *B. melitensis* biovar *abortus* 2308) were used to transform EcAB4-10 cells [23]. Ability of the cloned gene to rescue growth of this DXR-deficient mutant strain was ascertained by monitoring growth on plates either supplemented (+) or not (−) with 1 mM MVA as indicated. 1) YP_001634831.1, 2) YP_001634944.1, and 3) YP_001636771.1. Click here for file

Additional file 6 **Table S1.** List of amino acid sites detected as related to functional divergence of DXR-II vs DLO1 and DXR-II vs DLO2. **Table S2.** List of sequences used as queries in BLAST searches for enzymes of the MEP, MVA and CP pathway, and the corresponding bacterial strain. **Table S3.** Distribution of enzymes of the MEP, MVA and the CP pathways across 128 whole sequenced bacterial strains. **Table S4.** GC content of *DXR-II* genes and corresponding genomes. **Table S5.** 3:1 relative dinucleotide frequencies at *DXR-II* genes and their corresponding genomes and statistical tests of co-variation. **Table S6.** RSCU values at *DXR-II* genes and their corresponding genomes and statistical tests of independence. **Table S7.** CAI values for *DXR-II* genes and the average for all genes in the corresponding genomes. Click here for file

Additional file 7 **Distribution of DXR-I and DXR-II in Alphaproteobacteria.** The occurrence of DXR-I and DXR-II is represented for alphaproteobacterial strains with full sequenced genomes in a phylogenetic context, according to the robust species tree reported in [30]. Click here for file

Additional file 8 **Distribution of DXR-I and DXR-II in Firmicutes.** The occurrence of DXR-I and DXR-II is represented for strains with full sequenced genomes in a phylogenetic context, according to the robust species tree for Firmicutes reported in [32]. Click here for file
