# Supplementary material for: Evolutionary diversification and characterization of the eubacterial gene family encoding DXR type II, an alternative isoprenoid biosynthetic enzyme
Source: BMC Evol Biol. 2013 Sep 3;13:180. doi: 10.1186/1471-2148-13-180 (PMC3847144; doi:10.1186/1471-2148-13-180)
Supplement: Additional file 1 — Multiple alignment of 130 DRL and DLO related protein sequences. Positions conserved in 100%, 70% or 40% of the sequences are shown in black, dark grey and light grey, respectively. Strain names are grouped as DXR-II, DLO1 (grey shadow) and DLO2. [file 1471-2148-13-180-S1.pdf]

```
                                                          *        20         *        40         *        60         *        80         *
Anaerococcus_prevotii_DSM_20548                    : ------------------------------------MFKINRKLKEMKDNGE---S--IKLAIIGC-GKMGASLISQISK-MDAMEVKLVVDRTPQ :  53
Bacillus_clausii_KSM-K16                           : -------------------------------------MSIYRELQERDKQQQ---P--IKVGVIGA-GQMGFGLISQISR-IPGMVVGGICDVHIE :  52
Bacillus_halodurans_C-125                          : ------------------------------------MLGINRKLEQLESDGT---I--IRVGLVGA-GQMGRGMVSQIES-MKGMRVVVTADIVLE :  53
Bacillus_pumilus_SAFR-032                          : -------------------------------------MSIYQQLLARERSGD---P--IKVGIIGA-GQMGFGLISQISK-IPGMIVAGVCDIHLS :  52
Bartonella_bacilliformis_KC583                     : ------------------------------MASNVTLTGLARDLKQYAEQHH---P--IRIGLVGC-GEMGTDLLSSVAH-IEGITVAAVVTRTPL :  59
Bartonella_clarridgeiae_73                         : ------------------------------MTSNINLTGLARDLKQRAETYK---P--IRIGLIGC-GEMGTDLLSSITH-IDGITIAAVATRTPS :  59
Bartonella_grahamii_as4aup                         : ------------------------------MASNVNLTGLAHDLKQRAEKHP---P--IRIGLIGC-GEMGTDLLSSIAH-MDGITVAAVATRTPS :  59
Bartonella_henselae_str._Houston-1                 : ------------------------------MASNVSLTGLAHDLKQRAENHP---P--IRIGLIGC-GEMGTDLLSSIAH-MDGITVAAVATRTPS :  59
Bartonella_quintana_str._Toulouse                  : ------------------------------MASNVSLTGLARDLKQRAENHP---P--IRIGLIGC-GEMGTDLLSSIAH-MDGITVAAVATRTPS :  59
Bartonella_tribocorum_CIP_105476                   : ------------------------------MASNVNLTGLAHDLKQRAENHP---P--IRIGLIGC-GEMGTDLLSSIAH-MEGITVAAVATRTPS :  59
Brucella_abortus_bv._1_str._9-941                  : ------------------------------MTTNVALVGLARDLAARAETGK---P--IRIGLIGA-GEMGTDIVTQVAR-MQGIEVGALSARRLP :  59
Brucella_abortus_S19                               : ------------------------------MTTNVALVGLARDLAARAETGK---P--IRIGLIGA-GEMGTDIVTQVAR-MQGIEVGALSARRLP :  59
Brucella_canis_ATCC_23365                          : ------------------------------MTTNVALVGLARDLAARAETGK---P--IRIGLIGA-GEMGTDIVTQVAR-MQGIEVGALSARRLP :  59
Brucella_melitensis_ATCC_23457                     : ------------------------------MTTNVALVGLARDLAARAETGK---P--IRIGLIGA-GEMGTDIVTQVAR-MQGIEVGALSARRLP :  59
Brucella_melitensis_biovar_Abortus_2308            : ------------------------------MTTNVALVGLARDLAARAETGK---P--IRIGLIGA-GEMGTDIVTQVAR-MQGIEVGALSARRLP :  59
Brucella_melitensis_bv._1_str._16M                 : ----------------------------------MALVGLARDLAARAETGK---P--IRIGLIGA-GEMGTDIVTQVAR-MQGIEVGALSARRLP :  55
Brucella_microti_CCM_4915                          : ------------------------------MTTNVALVGLARDLAARAETGK---P--IRIGLIGA-GEMGTDIVTQVAR-MQGIEVGALSARRLP :  59
Brucella_ovis_ATCC_25840                           : ------------------------------MTTNVALVGLARDLAARAETGK---P--IRIGLIGA-GEMGTDIVTQVAR-MQGIEVGALSARRLP :  59
Brucella_pinnipedialis_B2/94                       : ------------------------------MTTNVALVGLARDLAARAETGK---P--IRIGLIGA-GEMGTDIVTQVAR-MQGIEVGALSARRLP :  59
Brucella_suis_1330                                 : ------------------------------MTTNVALVGLARDLAARAETGK---P--IRIGLIGA-GEMGTDIVTQVAR-MQGIEVGALSARRLP :  59
Brucella_suis_ATCC_23445                           : ------------------------------MTTNVALVGLARDLAARAETGK---P--IRIGLIGA-GEMGTDIVTQVAR-MQGIEVGALSARRLP :  59
Chelativorans_sp._BNC1                             : ------------------------------MVTNVALVGLARDLEERAKAGK---P--IRIGLIGT-GEMGTDIVARISH-MRGIEVGAIAELRPK :  59
Chloroflexus_aurantiacus_J-10-fl                   : -------------------------------------MSLRERLIQYSQHQK---M--IRVGLVGA-GQMGVGFISQTER-MEGLRVVAVADIIPG :  52
Chloroflexus_sp._Y-400-fl                          : -------------------------------------MSLRERLIQYSQHQK---M--IRVGLVGA-GQMGVGFISQTER-MEGLRVVAVADIIPG :  52
Clostridium_difficile_630                          : ------------------------------------MLDLNRKLAKLEEEGK---K--IKVALIGA-GHMGNGMVSQMAN-MKGIEASIVVDINLE :  53
Clostridium_difficile_CD196                        : ------------------------------------MLDLNRKLAKLEEEGK---K--IKVALIGA-GHMGNGMVSQMAN-MKGIEASIVVDINLE :  53
Clostridium_difficile_R20291                       : ------------------------------------MLDLNRKLAKLEEEGK---K--IKVALIGA-GHMGNGMVSQMAN-MKGIEASIVVDINLE :  53
Eubacterium_limosum_KIST612                        : ------------------------------------MINMMYKLQKLEDEGK---P--IKASIVGA-GQMGCGMAAQMTV-MKGMDPVVVVDVVLE :  53
Finegoldia_magna_ATCC_29328                        : ------------------------------------MNKMQRKLVDLDNHGQ---K--VRVGLVGC-GKMGSGLVSQLSR-IKGMRPSVIIDRHVD :  53
Halanaerobium_hydrogeniformans                     : ------------------------------------MSNVYKDLKELEAKGE---Y--IKIGLIGA-GQMGTDIVSQVAQ-MNGVKVPVVADIDIE :  53
Listeria_innocua_Clip11262                         : -------------------------------------MTLYRQLLAREKENN---P--IRVGVIGA-GQMGFGMISQIAA-IPGMSVVGISDIHVD :  52
Listeria_ivanovii                                  : -------------------------------------MTLYRQLLAREQENN---P--IRVGVIGA-GQMGFGMISQIAA-IPGMSVVGISDIHVE :  52
Listeria_monocytogenes                             : -------------------------------------MTLYRQLLARENENN---P--IRVGVIGA-GQMGFGMISQIAA-IPGMSVVGISDIHVE :  52
Listeria_monocytogenes_08-5923                     : -------------------------------------MTLYRQLLARENENN---P--IRVGVIGA-GQMGFGMISQIAA-IPGMSVVGISDIHVE :  52
Listeria_monocytogenes_EGD-e                       : -------------------------------------MTLYRQLLARENENN---P--IRVGVIGA-GQMGFGMISQIAA-IPGMSVVGISDIHVE :  52
Listeria_monocytogenes_HCC23                       : -------------------------------------MTLYRQLLARENENN---P--IRVGVIGA-GQMGFGMISQIAA-IPGMSVVGISDIHVE :  52
Listeria_monocytogenes_serotype_4b_str._           : -------------------------------------MTLYRQLLARENENN---P--IRVGVIGA-GQMGFGMISQIAA-IPGMSIVGISDIHVE :  52
Listeria_monocytogenes_serotype_4b_str._           : -------------------------------------MTLYRQLLARENENN---P--IRVGVIGA-GQMGFGMISQIAA-IPGMSIVGISDIHVE :  52
Listeria_welshimeri_serovar_6b_str._SLCC           : -------------------------------------MTLYRQLLAREKENN---P--IRVGVIGA-GQMGFGMISQIAA-IPGMSVVGISDIHVE :  52
Mesorhizobium_ciceri_biovar_biserrulae_W           : ------------------------------MASNVSLTGLARDLEERAKSGK---P--IRIGLIGS-GEMGTDIVTRVAH-MSGIEIGAISELNLP :  59
Mesorhizobium_loti_MAFF303099_1                    : ------------------------------MASNISLTGLARDLDERGKSGK---P--IRIGLIGS-GEMGTDIVTRVAH-MSGIEIGAISELNLP :  59
Mesorhizobium_loti_MAFF303099_2                    : ------------------------------------MTGLARDLASRAAEGR---P--VRIGVIGS-GEMGTDLVTQGML-MPGISVCAVSTRRPH :  53
Mesorhizobium_opportunistum_WSM2075                : ------------------------------MASNVSLTGLARDLEERGKSGK---P--IRIGLIGS-GEMGTDIVTRVAH-MSGIEIGAISELNLP :  59
Ochrobactrum_anthropi_ATCC_49188_1                 : ------------------------------MTTNVALVGLARDLAARAETGK---P--IRIGLIGA-GEMGTDIVTQVAR-MQGIEVGALSARRLP :  59
Ochrobactrum_anthropi_ATCC_49188_2                 : ------------------------------MATNVALTGLARDMQARADSGR---P--IRIGLIGS-GEMGTDIVTRVAH-MPGIEVGAISELRVP :  59
Pelagibacterium_halotolerans_B2                    : --------------------------------MTISKTGIARDLERLRDKGK---P--VRLGIIGS-GEMGTDLVTQVSL-MAGIEIAAIATRRPH :  57
Roseobacter_litoralis_Och_149                      : ------------------------------MAANISLTGLARDLSERAATGK---P--VRIGLIGS-GEMGTDIVTRAGM-MDGVEVAAISEVNPA :  59
Sebaldella_termitidis_ATCC_33386                   : ------------------------------------MLGLNYKLEKLEKAGT---P--ILTGIIGA-GQMGQGMVSQMYL-MEGMNPAIVVDINTE :  53
Sinorhizobium_fredii_NGR234                        : ------------------------------MPINVAPTGLTRDLMRREEGGR---P--IRIGLIGS-GEMGTDIVTRVAH-MPGIEIGAICDLNVG :  59
Starkeya_novella_DSM_506                           : ----------------------------MLGANGIVRSGIAAELEQWTAKHG---P--VTIGLAGA-GQMGTDLIVQAAL-MPGVRIGAVVEMNPQ :  61
Tepidanaerobacter_sp._Re1                          : ------------------------------------MLNINTRLRKLDESGE---K--INVGLVGV-GQMGRGIVTQVLK-MKGMNIPIIANRTVD :  53
Thermosediminibacter_oceani_DSM_16646              : ------------------------------------MLNLNSKLRELDASGK---K--INVGLIGA-GQMGRGMVSQIFC-MKGIRVAAICDRKLE :  53
Verminephrobacter_eiseniae_EF01-2                  : -------------------------------MDGFIPSGLAADLQARGLSGK---P--IRIGLIGS-GMMGGDIVTQVLQ-MRGIVVAAIADIHLP :  58
Anabaena_variabilis_ATCC_29413                     : ------------------------------------MIIIDNALKARAAAGN---P--VKVGMIGA-GFMGRGIANQIINSVPGMELVAISNRSID :  54
Chloroflexus_aggregans_DSM_9485                    : ------------------------------------MILVDTALARAETEGR---P--IRVGMIGA-GFMARGIALQIIRYTRGMRLVAIANRTIE :  54
Coraliomargarita_akajimensis_DSM_45221             : ------------------------------------MIIVDTRLKAREAAGN---P--IQVGILGA-GEMSLGLVNQIERHIPGMRVAAIYNRTLE :  54
Coxiella_burnetii_CbuG_Q212                        : ---------------------------------------MLNELQYREKTGS---P--IRVGLIGA-GSMGTGIAWQIGR-TPGMRLAFIAATTME :  50
Coxiella_burnetii_CbuK_Q154                        : ---------------------------------------MLNELQYREKTGS---P--IRVGLIGA-GSMGTGIAWQIGR-TPGMRLAFIAATTME :  50
Coxiella_burnetii_Dugway_5J108-111                 : ---------------------------------------MLNELQYREKTGS---P--IRVGLIGA-GSMGTGIAWQIGR-TPGMRLAFIAATTME :  50
Coxiella_burnetii_RSA_331                          : ---------------------------------------MLNELQYREKTGS---P--IRVGLIGA-GSMGTGIAWQIGR-TPGMRLAFIAATTME :  50
Coxiella_burnetii_RSA_493                          : ---------------------------------------MLNELQYREKTGS---P--IRVGLIGA-GSMGTGIAWQIGR-TPGMRLAFIAATTME :  50
Cyanothece_sp._PCC_7425                            : ------------------------------------MIIVDRALKARAEAGN---P--IRVGLIGA-GFMGRGIANQIINSVPGMSLVAIANRHLE :  54
Cyclobacterium_marinum_DSM_745                     : ------------------------------------MIIIDTALRKRAEEGK---P--VRVGMIGA-GFMAQGITLQIEKYTEGMELVAIANRTLS :  54
Deinococcus_maricopensis_DSM_21211                 : ------------------------------------MIIVDTALRAREAAGD---P--VRVAMIGA-GFMGRGVANQIINSVPGMRLVAVSNRTLD :  54
Desulfococcus_oleovorans_Hxd3                      : --------------------------------------------MQKQNGSD---PAKIRIGIIGI-GSMGKGLVYQAHI-TPGVRCVAVCDTDVK :  47
Frankia_sp._EuI1c                                  : ------------------------------MTTRTVRSASHVVWPTAAAAAPMLLPTDVRVGIVGT-GYTARGLAHVLSR-TPRLTVSSVLTRRPI :  64
Gloeobacter_violaceus_PCC_7421                     : ------------------------------------MILIDKALQLRAERRQ---P--VRVAMVGA-GFMGLGIAQQIVQFVPGMELVAIAGRRLE :  54
Hirschia_baltica_ATCC_49814                        : ------------------------------------MIIVDTALAKRAEEKR---P--IKVAMIGA-GFQARGILLQVAQSVPGVQIVGIAARNVD :  54
Kineococcus_radiotolerans_SRS30216                 : ------------------------------------MIIVDKALQQRQADGR---P--IRVALVGA-GFMGRGFVNQVVNSVPGMELVAIANRTVS :  54
Methanosphaerula_palustris_E1-9c                   : ------------------------------------------------MTEQ------TRIGVVGT-GFIGKGIVMSLEN-QPDLRISHVLTNSDI :  40
Nakamurella_multipartita_DSM_44233                 : ------------------------------------MILVDTALGRRQERGE---P--IRVGMIGA-GFMGRGVARQIIRYTPGMTLAAVSNRNLA :  54
Nostoc_azollae_0708                                : ------------------------------------MIIIDKALQARAALGK---P--IKVAMIGA-GFMGRGIANQIANSVPGMELVAISNRHID :  54
Nostoc_punctiforme_PCC_73102                       : ------------------------------------MIIIDRALQARAAAGN---P--IKVGMIGA-GFMGRGIANQIVNSVPGMELVAISNRKID :  54
Nostoc_sp._PCC_7120                                : ------------------------------------MIIIDNALKARAAAGN---P--VKVGMIGA-GFMGRGIANQIINSVPGMELVAISNRSID :  54
Pseudomonas_stutzeri_A1501                         : --------------------------------------------------------------------MIAHGFVRLIKQHYQDMQISRVLTRRPL :  28
Pseudomonas_stutzeri_ATCC_17588__LMG_111           : --------------------------------------------------------------------MIAHGFVRLIKQHYQDMQISRVLTRRPL :  28
Pseudoxanthomonas_spadix_BD-a59                    : -------------------------------------MVIDRLLRERQAAGM---P--IRVGLYGA-GFMAKGIVNQIVHSVPGMVLSVICNRNLD :  53
Ramlibacter_tataouinensis_TTB310                   : ------------------------------------MIIVDTALARRAAEGK---P--IRIGLVGA-GFMARGVALQVVQSVPGMRVAAIANRTLA :  54
Rhodobacter_sphaeroides_2.4.1                      : ------------------------------------MILVDKALARRAAEGR---P--IRVGMIGA-GFMGSGIALQIAKSVPGMQLVAIAARRTE :  54
Rhodobacter_sphaeroides_ATCC_17025                 : ------------------------------------MIIVDKALARRAAEGR---P--IRVGMIGA-GFMGSGIALQIANSVPGMQLVAIAARRVE :  54
Rhodobacter_sphaeroides_ATCC_17029                 : ------------------------------------MILVDKALARRAAEGR---P--IRVGMIGA-GFMGSGIALQIAKSVPGMQLVAIAARRTE :  54
Rhodobacter_sphaeroides_KD131                      : ------------------------------------MILVDKALARRAAEGR---P--IRVGMIGA-GFMGSGIALQIAKSVPGMQLVAIAARRTE :  54
Rhodothermus_marinus_DSM_4252                      : ------------------------------------------------MADS------YRIGVVGS-GFIARGFVMAMQH-VKDLRVTRVLTRTDI :  40
Rhodothermus_marinus_SG0.5JP17-172                 : --------------------------------------------MRRFVADS------YRIGVVGS-GFIARGFVMAMQH-VKDLRVTRVLTRTDI :  44
Sphingomonas_wittichii_RW1                         : ------------------------------------MILVDTALRRREEEGR---P--IRVGIVGA-GFQGAAIVRQIMTVTPGMTVAALANRNIA :  54
Streptomyces_griseus_subsp._griseus_NBRC           : ------------------------------------MMIVDTALRRRAEEGR---P--IRVGMVGA-GYMGRGLVRHISTSVAGMRVAAISNRNPA :  54
Xanthomonas_campestris_pv._campestris_st           : -------------------------------------MSVDRLLQARAAEGR---P--LRVGLYGA-GFMARGIVNQIAHAVPGMRVAVICNRSVD :  53
Xanthomonas_campestris_pv._campestris_st           : -------------------------------------MSVDRLLQARAAEGR---P--LRVGLYGA-GFMARGIVNQIAHAVPGMRVAVICNRSVD :  53
Xanthomonas_campestris_pv._campestris_st           : -------------------------------------MSVDRLLQARAAEGR---P--LRVGLYGA-GFMARGIVNQIAHAVPGMRLAVICNRSVD :  53
Achromobacter_xylosoxidans_A8                      : -------------------------------------MNLHRLLKQREADHK---P--LRVALLGA-GKFGAMFMSQAPR-TPGMRLVAVADLAPD :  52
Acidiphilium_cryptum_JF-5                          : -------------------------------------MNLSHLLAARADAGR---P--VRVLLIGA-GKFGSMFLSQVPH-TRGIEVAAIADLRPD :  52
Acidiphilium_multivorum                            : -------------------------------------MNLSHLLAARADAGR---P--VRVLLIGA-GKFGSMFLSQVPH-TRGIEVAAIADLRPD :  52
Acidovorax_ebreus_TPSY                             : -------------------------------------MNLHQKLLQRARQGR---P--IRIGLIGA-GKFGAMYLAQVPR-MPGVHLVGIADLSPA :  52
Acidovorax_sp._JS42                                : -------------------------------------MNLHQKLLQRARQGR---P--IRIGLIGA-GKFGAMYLAQVPR-MPGVHLVGIADLSPA :  52
Actinosynnema_mirum_DSM_43827                      : -------------------------------------MIHSHAVRDRQ-------P--VRIALTGANGGYGRTLLAQLRS-TPELVPALLVEPDAD :  49
Agrobacterium_sp._H13-3                            : -------------------------------------MNLYSLLSQRAAGGK---P--VRVGLIGA-GKFGSMVLAQAQR-IAGFHMVAVADLNVG :  52
Agrobacterium_tumefaciens_str._C58                 : MWPAPRRVNLRSLGIAWPSPGGLDTLASNFPSEGCQNMNLYSLLSARAAGGK---P--VRVGLIGA-GKFGSMVLAQAQR-IAGFHMVAVADLNVG :  89
Anaeromyxobacter_sp._Fw109-5                       : -------------------------------------MNLHERLLERAAAGR---P--IRVGLVGA-GKFGTMYLAQAPR-TPGVHVVAVADLRPE :  52
Arthrobacter_sp._FB24                              : -------------------------------------MNLHKLLSDREQDGR---P--IRVGLIGA-GRYGTMYLAQANN-IPGIHVVAIADINVK :  52
Azorhizobium_caulinodans_ORS_571                   : -------------------------------------MNLHALLAQRAASGK---P--VRVGLIGA-GKFGSMVLAQAQR-IEGMHVVGVADLNVG :  52
Bordetella_avium_197N                              : -------------------------------------MNLHRLLKQRETDNK---P--LRLALIGI-GKFSAMLLSQAQR-TPGIRLIAVADRDAS :  52
Bordetella_bronchiseptica_RB50                     : -------------------------------------MNLYRLLKQREADQK---P--LRVALIGA-GKFGSMFLSQVQR-TPGMRLVAVADLAPD :  52
Bordetella_parapertussis_12822                     : -------------------------------------MNLYRLLKQREADQK---P--LRVALIGA-GKFGSMFLSQVQR-TPGMRLVAVADLAPD :  52
Bordetella_petrii_DSM_12804                        : -------------------------------------MNLHRLLKQRQADHK---P--LRVALIGA-GKFGSMFLSQARR-TPGIQLVAVADLRPD :  52
Bradyrhizobium_japonicum_USDA_110                  : ----------------------------------MSPMNLFRLLQARASAGK---P--VRVALIGA-GKFGSMFLAQVPH-TPGLEVPIIVDIDRD :  55
Bradyrhizobium_sp._BTAi1                           : -------------------------------------MNLHSLLQARHASGQ---P--VRVALIGA-GKFGSMFLSQVPH-VVGLEVPVIIDLDPE :  52
Bradyrhizobium_sp._ORS278                          : -------------------------------MKPDNRMNLHALLQARAAAGK---P--VRVALIGA-GKFGSMFLSQVPN-VAGLDVPVIIDLDPQ :  58
Candidatus_Pelagibacter_ubique_HTCC1062            : -------------------------------------MFLNTKLQSRKK------P--IKVAFIGC-GKFVSMFLAQYNQ-LQKITIDTIVDINID :  49
Cupriavidus_necator_N-1                            : -------------------------------------MNFYPQLLARAEQGK---P--IRVGLIGA-GKFGSMYLAQVPR-TPGVHMVGIADLVPA :  52
Cupriavidus_taiwanensis                            : -------------------------------------MNFYPQLLARAEQGK---P--VRVGLIGA-GKFGAMYLAQVPR-TPGVHMVGIADLVPA :  52
Methylibium_petroleiphilum_PM1                     : -------------------------------------MNLHAMLAQRAADGR---P--LRVALIGA-GKFGSMFLSQVRR-TPGLHLVAVSDLSPA :  52
Methylobacterium_nodulans_ORS_2060                 : -------------------------------------MNLSTLLLQRASAGR---P--VRIGLIGA-GKFGSMVLAQAQR-IAGLHVVGVADLNVG :  52
Methylobacterium_radiotolerans_JCM_2831            : -------------------------------------MSLFAKLRRRADEGR---P--VRVGLIGA-GKFGSMYLSQAPR-TPGIHLVAVADLSPD :  52
Methylobacterium_sp._4-46                          : -------------------------------------MNLSTLLLARASAGR---P--VRVGLIGA-GKFGSMVLAQAQR-IAGLHVVGVADLNVG :  52
Mycobacterium_smegmatis_str._MC2_155               : -------------------------------------MNLYSLLQQRTEASG---P--VRVGVIGA-GKFASMFLTQAVN-SPSLHVVGVADINVS :  52
Nocardiopsis_dassonvillei_subsp._dassonv           : ----------------------------------------MQATDATTAPPP---P--VRYALTGARGGFARTLLAQTPR-MERLRPSVLCDLDTE :  50
Paracoccus_denitrificans_PD1222                    : ---------------------------------------MNYHIQFPPSSDK------VATTIIGT-GGFGRSYLAQCRK-VPLLEARVAVDVTAE :  49
Polaromonas_sp._JS666                              : -------------------------------------MNLYTKLQQRAADNQ---P--VRIGLIGA-GKFGSMYLAQIPR-TPGVHLVAIADLSPD :  52
Polymorphum_gilvum_SL003B-26A1                     : -------------------------------------MNLARMLGKRAEDDR---P--VRVGLIGG-GKFGSMYLTQARL-TTGIHVLGIADLDIR :  52
Polynucleobacter_necessarius_subsp._asym           : -------------------------------------MSLIQKLKARAANNN---P--VRVGVIGA-GKFGSMYLSQAPR-TPGIHLVAVADLSPA :  52
Pusillimonas_sp._T7-7                              : -------------------------------------MSLYAQLQERAVQNK---P--IRVGLIGA-GKFGAMYLSQIPR-TPGVHLAGIADLSPD :  52
Rhodopseudomonas_palustris_BisB5                   : -------------------------------------MNLHHLLAARADAGR---P--VRVALIGA-GKFGSMFLAQTPH-TRGLEVAAIVDLDPE :  52
Rhodospirillum_rubrum_ATCC_11170                   : ---------------------------------------MNYEHLFADLQDR---R--IHIALTGAKGGFSRTLLAQCKM-IPQITLSALCDLDVA :  51
Spirochaeta_smaragdinae_DSM_11293                  : ---------------------------------------MNYDLLFSTKGEK---K--LEVAVIGVRGFNHSLFIYGSRN--DAISIRILCGRTAQ :  50
Spirochaeta_sp._Buddy                              : -------------------------------------MLMNYSTLFKDNAKV------AQVGIIGANGGFGYSFLAQVPRMQDSLSLRVICDLDMD :  53
Streptomyces_flavogriseus_ATCC_33331               : ---------------------------------------MNLHHLFAGTGDR---T--VRYALSGAGGGFARTLLAQTPR-IGRLAPAVLCDRDVE :  51
Streptomyces_sp._SirexAA-E                         : ---------------------------------------MNLQHLFAGTGER---T--VHYALSGAGGGFARTLLAQTLR-IDRLAPAVLCDRDVD :  51
Variovorax_paradoxus_EPS                           : -------------------------------------MSLHTRLQQRAAEGR---P--IRIGLIGA-GKFGSMYLAQIPR-TPGVQLVAIADLSPD :  52
Variovorax_paradoxus_S110                          : -------------------------------------MSLYTRLQQRAAESR---P--IRIGLIGA-GKFGSMYLAQIPR-TPGVQLVAIADLSPA :  52
Xanthobacter_autotrophicus_Py2                     : -------------------------------------MNLYAKLKARAAAGR---P--VRVGVIGA-GKFGSMFLSQGPR-TPGLHIVGIADLDVN :  52
```

```
                                              100         *        120         *        140         *        160         *        180         *
Anaerococcus_prevotii_DSM_20548 : KAIKALVNAGI----SEDKIIFTDDYNEGYEVL-EKGFVCVS--TNYRLAYKLMQINAVIDCTGNPPFGAVIARKTIQYHKHMITFNVECDAVVGP : 142
Bacillus_clausii_KSM-K16 : NAEKARDFYRS-------------------QEN-RKVETVVS--SDYRAVIQSNTVEVVVDATGVPEVGANIALEALRSRKHLVLLNVEVDITIGS : 126
Bacillus_halodurans_C-125 : NVKLAYERAGV----PKDEVIETDEISKAADAI-QAGKVVAT--GDAELVTALSEVDVVVDATGIPNIGAKIAWDAILNKKHIVMLNVEADVTVGP : 142
Bacillus_pumilus_SAFR-032 : AAEKAANFYKQ--------------------HA-KPHQMVVT--NDYREVIQSDSVEVVVDATGVPEVGANISLEALNSKKHLVLLNVEVDITIGL : 125
Bartonella_bacilliformis_KC583 : RVLDAAALAYG----EKGHAIAVENTKALTEAI-EKGLIAAT--DDLDLVLKHEQIDIIVDATGYPEAGAEIGFKTLENNKHLVMMNVEADVTIGT : 148
Bartonella_clarridgeiae_73 : RVFDAAALAYG----EKGHVCEVENTTALTQTI-EKDLIAAT--DDLDLVLRHEQIDIIVDATGHPEAGAEIGFKTLRNNKHLVMMNVEADVTIGA : 148
Bartonella_grahamii_as4aup : RIFDAATIAYG----EDGHVCEVENTYALTQSI-EKNLIAAT--DDINLVLRHEQIDIIVDATGYPEAGAEIGIKALENNKHLVMMNVEADVTIGA : 148
Bartonella_henselae_str._Houston-1 : RIFDAARLAYG----EEGHVREVENASALTRTI-EKGLIAAT--NDIDLVLRHEQIDIIVDATGYPEAGAEIGFKALENNKNLVMMNVEADVTIGA : 148
Bartonella_quintana_str._Toulouse : RIFDAATLAYG----EEGHVREVENTTALTQTI-EKGLIAAT--SDIDLLLRHEQIDIIVDATGYPEVGAEIGFKALENNKHLVMMNVEADVTIGA : 148
Bartonella_tribocorum_CIP_105476 : RIFDAATLAYG----EEGHVCEVENAHALTQSI-EKGLIAAT--DDIDLVLRHEQIDIIVDATGYPEAGAEIGFKALENNKHLVMMNVEADVTIGA : 148
Brucella_abortus_bv._1_str._9-941 : NTFKAIRTAYG----DEENAREATTESAMTRAI-EAGKIAVT--DDNDLILSNPLIDVIIDATGIPEVGAETGIAAIRNGKHLVMMNVEADVTIGP : 148
Brucella_abortus_S19 : NTFKAIRTAYG----DEENAREATTESAMTRAI-EAGKIAVT--DDNDLILSNPLIDVIIDATGIPEVGAETGIAAIRNGKHLVMMNVEADVTIGP : 148
Brucella_canis_ATCC_23365 : NTFKAIRTAYG----DEENAREATTESAMTRAI-EAGKIAVT--DDNDLILSNPLIDVIIDATGIPEVGAETGIAAIRNGKHLVMMNVEADVTIGP : 148
Brucella_melitensis_ATCC_23457 : NTFKAIRTAYG----DEENARGATTESAMTRAI-EAGKIAVT--DDNDLILSNPLIDVIIDATGIPEVGAETGIAAIRNGKHLVMMNVEADVTIGP : 148
Brucella_melitensis_biovar_Abortus_2308 : NTFKAIRTAYG----DEENAREATTESAMTRAI-EAGKIAVT--DDNDLILSNPLIDVIIDATGIPEVGAETGIAAIRNGKHLVMMNVEADVTIGP : 148
Brucella_melitensis_bv._1_str._16M : NTFKAIRTAYG----DEENARGATTESAMTRAI-EAGKIAVT--DDNDLILSNPLIDVIIDATGIPEVGAETGIAAIRNGKHLVMMNVEADVTIGP : 144
Brucella_microti_CCM_4915 : NTFKAIRTAYG----DEENAREATTESAMTRAI-EAGKIAVT--DDNDLILSNPLIDVIIDATGIPEVGAETGIAAIRNGKHLVMMNVEADVTIGP : 148
Brucella_ovis_ATCC_25840 : NTFKAIRTAYG----DEENAREATTESAMTRAI-EAGKIAVT--DDNDLILSNPLIDVIIDATGIPEVGAETGIAAIRNGKHLVMMNVEADVTIGP : 148
Brucella_pinnipedialis_B2/94 : NTFKAIRTAYG----DEENAREATTESAMTRAI-EAGKIAVT--DDNDLILSNPLIDVIIDATGIPEVGAETGIAAIRNGKHLVMMNVEADVTIGP : 148
Brucella_suis_1330 : NTFKAIRTAYG----DEENAREATTESAMTRAI-EAGKIAVT--DDNDLILSNPLIDVIIDATGIPEVGAETGIAAIRNGKHLVMMNVEADVTIGP : 148
Brucella_suis_ATCC_23445 : NTFKAIRTAYG----DEENAREATTESAMTRAI-EAGKIAVT--DDNDLILSNPLIDVIIDATGIPEVGAETGIAAIRNGKHLVMMNVEADVTIGP : 148
Chelativorans_sp._BNC1 : AALQAVEIAYG----EPDRARTVDSGAAMARAI-EAGRIAVT--DDASHVIESDLIDVVVDATGVPAVGAEIGLRAMERGKHLVMMNVEADVTIGA : 148
Chloroflexus_aurantiacus_J-10-fl : QAESGYLESGV---SPEMIIHLDEDPERAAELI-DSGHRIAT--VDAAFLVQIANLDVILECTGIPEVGARVCYAAIQHSKHVVNMNVETDATIGY : 142
Chloroflexus_sp._Y-400-fl : QAESGYLESGV---SPEMIIHLDEDPERAAELI-DSGHRIAT--VDAAFLVQIANLDVILECTGIPEVGARVCYAAIQHSKHVVNMNVETDATIGY : 142
Clostridium_difficile_630 : LAHKAFTDAGI----DEEIIDNVTNEKDAEIKL-QEGKVLTC--NDFLVACKTKSIDVVIDATGGIAIGAEIALNSILNKKHIVMLNAETDCVVGP : 142
Clostridium_difficile_CD196 : LAHKAFTDAGI----DEEIIDNVTNEKDAEIKL-QEGKVLTC--NDFLVACKTKSIDVVIDATGGIAIGAEIALNSILNKKHIVMLNAETDCVVGP : 142
Clostridium_difficile_R20291 : LAHKAFTDAGI----DEEIIDNVTNEKDAEIKL-QEGKVLTC--NDFLVACKTKSIDVVIDATGGIAIGAEIALNSILNKKHIVMLNAETDCVVGP : 142
Eubacterium_limosum_KIST612 : NAKRAYLDSGY---KEGEDFVEAETVEEANKLL-AEGKFIVT--KNNEIATKCDIIDCAVDATGVPEVGAKVAMDAINAGKHICMLNVETDVCIGH : 143
Finegoldia_magna_ATCC_29328 : KCVTALRKAGV----KDVDIVRTTDLKVAENAI-KNNSFVVS--DDYTLSYKLDMIDGVIDATGNPPFGTQLAVESIEHEKHTILLNVECDAVVGP : 142
Halanaerobium_hydrogeniformans : RAKKAYQLAGI----STEDIIMSDDTAEINNYI-KEDKNVVT--SNGFAVPEVESIDVIIDATGVPDVGARIGLETINNKKHIVMMNVEADVVVGP : 142
Listeria_innocua_Clip11262 : AAKKAADAYNA-------------------TAT-KKEKILLS--NDFKEIIHSDLVEVIVDATGVPEVGAKISLETLLAKKQLVLLNVEIDITIGP : 126
Listeria_ivanovii : AAKKAADAYNA-------------------TAA-KKEKILLS--NDFKEIIHSDQVEVIVDATGVPEVGAKISLETLLAKKQLVLLNVEIDITIGP : 126
Listeria_monocytogenes : AAQKAADAYNA-------------------TAT-KKEKILLS--NDFKEIIHSDLVEVVVDATGVPEVGAKISLETLLAKKQLVLLNVEIDITIGP : 126
Listeria_monocytogenes_08-5923 : AAQKAADAYNA-------------------TAT-KKEKILLS--NDFKEIIHSDLVEVVVDATGVPEVGAKISLETLLAKKQLVLLNVEIDITIGP : 126
Listeria_monocytogenes_EGD-e : AAQKAADAYNA-------------------TAT-KKEKILLS--NDFKEIIHSDLVEVIVDATGVPEVGAKISLETLLAKKQLVLLNVEIDITIGP : 126
Listeria_monocytogenes_HCC23 : AAQKAADAYNA-------------------TAT-KKEKILLS--NDFKEIIHSDLVEVIVDATGVPEVGAKISLETLLAKKQLVLLNVEIDITIGP : 126
Listeria_monocytogenes_serotype_4b_str._ : AAQKAADAYNA-------------------TAT-KKEKILLS--NDFKEIIHSDLVEVIVDATGVPEVGAKISLETLLATKQLVLLNVEIDITIGP : 126
Listeria_monocytogenes_serotype_4b_str._ : AAQKAADAYNA-------------------TAT-KKEKILLS--NDFKEIIHSDLVEVIVDATGVPEVGAKISLETLLAKKQLVLLNVEIDITIGP : 126
Listeria_welshimeri_serovar_6b_str._SLCC : AAKKAADAYNA-------------------SAT-KKEKILLS--NDFKEIIHSDLVEVIVDATGVPEVGAKISLETLLAKKQLVLLNVEIDITIGP : 126
Mesorhizobium_ciceri_biovar_biserrulae_W : AASKAVDIAFQ----ETGHAREVSNASAMTSAM-EAGKVAVT--NDADLVINNDLIDVVIDATGVPAVGAEIGLRAMEHGKHLVMMNVEADVTIGA : 148
Mesorhizobium_loti_MAFF303099_1 : AASRAVDIAFQ----ETGHAREVSNASAMTAAM-EAGKVAVT--NDASLVINNDLIDVVIDATGVPAVGAEIGLRAMEHGKHLVMMNVEADVTIGA : 148
Mesorhizobium_loti_MAFF303099_2 : TARDAIRIAYG----DEAMAVEADAASKVTAAI-EAGKIAVT---SNEMLVTNPLIDVVIDATGKPGVAADFDLMAMEHGKHLVMMNVEADVTIGC : 141
Mesorhizobium_opportunistum_WSM2075 : AASRAVDIAFQ----ETGHAREVSNASAMTAAM-EAGKVAVT--NDANLVIDNDLIDVVIDATGVPAVGAEIGLRAMEHGKHLVMMNVEADVTIGA : 148
Ochrobactrum_anthropi_ATCC_49188_1 : NTFKAVRTAYG----DEENAREARTESAMTSAI-ESGKIAVT--DDNDLILSNPLLDVIIDATGIPEVGAQTGIKAIRNGKHLVMMNVEADVTIGP : 148
Ochrobactrum_anthropi_ATCC_49188_2 : NALKAVDIAFQ----EEGHGREVSTASDLTAAM-EAHKVAVT--DNANLILENDLIDVVIDATGVPAVGAEIGLRAMEYGKHLVMMNVEADVTIGA : 148
Pelagibacterium_halotolerans_B2 : TAMAAMDIAYGTGEGK-SHAREVESRSAMSAAI-DRGKIAVT--ADTELMVTAPEIDVIIDATGKPGVGADFDLLGMEHGKHLVMMNVEADVTIGP : 149
Roseobacter_litoralis_Och_149 : AAHKAVEIAHA----AAGFSADANSTDALNAAI-ENGKTAVA--DNADAILESGLIDVVIDATGIPAVGAEIGLRAMERGKHLVMMNVEADVTIGA : 148
Sebaldella_termitidis_ATCC_33386 : LAKKALLHAGV----SEDKIKYTKCSCEADEWI-AQGNFVVT--DDSKVVTDSKKIEVLIEATGVTEVGAKIALDTIMGKKHLVMLNAETDVVIGP : 142
Sinorhizobium_fredii_NGR234 : RARSAVDIAYQ----SDGHYQDAANSVDLNAAI-ESGKVAIT--ADSANILNSGLIDVVIDATGVPAVGAEIGLAAMEHGKHLVMMNVEADVTIGA : 148
Starkeya_novella_DSM_506 : AAIDAALMAGH----SREDIVSASTAEQIDRAI-EAGRLPIT--DDYRAMCAAGHIDVVIDATGSPNIGTIVSLEAIRNGKHVVLLSVEADITIGR : 150
Tepidanaerobacter_sp._Re1 : KAKEALRAAGV----DCKDIYVTNTVSEAEKAI-KSGKYVVT--EDFEVVAKALPVDVVIEATGVPEVGANAALKAIYNGKHIVMLNVETDVTVGP : 142
Thermosediminibacter_oceani_DSM_16646 : EARRAYTLAGV----APDDILTAKTPSEVEEAL-GRGKYAVT--EDFGAITKAIPIDVVIDATGVPEAGAHIALDSIYNGKHIVMLNVETDVTVGP : 142
Verminephrobacter_eiseniae_EF01-2 : AAVAALKAAGG----ALDSHRIVQTQGAFDQAV-HDGRIAVT--QDAQLVCRSGLIDVVVDATGKPAVGAEIGLLAMEHGKHLVMMNVEADVTIGA : 147
Anabaena_variabilis_ATCC_29413 : GAKRAYSEAGI------EDIEIVDSVGELESAI-AQGKYTIT--EDAKIICQAEGIDAIIEVTGAVEFGAHVVMEAIAHRKHVIMMNAELDGTIGP : 141
Chloroflexus_aggregans_DSM_9485 : RAIQAYTEADV----PAEAIRRVTSATALSEAL-AAGAPAVT--DDALLICAAEGIDVILEVTGAVEFGAHVALAAMQHGKHVVTMNAELDGTLGA : 143
Coraliomargarita_akajimensis_DSM_45221 : KAQHLYRVAGA------GEALVCDHSNQVDDAI-AVSKPVVC--QSPDTVIQAERIDVVVEMTGTIDLAFSWIVSAFASGKNVVSFNAELDAAVGV : 141
Coxiella_burnetii_CbuG_Q212 : SVQRAQIAYGK----------ETKLYSNADQAL-----------DDPNI-----EFDVLVDATSTIGISARYALKAIHRKAHVVLLNAHVDLFLGF : 120
Coxiella_burnetii_CbuK_Q154 : SVQRAQIAYGK----------ETKLYSNADQAL-----------DDPNI-----EFDVLVDATSTIGISARYALKAIHRKAHVVLLNAHVDLFLGF : 120
Coxiella_burnetii_Dugway_5J108-111 : SVQRAQIAYGK----------ETKLYSNADQAL-----------DDPNI-----EFDVLVDATSTIGISARYALKAIHRKAHVVLLNAHVDLFLGF : 120
Coxiella_burnetii_RSA_331 : SVQRAQIAYGK----------ETKLYSNADQAL-----------DDPNI-----EFDVLVDATSTIGISARYALKAIHRKAHVVLLNAHVDLFLGF : 120
Coxiella_burnetii_RSA_493 : SVQRAQIAYGK----------ETKLYSNADQAL-----------DDPNI-----EFDVLVDATSTIGISARYALKAIHRKAHVVLLNAHVDLFLGF : 120
Cyanothece_sp._PCC_7425 : GARQAYLEAGI------DTIEEVENSFALESAI-AAGHPAIT--ENALLICRAENIDALIEVTGTVDYGARVVMEAIAYQKPVIMMNAELDGTLGP : 141
Cyclobacterium_marinum_DSM_745 : KAKDVYSKAGQ------ENPQEVDNLEELNQVI-KEGGRAVT--NDPFLICKAEGIDAVIEVTGAVDLGARIAQECIDNGKHIIMMNAETDGTVGP : 141
Deinococcus_maricopensis_DSM_21211 : TAYRAYREAGV------EDAREVRSVEELEDAI-RAGRAAVT--EDALLICRAQGIDCLIDVTGDVEFGAHVTMDAIANGRHMVTMNAELDATVGP : 141
Desulfococcus_oleovorans_Hxd3 : RCTAVLTWLHI--------PHSIATSRAVMEDVTRRGEVAVC--EDGLWVAECADVDVVIEASSAILPAAEFALATLNSGKHLVLMNSEIDLLFGP : 133
Frankia_sp._EuI1c : DEVDG-PWAGL-----------------------------LT--HSTDRLIAQS--DLVIITTGDPVHGTEVAVQALAARLPVITMDAELQVTAGS : 126
Gloeobacter_violaceus_PCC_7421 : SAVHVYRQAGL------EEVVPVETAAQLERAI-AEGRPAVT--EDACLLCEADNVDAILEVTGSVEYAAQVALAAFTHGKHVVLMNAELDGTLGP : 141
Hirschia_baltica_ATCC_49814 : QGLFAYSQAGL-------EATTAESENDINDAI-RDGKFVVT--PNAIALAQADDIECVLEVTGSTDYAARVILATIEAGKHALHMNAEVDGTVGP : 140
Kineococcus_radiotolerans_SRS30216 : KARDAYEQAGV------TGVVEADDAAAVDRAI-SQGRPVVS--GSYEAVVDAGQVEAVVEATGNTEYGAHVVVRAIDAGKHVVLLNAEVDGTAGL : 141
Methanosphaerula_palustris_E1-9c : SRRNDYP-----------------------------GKHLLT--NSVEDLISGS--DLIVECSGEAVSATYVVDKVIKAGLPVVTMDAELQVTTGS : 103
Nakamurella_multipartita_DSM_44233 : GAQRAYREAGI------DDVDVVDSAAGVSAAV-AAGRPVVT--DDPMAVCAADGIDVICDVTGAVEFGAAITLAAFEAGKPVVTMNAELDGTVGP : 141
Nostoc_azollae_0708 : SAKRAYSEAGI------EDVQVVSSVVDLEDAL-IQGKYAIT--EDAMLLCEADGIDAIIEVTGTIEYAAHLVMRAIAHKKHIILMNAELDGTIGP : 141
Nostoc_punctiforme_PCC_73102 : AAKQAYSEAGI------EDIQVVATVSELEDAI-ANGKYAVT--EDAKLLCRAEGIEAIIEVTGAVEFGAHIVMEAIAHCKHVIMMNAELDGTIGP : 141
Nostoc_sp._PCC_7120 : GAKRAYSEAGI------EDIEIVDSVGELESAI-AQGKYTIT--EDATIICQADGIDAIIEVTGAVEFGAHVVMEAIAHRKHVIMMNAELDGTIGP : 141
Pseudomonas_stutzeri_A1501 : STMTDFPLADV-----------------------------LT--NSLDELIDNS--DLIVECSGDVFHGTAVIERAFEAGLKVVTVNAELQVTTGS : 91
Pseudomonas_stutzeri_ATCC_17588__LMG_111 : STMTDFPLADV-----------------------------LT--NSLDELIDNS--DLIVECSGDVFHGTVVIERAFEAGLKVVTVNAELQVTTGS : 91
Pseudoxanthomonas_spadix_BD-a59 : KALAAFAQAGV-------EARIVDTGRALSAAI-ASGAAAIT--DDPALLYECNELDCLVDATGAVEYGAHISLAAISYGRDMVTMNAELDGTVGP : 139
Ramlibacter_tataouinensis_TTB310 : NARRAYREAGL------EEMRTVESQAQLEQAV-AAGVPAVT--EDALLICRAEGIDAIVEVTGTIELAAAVTLEAIAHGKHVVLMNAEVDGTIGP : 141
Rhodobacter_sphaeroides_2.4.1 : QAAAAFEASRT-----GETVRHVDSQAELEAAI-VAGEPAVT--ADPSLIGCARGIEAIIEVTGSMDYALQAVEPAIATGKHVILMNAELDGTIGP : 142
Rhodobacter_sphaeroides_ATCC_17025 : QATVAFESSRT-----GESVRRVSTQAELEAAI-VAGEPAVT--DDPALVGCARGIEAIIEVTGSMDYALLAVEAAIATGKHVILMNAELDGTIGP : 142
Rhodobacter_sphaeroides_ATCC_17029 : QAAAAFEASRT-----GETVRHVDSQAELEAAI-VAGEPAVT--ADPSLIGCARGIEAIIEVTGSMDYALQAVEPAIATGKHVILMNAELDGTIGP : 142
Rhodobacter_sphaeroides_KD131 : QAAAAFEASRT-----GETVRHVDSQAELEAAI-VAGEPAVT--ADPSLVGCARGIEAIIEVTGSMDYALQAVEPAIATGKHVILMNAELDGTIGP : 142
Rhodothermus_marinus_DSM_4252 : RNRPDFPRPEL-----------------------------LT--NSLDDLIEHA--DLVVECSGDVLHATEVVSRVLEAGLPVVTMDAEFHITTGS : 103
Rhodothermus_marinus_SG0.5JP17-172 : RHRPDFPRPEL-----------------------------LT--NSLDDLIEHA--DLVVECSGDVLHATEVISRVLEAGLPVVTMDAEFHITTGS : 107
Sphingomonas_wittichii_RW1 : PAVAAFEALGI-------APRRCDSRRAVEAAI-AAGEPAVT--EDAIGLAEADGIDVVVEVTGSIEHAAAVIVAAIRAGKHVVQMNAEVDGTVGP : 140
Streptomyces_griseus_subsp._griseus_NBRC : HAERAYTEAGL-------SPTRADTADGISAAV-ALGRPVVT--EDAFALIAAEGIDCLVDVTGAVEFGAQITVAAIDRGLTVVTMNAELDCTVGP : 140
Xanthomonas_campestris_pv._campestris_st : KAIEAYRQAGQ-------QAIRVDDAAALSRQI-AAGTPAVT--DDPALLYGCDALDCLIDATGAIDYGARISLGAIANGRHMVTMNAELDGTVGP : 139
Xanthomonas_campestris_pv._campestris_st : KAIEAYRQAGQ-------QAIRVDDAAALSRQI-AAGTPAVT--DDPALLYGCDALDCLIDATGAIDYGARISLGAIANGRHMVTMNAELDGTVGP : 139
Xanthomonas_campestris_pv._campestris_st : KAIEAYRQAGQ-------QATRVDDAAALSRQI-AAGAPAVT--DDPALLYGCDALDCLIDATGAIDYGARISLGAIANGRHMVTMNAELDGTVGP : 139
Achromobacter_xylosoxidans_A8 : RARAALTRVGW--------PASALNASSTNDAL-KNGKVYFS--DDPHAVIASPDVDIVIDATGQAAAGISHVLACCEYGKHIIMVNVEADALAGP : 137
Acidiphilium_cryptum_JF-5 : GARAACRAVGW------------------DEAL--IAATRFT--DDAGAELRRDDIDVVIEATGNPPAGIAHARAAFAAGKPVVMVNVEADALAGP : 126
Acidiphilium_multivorum : GARAACRAVGW------------------DEAL--IAATRFT--DDAGAELRRDDIDVVIEATGNPPAGIAHARAAFAAGKPVVMVNVEADALAGP : 126
Acidovorax_ebreus_TPSY : NARASLARVGW--------APEQSEAASAQQAL-EQGTTWIT--EDWQALVRHPQIELIVECTGNPLAAVDHILDAFAHGKHVVNVTVEADAFCGP : 137
Acidovorax_sp._JS42 : NARASLARVGW--------APEQSEAASAQQAL-EQGTTWIT--EDWQALVRHPQIELIVECTGNPLAAVDHILDAFAHGKHVVNVTVEADAFCGP : 137
Actinosynnema_mirum_DSM_43827 : GMRRTLAELGL-----------------------DAGAITDG--SDVDW----SGVDVLIEASGKVAAGTGYATAAIEHGVHVVMVSKEVDTVAGV : 116
Agrobacterium_sp._H13-3 : KARESFDRVGW--------PKERYDATSCADAL-KSGKTYVT--DDVNELFACGEIECVIEATGHPLAGARHALGAIDHNKHVVMVNVEADVMVGP : 137
Agrobacterium_tumefaciens_str._C58 : KARESFDRVGW--------PKERYDATSCADAL-KSGKTYVT--DDVNELFACGEIECIIEATGHPLAGARHALGAIDHNKHVVMVNVEADVMVGP : 174
Anaeromyxobacter_sp._Fw109-5 : RARDALARAGW--------PAERAGARGAEEAL-ARGTTWIT--DDAERLIASAGVELVIDATGSPSAGIRHVLSCCAHGKDVVMVNVEADALAGP : 137
Arthrobacter_sp._FB24 : RAEGAFELVGW---------PKNQIAPDIATAL-RDRSTTIV--ANADELFDVD-IDVIVEATGNPIVGVKHALRAIETKKHIIMVTVEADALAGP : 135
Azorhizobium_caulinodans_ORS_571 : RARASLARVGW--------PEERYGATSLADAF-KSGKTCVL--DDANALAAFDGVECIIEATGHPIAGVRHALAAIDAGKHVVMVNVEADVLCGP : 137
Bordetella_avium_197N : RARSALTRCGW--------PAQTLGASTIHEAL-KHGKVFCC--DDPLQVIADPDVEIVIDASTLAAEGIAHTLWCCEYRKDIIMVNLAADALAGP : 137
Bordetella_bronchiseptica_RB50 : RARAALTRVGW--------PAAALNADTIHHAV-KTGKVYFS--DDVRSVIVDPEVEIVIDATGQAAAGIEHVLTCCEYGKHIIMVNVEADALAGP : 137
Bordetella_parapertussis_12822 : RARAALTRVGW--------PAAALNADTIHHAV-KTGKVYFS--DDVRSVSVDPEVEIVIDATGQAAAGIEHVLTCCEYGKHIIMVNVEADALAGP : 137
Bordetella_petrii_DSM_12804 : RARAALTRVGW--------PADALRADSAPDAL-RSGRVYFS--DDAAAVIASPEIEIVIDATGQAAAGIAHVLACCEHGKHIIMVNVEADALAGP : 137
Bradyrhizobium_japonicum_USDA_110 : RAREACRTVGW-------------------DAA-RIAATVFT--DDGARAIAGGAMDVVVEATGNPAVGIRHARAAIAAGKHIVMVNVEADVLAGP : 129
Bradyrhizobium_sp._BTAi1 : RARDACRTIGW----SAELIERTAFSVDGAKAL-------------------ADNIDVVVEATGSPAAGIRHARAAIRAGKHIVMVNVEADVLAGP : 125
Bradyrhizobium_sp._ORS278 : RAREACRTVGW----SAELIERTTFSADSAKAL-------------------ADSIDVVVEATGSPAAGIRHARAAIRAGKHIVMVNVEADVLAGP : 131
Candidatus_Pelagibacter_ubique_HTCC1062 : NAKKNCLKSGL----TTETVNKINFVTTLDEAI-------------------DRDVEVFIEATGNPIIGTVHAVKIIKNKKHIILVNVEADVTCGK : 122
Cupriavidus_necator_N-1 : NAHASLERVGW--------EAGRAQAASLDDAV-RMGSTHVG--DDWEALVAHPGVDIIIECTGNPIAAVDHCLAAFKHGKHVINVTVEADAFCGP : 137
Cupriavidus_taiwanensis : NAHASLERVGW--------EAGRARAASLDDAL-RTGATHVG--EDWQSLVAHPAIDIIIECTGNPIAAVDHCLAAFGHGKHVINVTVEADAFCGA : 137
Methylibium_petroleiphilum_PM1 : RAKAALLSVGW--------DEDRLGAKTLDDAH-RSGSTCLT--DDTPAVIASDAVEVVIDATGSPAAGIAHVLACCRHGKHIVMVNVEADALAGP : 137
Methylobacterium_nodulans_ORS_2060 : RARDSLARVGW--------PEERYAAPTLGDAV-RTGRTCVL--DDAGALAACDEIECIIEATGHPIAGIRHALQAIEAGKHVVMVNVEADVLCGP : 137
Methylobacterium_radiotolerans_JCM_2831 : RAREALRRVGW--------DDARFAARALEAAA-RGGTTLVT--EDAEAMIASPFVDIVIDATGSPAAGIRHALQAFAAAKHIVMVNVEADVLAGP : 137
Methylobacterium_sp._4-46 : RARDSLARVGW--------PEERYAAPTLGDAV-RSGRTCVL--DDAAALSACDEIECIIEATGHPIAGIRHALQAIDAGKHVVMVNVEADVLCGP : 137
Mycobacterium_smegmatis_str._MC2_155 : KAKAALHRTGW--------PADRYAAGSLDEAL-RTGATAVT--DNADALLAHPGIEVILEITGNPLVGTYHAVTAIDNGKHVVMVNVEADCMVGP : 137
Nocardiopsis_dassonvillei_subsp._dassonv : GTIALCAELGY----PADALTVCSSADEVAAL--ADGRIAVI--ADARLLEHAD-YDVLVEATGSPEAGTRAARTAIAAGRHVVMVSKEVDSVSGI : 137
Paracoccus_denitrificans_PD1222 : QAARTMIEVGY----PEARIRKCDTAEEAAAAW-AAGDFIAA--GDFAHVAALP-VHVVIEATGHPEAGARHCRLAVEAGHHVALVSKEVDSVVGP : 137
Polaromonas_sp._JS666 : AARTNLTRVGW--------KPEMSQAESAQTAI-KTGATWIT--DDWQAVVTHPQIDIIVECTGNPVAAVEHCLQAFAHGKHVVNVTVEADAFCGP : 137
Polymorphum_gilvum_SL003B-26A1 : RLRGTLRRAGW--------PDEQTDTASFSDAL-GSGRTFLT--DSAEALIAADGLDVLIDATGDPAAGIRHCLAAIRNGRHVVMVNVEADALAGP : 137
Polynucleobacter_necessarius_subsp._asym : RAKESLARVGW--------DTPRYSATSMQDAA-KSGATFVT--DDAEKMIASEYIDIVIDATGSPAAGIRHALLCFDHRKHIIMVNVEADVLAGP : 137
Pusillimonas_sp._T7-7 : AARSNLARVGW--------KSEQYGATSADEAL-RTGSTWIT--DDWKALVGLPDIDVIVECTGNPIAAVEHCLAAFEHGKHVVNVTVEADAFCGP : 137
Rhodopseudomonas_palustris_BisB5 : RARDACRHVGW-----------------------DEGRIAATRFDSDPAAANAAGIEVVVEATGNPAAGIRHARAAIAAGQHVVMVNVEADVLAGP : 125
Rhodospirillum_rubrum_ATCC_11170 : GTAALLESLGF----PADSHAICATPIEVTAAA-ASGRIALV--ADYRLLDGLA-LDIVVEATGNPEISMQIALAALRRQVHVAMVSKETDSVVGP : 139
Spirochaeta_smaragdinae_DSM_11293 : KCIDAYLSVGI----KRENIALCSSTAEGLSAF-GKGMYLVF--TDIDTAMSMP-CDLVVEGTGNPEASARYALSAIEHHKHIVMVTKESDSVVGS : 138
Spirochaeta_sp._Buddy : VSRKLLESLKY----DSSRFHPCRNQRDIEQAL-KTDDPIII--LEDGTLLPFCKIDVLVEATGSPEVSSVMAQACLKQGIHVCMVSKEADCVSGP : 142
Streptomyces_flavogriseus_ATCC_33331 : RLRGMLVELGY----PADRLAVCADAAEVARAA-EQGHVALV--ADGALLPAAH-WDILVEATGSPADGYAMAREALTGGRHVAMVSKEVDSVAGL : 139
Streptomyces_sp._SirexAA-E : RLRGMLVELGY----EDGRLRVCADADEVAHAV-EEGSVALV--ADGALLTAAP-WDILVEATGSPADGYAMARAALTGGRHVAMVSKEVDSVAGL : 139
Variovorax_paradoxus_EPS : AARVNLARVGW--------KPERTAAGSVQEAI-KTGNTWIT--DNWQAVTREPSIDIVVECTGHPIAAVDHCLDAFAHGKHVVNVTVEADAFCGP : 137
Variovorax_paradoxus_S110 : AARANLERVGW--------NPERAAAGSVQEAL-KTGSTWIT--DDWQAVTREPAIDIVVECTGNPVAAVEHCLDAFAHGKHVVNVTVEADAFCGP : 137
Xanthobacter_autotrophicus_Py2 : RARASCARVDW--------PAERYAARSFAEAM-ASGSTFIT--DDAEALIATGGMDVIVEATGNPGAGIRHALLCCKHGRHIVMVNVEADALAGP : 137
```

```
                                                    200         *        220         *        240         *        260         *        280
Anaerococcus_prevotii_DSM_20548               : VLHDMAKKAGVVYTGILGDEPGAIIDLVEYAYGMGLEVLVAA--KG-KNNPLDRDATPESL-------AEKAK-EK------------GLSAKMLT : 215
Bacillus_clausii_KSM-K16                      : YMHSMFDAAGLVYSGSAGDEPAAIVELYEFAKTMGLEVVVAG--KG-KNNPLKPTANPDTC-------AEEAK-QK------------HMSAHMLA : 199
Bacillus_halodurans_C-125                     : LLKQMADACGVVYTGSAGDEPGAVMELYDFADALGFEVVALG--KG-KNNPLNVEANPQTA-------AEEAK-RK------------GASPKMLA : 215
Bacillus_pumilus_SAFR-032                     : VMHKLFTNAGLVYSGSAGDEPAATLELYEFAKTMGLEVLVAG--KG-KNNPFFPEANPDTC-------KAEAD-SK------------HMSAHMLA : 198
Bartonella_bacilliformis_KC583                : YLKHEADKRGLIYTLGAGDEPSSCMELIEFVSALGHQIIAAG--KG-KNNPLIFDAIPDHY-------EEEAS-RR------------NMNVRMLV : 221
Bartonella_clarridgeiae_73                    : YLKNEADKRGLIYTLGAGDEPTSCMELIEFVSALGHKIVAVG--KG-KNNPLIFDATPDAY-------EEEAR-QR------------NMNVRMLV : 221
Bartonella_grahamii_as4aup                    : YLKHEAEKRGLIYTLGAGDEPTSCMELIEFVSALGHKIVAAG--KG-KNNPLRFDATPDAY-------EEEAF-RR------------NMNVRMLV : 221
Bartonella_henselae_str._Houston-1            : YLKHEAEKQGLIYTLGAGDEPTSCMELIEFVSALGHKIVAAG--KG-KNNPLIFDATPDTY-------EEEAL-RR------------NMNVRMLV : 221
Bartonella_quintana_str._Toulouse             : YLKHEAEKRGLIYTLGAGDEPTSCMELIEFVSALGHKIVAAG--KG-KNNPLIFDATPDVY-------EEEAS-RR------------NMNVRMLV : 221
Bartonella_tribocorum_CIP_105476              : YLKHEAEKRGLIYTLGAGDEPTSCMELIEFVSALGHKIVAAG--KG-KNNPLLFDATPDAY-------EEEAL-RR------------NMNVRMLV : 221
Brucella_abortus_bv._1_str._9-941             : YLKAQADKQGVIYSLGAGDEPSSCMELIEFVSALGYEVVSAG--KG-KNNPLNFDATPDDY-------RQEAD-RR------------NMNVRLLV : 221
Brucella_abortus_S19                          : YLKAQADKQGVIYSLGAGDEPSSCMELIEFVSALGYEVVSAG--KG-KNNPLNFDATPDDY-------RQEAD-RR------------NMNVRLLV : 221
Brucella_canis_ATCC_23365                     : YLKAQADKQGVIYSLGAGDEPSSCMELIEFVSALGYEVVSAG--KG-KNNPLNFDATPDDY-------RQEAD-RR------------NMNVRLLV : 221
Brucella_melitensis_ATCC_23457                : YLKAQADKQGVIYSLGAGDEPSSCMELIEFVSALGYEVVSAG--KG-KNNPLNFDATPDDY-------RQEAD-RR------------NMNVRLLV : 221
Brucella_melitensis_biovar_Abortus_2308       : YLKAQADKQGVIYSLGAGDEPSSCMELIEFVSALGYEVVSAG--KG-KNNPLNFDATPDDY-------RQEAD-RR------------NMNVRLLV : 221
Brucella_melitensis_bv._1_str._16M            : YLKAQADKQGVIYSLGAGDEPSSCMELIEFVSALGYEVVSAG--KG-KNNPLNFDATPDDY-------RQEAD-RR------------NMNVRLLV : 217
Brucella_microti_CCM_4915                     : YLKAQADKQGVIYSLGAGDEPSSCMELIEFVSALGYEVVSAG--KG-KNNPLNFDATPDDY-------RQEAD-RR------------NMNVRLLV : 221
Brucella_ovis_ATCC_25840                      : YLKAQADKQGVIYSLGAGDEPSSCMELIEFVSALGYEVVSAG--KG-KNNPLNFDATPDDY-------RQEAD-RR------------NMNVRLLV : 221
Brucella_pinnipedialis_B2/94                  : YLKAQADKQGVIYSLGAGDEPSSCMELIEFVSALGYEVVSAG--KG-KNNPLNFDATPDDY-------RQEAD-RR------------NMNVRLLV : 221
Brucella_suis_1330                            : YLKAQADKQGVIYSLGAGDEPSSCMELIEFVSALGYEVVSAG--KG-KNNPLNFDATPDDY-------RQEAD-RR------------NMNVRLLV : 221
Brucella_suis_ATCC_23445                      : YLKAQADKQGVIYSLGAGDEPSSCMELIEFVSALGYEVVSAG--KG-KNNPLNFDATPDDY-------RQEAD-RR------------NMNVRLLV : 221
Chelativorans_sp._BNC1                        : YLKNEADRLGVTYSLGAGDEPSACMELIEFVSAMGHTIVAAG--KG-KNNPLNIDAVPDDY-------AAEAE-RR------------NMNVRMLV : 221
Chloroflexus_aurantiacus_J-10-fl              : RLAKLAAERGVIYTLAAGDEPGAIKELFDFADALGFEVAYIG--KG-KNNPLDRTATPDRL-------RLQAQ-KQ------------GMNPKMLT : 215
Chloroflexus_sp._Y-400-fl                     : RLAKLAAERGVIYTLAAGDEPGAIKELFDFADALGFEVAYIG--KG-KNNPLDRTATPDRL-------RLQAQ-KQ------------GMNPKMLT : 215
Clostridium_difficile_630                     : ILKKLADDAGVIFTGSAGDEPGAVMELFDFADAMGFEVRVIG--KG-KNNKLDLDCNPDTV-------REEAE-RK------------GASPHMIA : 215
Clostridium_difficile_CD196                   : ILKKLADDAGVIFTGSAGDEPGAVMELFDFADAMGFEVRVIG--KG-KNNKLDLDCNPDTV-------REEAE-RK------------GASPHMIA : 215
Clostridium_difficile_R20291                  : ILKKLADDAGVIFTGSAGDEPGAVMELFDFADAMGFEVRVIG--KG-KNNKLDLDCNPDTV-------REEAE-RK------------GASPHMIA : 215
Eubacterium_limosum_KIST612                   : LLYKLANNAGVVYTGSAGDEPGAVVEMYDYAKGLGFDVKVVG--KG-KNNPLALECTPETV-------AEIAK-EK------------GASPKMIC : 216
Finegoldia_magna_ATCC_29328                   : ILNEMAKKKGVVYTGSAGDEPGAIIQLSDFALGLGFKLLAVG--KG-KNNPLDNYTNEDIL-------REEAL-SK------------GLVPKMLT : 215
Halanaerobium_hydrogeniformans                : ILKKMADNAGVVYTGAAGDEPGAIMEIYNFAKSLGYKVVAAG--KG-KNNPLDRKANPERV-------AERAK-QK------------GTAPHMMA : 215
Listeria_innocua_Clip11262                    : LMKKLYDSAGLVYTGSDGDEPAAITELYEFSKSMGMEVLVAG--KG-KNNKLKISANPDSC-------QVEAD-GK------------NMASHMLA : 199
Listeria_ivanovii                             : LMKKLYDSAGLVYTGSDGDEPAAITELYEFSKSMGMEVLVAG--KG-KNNKLKISANPDSC-------QVEAD-EK------------NMASHMLA : 199
Listeria_monocytogenes                        : LMKKLYDSAGLVYTGSDGDEPAAITELYEFSKSMGMEVLVAG--KG-KNNKLKISANPDSC-------QVEAD-GK------------NMASHMLA : 199
Listeria_monocytogenes_08-5923                : LMKKLYDSAGLVYTGSDGDEPAAITELYEFSKSMGMEVLVAG--KG-KNNKLKISANPDSC-------QVEAD-GK------------NMASHMLA : 199
Listeria_monocytogenes_EGD-e                  : LMKKLYDSAGLVYTGSDGDEPAAITELYEFSKSMGMEVLVAG--KG-KNNKLKISANPDSC-------QVEAD-GK------------NMASHMLA : 199
Listeria_monocytogenes_HCC23                  : LMKKLYDSAGLVYTGSDGDEPAAITELYEFSKSMGMEVLVAG--KG-KNNKLKISANPDSC-------QAEAD-GK------------NMASHMLA : 199
Listeria_monocytogenes_serotype_4b_str._      : LMKKLYDSAGLVYTGSDGDEPAAITELYEFSKSMGMEVLVAG--KG-KNNKLKISANPDSC-------QAEAD-GK------------NMASHMLA : 199
Listeria_monocytogenes_serotype_4b_str._      : LMKKLYDSAGLVYTGSDGDEPAAITELYEFSKSMGMEVLVAG--KG-KNNKLKISANPDSC-------QAEAD-GK------------NMASHMLA : 199
Listeria_welshimeri_serovar_6b_str._SLCC      : LMKKLYDSAGLVYTGSDGDEPAAITELYEFSKSMGMEVLVAG--KG-KNNKLKISANPDSC-------QAEAD-GK------------NMASHMLA : 199
Mesorhizobium_ciceri_biovar_biserrulae_W      : YLKSEADRLGVTYSLGAGDEPSSCMELIEFVSAMGHPIVAAG--KG-KNNPLNIDAIPPDY-------EEEAK-RR------------HMNVRMLV : 221
Mesorhizobium_loti_MAFF303099_1               : YLKSEADRLGVTYSLGAGDEPSSCMELIEFVSAMGHPIVAAG--KG-KNNPLNIDATPPAH-------EEEAE-RR------------HMNVRMLV : 221
Mesorhizobium_loti_MAFF303099_2               : YLKQQADRLGVVYSVGAGDEPSSCMELIEFASALGLTIVSAG--KG-KNNPLNHDAMPDDY-------REEAI-RR------------NMNPRMLV : 214
Mesorhizobium_opportunistum_WSM2075           : YLKSEADRLGVTYSLGAGDEPSSCMELIEFVSAMGHPIVAAG--KG-KNNPLNIDATPPAY-------EEEAR-RR------------HMNVRMLV : 221
Ochrobactrum_anthropi_ATCC_49188_1            : YLKAEADKHGVIYSLGAGDEPSSCMELIEFVSAMGHKVVAAG--KG-KNNPLNFDAIPDDY-------QEEAD-RR------------NMNVRLLV : 221
Ochrobactrum_anthropi_ATCC_49188_2            : YLKAEAERLGVTYSLGAGDEPSSCMELIEFVSAMGHPIVAAG--KG-KNNPLNIDAVPDQY-------LEEAT-RR------------NMNVRMLV : 221
Pelagibacterium_halotolerans_B2               : YLKHEADRLGVVYSVGAGDEPSSCMELIEFCSALGLEIVAAG--KG-KNNPLNHDAVPDDY-------REEAT-RR------------NMNPRMLV : 222
Roseobacter_litoralis_Och_149                 : YLRREANRLGVVYSLGAGDEPSSCMELIEFVSAMGHKIVCAG--KG-KNNPLNFDAIPEAY-------MEEAA-RR------------HMNPRLLV : 221
Sebaldella_termitidis_ATCC_33386              : LLKKLADNAGVVYTGAAGDEPAATKELYDFAKAAGFDVRVIG--KG-KNNKIDKDCNPDTV-------AEEAE-RR------------GVSAHMLA : 215
Sinorhizobium_fredii_NGR234                   : YLKSEAERLGVTYSLGAGDEPSSCIELIEFVSSMGHPIVAAG--KG-KNNPLNVAAVPDDY-------AEEAA-RR------------NMNVRMLV : 221
Starkeya_novella_DSM_506                      : FLKEEARKAGVVLTGAAGDEPAATIELIGFAQSLGFDIVCAG--KA-KNNAFKPDAVPDEF-------AAEAK-AR------------NMNPRMLV : 223
Tepidanaerobacter_sp._Re1                     : VLKQMADSAGVVYTVSAGDEPGAIKELYDFADAVGFNVVAVG--KG-KNNPLNREANPDTM-------RQEAD-AK------------GINPRMLT : 215
Thermosediminibacter_oceani_DSM_16646         : ILKKMADSAGVVYTVSAGDEPGAIKELYDFADAMGFEILVAG--KG-KNNPLDLEANPDSV-------MEEAM-KK------------NMNPKMLA : 215
Verminephrobacter_eiseniae_EF01-2             : YLKREADRLGVVYSLGAGDEPSATIELIGFVSALGYPVIAAG--KG-KNNPLNFDATPDDY-------AAQAR-RK------------HLNPRLLV : 220
Anabaena_variabilis_ATCC_29413                : ILKVYADKAGVILSACDGDQPGVQMNLYRFVQSIGLTPLLCGNIKG----LQDPYRNPTTQ-------EGFAK-RW------------GQKAHMVT : 213
Chloroflexus_aggregans_DSM_9485               : ILQVYARRYGVIFTLSDGDQPGVTMNLYRFVRGLGVKPVLCGNIKG----LHDPYRNPTTQ-------ANFAR-QW------------GQNPYMVT : 215
Coraliomargarita_akajimensis_DSM_45221        : YLQQQARARGVRYTLGDGDQPGVTLNLYRYVSAMGMQPLVCGNNKG----MLDHYRTPETQ-------KGFAE-QT------------GMSVDMVT : 213
Coxiella_burnetii_CbuG_Q212                   : YLQHEAAKHGVIVTSDAGDQHGVLARMIEELQLWGFQITQAGNIKG----FLDRHSTADSK-------REIAK-KL------------NLKVVPCV : 192
Coxiella_burnetii_CbuK_Q154                   : YLQHEAAKHGVIVTSDAGDQHGVLARMIEEIQLWGFQITQAGNIKG----FLDRHSTADSK-------REIAK-KL------------NLKVVPCV : 192
Coxiella_burnetii_Dugway_5J108-111            : YLQHEAAKHGVIVTSDAGDQHGVLARMIEEIQLWGFQITQAGNIKG----FLDRHSTADSK-------REIAK-KL------------NLKVVPCV : 192
Coxiella_burnetii_RSA_331                     : YLQHEAAKHGVIVTSDAGDQHGVLARMIEEIQLWGFQITQAGNIKG----FLDCHSTADSK-------REIAK-KL------------NLKVVPCV : 192
Coxiella_burnetii_RSA_493                     : YLQHEAAKHGVIVTSDAGDQHGVLARMIEEIQLWGFQITQAGNIKG----FLDRHSTADSK-------REIAK-KL------------NLKVVPCV : 192
Cyanothece_sp._PCC_7425                       : ILKVYADRAGVILTFSDGDQPGVQMNLYRFVKSIGLTPLLCGNVKG----LQDPYRNPTTQ-------EGFAR-RW------------GQKPHMVT : 213
Cyclobacterium_marinum_DSM_745                : ILKVHADNKGVVFTNADGDQPGVTLNLFRFVKQLGLKPVLCGNIKG----LHDPYRNPTTQ-------EGFAK-KW------------KQKPAMVT : 213
Deinococcus_maricopensis_DSM_21211            : MLKVLADRAGVIFTAADGDQPGVQMNLYRFVRSIGLTPVLCGNIKG----LQDPYRTPTTQ-------QAFAE-RW------------GQNPYMVT : 213
Desulfococcus_oleovorans_Hxd3                 : LLADIARKNGVVCTSCDGDQYGVLKHQIDDLALWGLDLVMAGNIKG----FLDRSANPTSI-------VPEAD-IR------------NLDYRMCT : 205
Frankia_sp._EuI1c                             : YLASLG-----VVVEAQGDQPGSLALLAHEVRQMGLTPLVYGNIKG----FLNHHPKPAEM-------AYWAE-RQ------------GISLTQVT : 193
Gloeobacter_violaceus_PCC_7421                : ILKRYADRAGVVYTACDGDQPGVQMNLWRFVRSMGLTPLVCGNVKG----LHDPYRNPTTQ-------EAFAR-RW------------GQKPHMVT : 213
Hirschia_baltica_ATCC_49814                   : ILKVKADKAGVIYSFSDGDQPGVEANLLRVVKAMGVKPVLAGNIKG----LHDPYRNPTTQ-------AGFAK-QY------------KQKPAMVA : 212
Kineococcus_radiotolerans_SRS30216            : ALRTRAEKAGVIYTGCDGDQPGVQMNLIRFVRSIGVTPLVSGNIKG----LQDEYRNPTTQ-------EGFAK-QW------------GQDPYMVT : 213
Methanosphaerula_palustris_E1-9c              : YFAKKG-----FITEAEGDQPGCLAALDEEARQMGFSPQVYGNIKG----YLNYTPTIEDM-------RFWSQ-KQ------------GISLDKTT : 170
Nakamurella_multipartita_DSM_44233            : LLAHRAAAAGLVFSGVDGDQPGVEINLFRFVTGRGLTPLVLGNIKG----LQDRFRNPTTQ-------AGFAA-QW------------GQDPHMVT : 213
Nostoc_azollae_0708                           : ILKLHAERAGVILTACDGDQPGVEMNLYRFVKSIGLTPLLCGNIKG----LQDPYRNPTTQ-------ASFAQ-HW------------GQNPAMVT : 213
Nostoc_punctiforme_PCC_73102                  : ILKVYADKAGVILSACDGDQPGVQMNLYRFVKSIGLTPLLCGNIKG----LQDPYRNPTTQ-------EGFAK-RW------------GQKPHMVA : 213
Nostoc_sp._PCC_7120                           : ILKVYADKAGVILSACDGDQPGVQMNLYRFVQSIGLTPLLCGNIKG----LQDPYRNPTTQ-------EGFAK-RW------------GQKAHMVT : 213
Pseudomonas_stutzeri_A1501                    : YLAGKG-----FLTEAEGDQPGSLAALREDALQMGFQPLVYGNMKG----YLNHDPSPEDM-------AYWAN-RQ------------GISVDQTT : 158
Pseudomonas_stutzeri_ATCC_17588__LMG_111      : YLAGKG-----FLTEAEGDQPGSLAALREDALQMGFQPLVYGNMKG----YLNHDPSPEDM-------AYWAN-RQ------------GISVDQTT : 158
Pseudoxanthomonas_spadix_BD-a59               : LLKKKADAAGVIFTACDGDQPGVQMNLFRFVRSIGLTPLMCGNIKG----LQDPYRTPTTQ-------AGFAA-KW------------GQDPYMVT : 211
Ramlibacter_tataouinensis_TTB310              : ILKTYADRAGVVLTSADGDQPGVMMNLWRFVRGIGVKPVLCGNIKG----LHDPYRNPTTQ-------AGFAR-QW------------GQNPQMVT : 213
Rhodobacter_sphaeroides_2.4.1                 : LLKKRADAAGVVLTNCDGDQPGVQMNLIRFVKGIGVKPVLSGNIKA----LQDEYRTPETQ-------RGFAE-KW------------GQNVHMVT : 214
Rhodobacter_sphaeroides_ATCC_17025            : LLKKRADAAGVVITNCDGDQPGVQMNLIRFVKGIGVKPVLSGNIKA----LQDEYRTPETQ-------RGFAE-KW------------GQNVHMVT : 214
Rhodobacter_sphaeroides_ATCC_17029            : LLKKRADAAGVVLTNCDGDQPGVQMNLIRFVKGIGVKPVLSGNIKA----LQDEYRTPETQ-------RGFAE-KW------------GQNVHMVT : 214
Rhodobacter_sphaeroides_KD131                 : LLKKRADAAGVVLTNCDGDQPGVQMNLIRFVKGIGVKPVLSGNIKA----LQDEYRTPETQ-------RGFAE-KW------------GQNVHMVT : 214
Rhodothermus_marinus_DSM_4252                 : YFVDRG-----LLTEAEGDQPGCLAALREDILQMGFRPLVYGNIKR----YLNHNPTPEEM-------HFWAR-KQ------------GITLPQVT : 170
Rhodothermus_marinus_SG0.5JP17-172            : YFVDRG-----LLTEAEGDQPGCLAALREDILQMGFRPLVYGNIKR----YLNHNPTPEEM-------HFWAR-KQ------------GITLPQVT : 174
Sphingomonas_wittichii_RW1                    : ILKRKADAAGLVYSFSDGDQPGVQMNLFRFVAGLGVKPVLCGNIKG----LHDPKRNPTTQ-------QAFAE-KW------------GQKPAMVA : 212
Streptomyces_griseus_subsp._griseus_NBRC      : LLAHRARSAGVILTGADGDQPAVQANLLRFVRGLGVTPLVAGNVKG----FQDEYRTPTTQ-------QPFAE-RW------------GQNVHMIT : 212
Xanthomonas_campestris_pv._campestris_st      : LLKRKADAAGVIFSACDGDQPGVQMNLYRFVRSIGLTPLLCGNIKG----LQDPYRTPTTQ-------AGFAA-KW------------GQDPHMVT : 211
Xanthomonas_campestris_pv._campestris_st      : LLKRKADAAGVIFSACDGDQPGVQMNLYRFVRSIGLTPLLCGNIKG----LQDPYRTPTTQ-------AGFAA-KW------------GQDPHMVT : 211
Xanthomonas_campestris_pv._campestris_st      : LLKRKADAAGVIFSACDGDQPGVQMNLYRFVRSIGLTPLLCGNIKG----LQDPYRTPTTQ-------AGFAA-KW------------GQDPHMVT : 211
Achromobacter_xylosoxidans_A8                 : LLARRAREAGIVYSLAYGDQPALICEMVDWARASGFEVMAAG--KGTKYLPEFHTSTPDTVWPYYGFTAEMVA-AG------------DFNAQMFN : 218
Acidiphilium_cryptum_JF-5                     : LLAAEADAAGVTYSLAYGDQPALVAEMVDWARACGFSVVAAG--KGTKYLPLYHGVTPDTVWQHYGLTAEQAR-EG------------GMNPQMFN : 207
Acidiphilium_multivorum                       : LLAAEADAAGVTYSLAYGDQPALVAEMVDWARACGFSVVAAG--KGTKYLPLYHGVTPDTVWQHYGLTAEQAR-EG------------GMNPQMFN : 207
Acidovorax_ebreus_TPSY                        : LLARKALAAGVVYSLAYGDQPAMICELVDWARTCGFPVVAAG--RGHKWLPHFCESTPETVWGYYGLTPEQAQ-RG------------GLNPKMFN : 218
Acidovorax_sp._JS42                           : LLARKAQAAGVVYSLAYGDQPAMICELVDWARTCGFPVVAAG--RGHKWLPHFCESTPETVWGYYGLTPEQAQ-RG------------GLNPKMFN : 218
Actinosynnema_mirum_DSM_43827                 : ALAARAAEAGLRYLPGDGDQPANLLRLLDWVTATGLEVVAIG--KSGEYDLVLDPATGVVTQAGATVHAPGLVDLL--DLGADPRETLARRAELVA : 208
Agrobacterium_sp._H13-3                       : ILAEKARAKGLIYSMAYGDQPALICELVDWARATGFEVVSAG--KGMNFEPRYRYSTPDTVWSYFGWTDEEVA-KG------------DFNPKMYN : 218
Agrobacterium_tumefaciens_str._C58            : ILAEKARAKGLIYSMAYGDQPALICELVDWARATGFEVVSAG--KGMNFEPRYRYSTPDTVWSYFGWTDEEVA-KG------------DFNPKMYN : 255
Anaeromyxobacter_sp._Fw109-5                  : LLAERARQAGIVYSLAYGDQPALVSELVDWARAAGFAVVAAG--KGTRYLPRYHASTPDTVWADYGIPPEEAR-AS------------GLNAQMFN : 218
Arthrobacter_sp._FB24                         : ALARRAEAAGVVYSMAYGDQPALIMELVDWARTSGFDVVCAG--KGAKFLEHYHEMNPDNVWENWEFSKELTD-SG------------QLNPNMHT : 216
Azorhizobium_caulinodans_ORS_571              : LLAERARQKGVVYSMAYGDQPAIICELVDWARSCGFELTSAG--KGMNFEPRYRYSTPDTVWGFFGWSEEEVA-KG------------DFNPKMYN : 218
Bordetella_avium_197N                         : LLARRAREAGIVYSLAYGDQPALICEMVDWARACGFEIMAAG--KGTKYLPEFHTSTPDTVWPYYGFTDEMVA-AG------------DFDARLFN : 218
Bordetella_bronchiseptica_RB50                : LLARRAREAGIVYSLAYGDQPALICEMVDWARASGFEVVAAG--KGTKYLPEFHTSTPDTVWPYYGFTDEMVA-AG------------DFNAQMFN : 218
Bordetella_parapertussis_12822                : LLARRAREAGIVYSLAYGDQPALICEMVDWARASGFEVVAAG--KGTKYLPEFHTSTPDTVWPYYGFTDEMVA-AG------------DFNAQMFN : 218
Bordetella_petrii_DSM_12804                   : LLARRAREAGIVYSLAYGDQPALICEMVDWARASGFEVIAAG--KGTKYLPEFHTSTPDTVWPYYGFTDEMVA-AG------------DFNAQMFN : 218
Bradyrhizobium_japonicum_USDA_110             : LLAEEARKAGVVYSLAYGDQPALTAEMVDWARATGFRVVAAG--KGTKYLPAYHDVTPDGVWQHYGLTAGEAQ-SA------------GMNPQMFN : 210
Bradyrhizobium_sp._BTAi1                      : LLADEARKAGVVYSLAYGDQPALTAELVDWARATGFHVVAAG--KGTKYLPAYHDVTPDGVWQHYGLTAGEAQ-SA------------GMNPQMFN : 206
Bradyrhizobium_sp._ORS278                     : LLADEARKAGVVYSLAYGDQPALTAELVDWARATGFHVVAAG--KGTKYLPAYHDVTPDGVWQHYGLTAGEAQ-SG------------GMNPQMFN : 212
Candidatus_Pelagibacter_ubique_HTCC1062       : YLADLAKENNVICSMAYGDQPSLIMEQIEWAKLNGFFVVCAG--KGTKYHPDFEYSTPDTVWGHYGLSKERAEIES------------GMNPKMFN : 204
Cupriavidus_necator_N-1                       : LLAHRAREAGVVYSLAYGDQPGLACELVDWARTCGFPIIAAG--RGHKWLPKFRESTPDTVWDHWGVTAEQAR-IG------------GMNPKMFN : 218
Cupriavidus_taiwanensis                       : LLAHRAREAGVVYSLAYGDQPGLACELVDWARTCGFPIIAAG--RGHKWLPEFRESTPDTVWDHWGVTAEQAR-VG------------GMNPKMFN : 218
Methylibium_petroleiphilum_PM1                : LLARKAREAGIVYSLAYGDQPALICEMVDWARTAGFQVVAAG--KGTKYLPSYHASTPDTVWNHYGLSADDAR-AG------------GMNAQMFN : 218
Methylobacterium_nodulans_ORS_2060            : LLAERARQKGVVYSMAYGDQPAIICELVDWARSCGFELTSAG--KGMNFEPRYRYSTPDTVWGFFGWSEEEVR-KG------------DFNPKMYN : 218
Methylobacterium_radiotolerans_JCM_2831       : LLARRAAEAGVIYSMASGDQPALIAELVDWARTIGLEVICAG--KGTKYLPAYHASTPDTVWGHYGFSESQVA-GG------------DFNPQMFN : 218
Methylobacterium_sp._4-46                     : LLAERARRKGVVYSMAYGDQPAIICELVDWARSCGFELTSAG--KGMNFEPRYRYSTPDTVWGFFGWSEEEVR-KG------------DFNPKMYN : 218
Mycobacterium_smegmatis_str._MC2_155          : ILQRRAAAAGVIYAMAYGDQPALICELVDWCRTVGFDVVAAG--KGTKYLPEYNYSTPETVWNYYGFTDEQLA-SG------------DYNPQMFN : 218
Nocardiopsis_dassonvillei_subsp._dassonv      : ALADLARERGVVYTPGIGDQPANLIEWYERTRLLGLDVVAIG--KSGEYDLVFDPATGRVRQLDQEIDAPDLADLL--TLGEDVPATLAARARAVA : 229
Paracoccus_denitrificans_PD1222               : GLAALAREKGCVVSPVDGDQPSLLIELITWAELLGFEIIAAG--KSSEYDFIFDAAAGTLTSDGRMVSAPDFA-QWADLDDRPAAEVVAGRANAAS : 230
Polaromonas_sp._JS666                         : LLARKAAEAGVVYSLAFGDQPALICDLVDWARTCGFPVVAAG--RGHKWLPHFCESTPDTVWGNYGLTPEQAL-RG------------GLNPKMFN : 218
Polymorphum_gilvum_SL003B-26A1                : LLACRAQAAGLVYSLAWGDQPALIAEHVDWARTCGFEVVCAG--KGTRYLPHYHQLTPDTVWETLQSYLALKD-PS------------QINLKMFN : 218
Polynucleobacter_necessarius_subsp._asym      : LLARKAAEAGVIYSMASGDQPALIAELVDWARTIGLEVVCAG--KGTKYLPIYHQSTPDTVWGHYGFSEEQVA-GG------------DFNAQMFN : 218
Pusillimonas_sp._T7-7                         : LLGLKAAQAGVVYSLAFGDQPALICDLVDWARTCGFPVVAAG--RGHKWLPHFSQSTPDTVWGYYGLTPEQAE-RG------------GLNPKMFN : 218
Rhodopseudomonas_palustris_BisB5              : LLADEARRAGVVYSLAYGDQPALTAELVDWARATGFRVVAAG--KGTKYLPIYHDVTPAGVWSHYGLSAAEAQ-SA------------GMNPQMFN : 206
Rhodospirillum_rubrum_ATCC_11170              : QLFKIARDNGVVYTTADGDQPSNLIGLVTWARILGFEIVAAG--KSSEYDYVFDPKSAQLTYSGQTIDVPALRDLW--TLGDDVPALMKARSAAIA : 231
Spirochaeta_smaragdinae_DSM_11293             : LLAKKAREQGVIYSLAEGDQPALLIGLVTWARKAGLSIVSIG--KSSEYDFIFDPERSTMQVLDRTEEVSGFEKVW--SLGENSRQTVEQRSALLS : 230
Spirochaeta_sp._Buddy                         : YLYNLAKHHKCVYAIAAGDQPANLIQLLSWVKTLGLEIIACG--KSSEYDFVYDLESGSFEFQGQKADLPELATCW----HLESMQTLKERSRILS : 232
Streptomyces_flavogriseus_ATCC_33331          : HLADLARDNGVVYTTADGDQPANLIALVTWAELLGLDIVAIG--KSSEYDLVFDPETGSVTQLDTTVPAPELAGLL--GLGSDVRATLAARAAAVS : 231
Streptomyces_sp._SirexAA-E                    : HLADLARDNGVVYTTADGDQPANLIALVTWAELLGLDIVAIG--KSSEYDLVFDPATGAVTQLDTTVPAPELAGLL--ALGSDVRATLAARAAAVT : 231
Variovorax_paradoxus_EPS                      : LLAHKAQQAGVIYSLAFGDQPALICDLVDWARTCGFPVVAAG--RGHKWLPHFTESTPETVWGNYGLTPEQAL-RG------------GLNPKMFN : 218
Variovorax_paradoxus_S110                     : LLARRAQQAGVVYSLAFGDQPALICDLVDWARTCGFPVVAAG--RGHKWLPHFTESTPETVWGNYGLTPEQAL-RG------------GLNPKMFN : 218
Xanthobacter_autotrophicus_Py2                : LLARHAAAAGIVYSFAYGDQPALIAEIVDWARTAGLEVICAG--KGTKYLPAYHQSTPETVWGHYGFSPEEVA-GG------------DFNAQMFN : 218
```

```
                                                    *      300         *      320         *      340         *      360         *      380
Anaerococcus_prevotii_DSM_20548              : SFVDGTNTML-ELNSVSNAL-GFLPDVFGCHGIDT-S---------PETAVEDFRLKAD--GGKLSRYGVVEFSRG------MAP-----GVFIIV : 286
Bacillus_clausii_KSM-K16                     : AFQDGTKTMA-EMNLLSNAI-GLVPDKVGMHGVDA-N---------LDNVAKKLDLKEH--GGVLDSLGVVEYVNG------LAP-----GVFVIV : 270
Bacillus_halodurans_C-125                    : SFQDGTKTMV-EMTAVANAT-GFLPDKPGMNGFVG-T---------VEELPAIFRRKEE--GGQVENERIVEYING------VAP-----GVFAIV : 286
Bacillus_pumilus_SAFR-032                    : AFQDGTKTMA-EMNLLSNAT-GLLPDKVGMHGVEA-N---------LENVADKLNQKQQ--GGVLDNFGVVEYVNG------LAP-----GVFVIV : 269
Bartonella_bacilliformis_KC583               : EFIDGSKTMV-EMAAIANAT-GLVPDCAGMHGPQA-A---------LEELDRVFIPQQD--GGILNRCGVVDYSIG----RGVSP-----GVFVIA : 294
Bartonella_clarridgeiae_73                   : EFIDGSKTMV-EMAAIANAT-GLLPDCPGMHGPKA-A---------LKDLNKVLIPKKD--GGILNRCGVVDYSIG----EGVAP-----GVFVIA : 294
Bartonella_grahamii_as4aup                   : EFIDGSKTMV-EMAAIANAT-GLLPDCPGMHGPQA-A---------LQDLNKVLIPKQD--GGILQRYGIVDYSTG----QGVSP-----GVFVIA : 294
Bartonella_henselae_str._Houston-1           : EFIDGSKTMV-EMAAIANAT-GLLPDCPGMHGPQA-A---------LKDLNKILIPKQD--GGILEQCGVVDYSIG----PGVSP-----GVFVIA : 294
Bartonella_quintana_str._Toulouse            : EFIDGSKTMV-EMAAIANAT-GLLPDCSGMHGPQV-M---------LQDLNKVLIPKQD--GGILKQCGVVDYSIG----QGVSP-----GVFVIA : 294
Bartonella_tribocorum_CIP_105476             : EFIDGSKTMV-EMAAIANAT-GLLPDCPGMHGPQA-A---------LQDLNKVLIPKQD--GGILQQYGVVDYSIG----QGVSP-----GVFVIA : 294
Brucella_abortus_bv._1_str._9-941            : EFIDGSKTMV-EMAAIANAT-GLVPDIAGMHGPRA-S---------IDQLSHTLIPQAE--GGVLSKSGVVDYSIG----KGVSP-----GVFVVA : 294
Brucella_abortus_S19                         : EFIDGSKTMV-EMAAIANAT-GLVPDIAGMHGPRA-S---------IDQLSHTLIPQAE--GGVLSKSGVVDYSIG----KGVSP-----GVFVVA : 294
Brucella_canis_ATCC_23365                    : EFIDGSKTMV-EMAAIANAT-GLVPDIAGMHGPRA-S---------IDQLSHTLIPQAD--GGVLSKSSVVDYSIG----KGVSP-----GVFVVA : 294
Brucella_melitensis_ATCC_23457               : EFIDGSKTMV-EMAAIANAT-GLVPDIAGMHGPRA-S---------IDQLSHTLIPQAE--GGVLSKSGVVDYSIG----KGVSP-----GVFVVA : 294
Brucella_melitensis_biovar_Abortus_2308      : EFIDGSKTMV-EMAAIANAT-GLVPDIAGMHGPRA-S---------IDQLSHTLIPQAE--GGVLSKSGVVDYSIG----KGVSP-----GVFVVA : 294
Brucella_melitensis_bv._1_str._16M           : EFIDGSKTMV-EMAAIANAT-GLVPDIAGMHGPRA-S---------IDQLSHTLIPQAE--GGVLSKSGVVDYSIG----KGVSP-----GVFVVA : 290
Brucella_microti_CCM_4915                    : EFIDGSKTMV-EMAAIANAT-GLVPDIAGMHGPRA-S---------IDQLSHTLIPQAD--GGVLSKSGVVDYSIG----KGVSP-----GVFVVA : 294
Brucella_ovis_ATCC_25840                     : EFIDGSKTMV-EMAAIANAT-GLVPDIAGMHGPRA-S---------IDQLSHTLIPQAD--GGVLSKSGVVDYSIG----KGVSP-----GVFVVA : 294
Brucella_pinnipedialis_B2/94                 : EFIDGSKTMV-EMAAIANAT-GLVPDIAGMHGPRA-S---------IDQLSHTLIPQAD--GGVLSKSGVVDYSIG----KGVSP-----GVFVVA : 294
Brucella_suis_1330                           : EFIDGSKTMV-EMAAIANAT-GLVPDIAGMHGPRA-S---------IDQLSHTLIPQAD--GGVLSKSGVVDYSIG----KGVSP-----GVFVVA : 294
Brucella_suis_ATCC_23445                     : EFIDGSKTMV-EMAAIANAT-GLVPDIAGMHGPRA-S---------IDQLSHTLIPQAD--GGVLSKSGVVDYSIG----KGVSP-----GVFVVA : 294
Chelativorans_sp._BNC1                       : EFVDGSKTMV-EMAAIANAT-GLVPDKPGMHGPAA-T---------LGELNRTLIPQKD--GGVLSRAGVVDYSIG----KGVAP-----GVFVIA : 294
Chloroflexus_aurantiacus_J-10-fl             : SFVDGTKTMV-EMTAIGNAL-GYAPEVTGGYGPSC-T---------VADLPKVFIPKAL--GGIFNNTKVVDFAVG-----DVAP-----GVFVVI : 287
Chloroflexus_sp._Y-400-fl                    : SFVDGTKTMV-EMTAIGNAL-GYAPEVTGGYGPSC-T---------VADLPKVFIPKAL--GGIFNNTKVVDFAVG-----DVAP-----GVFVVI : 287
Clostridium_difficile_630                    : SFKEGTKTMV-EMALMCNAT-GFVPDVRGGHGIEA-T---------VNEVPKKYALKSE--GGVLDNYGVVDFVNG------IAP-----GVFVVV : 286
Clostridium_difficile_CD196                  : SFKEGTKTMV-EMALMCNAT-GFVPDVRGGHGIEA-T---------VNEVPKKYALKSE--GGVLDNYGVVDFVNG------IAP-----GVFVVV : 286
Clostridium_difficile_R20291                 : SFKEGTKTMV-EMALMCNAT-GFVPDVRGGHGIEA-T---------VNEVPKKYALKSE--GGVLDNYGVVDFVNG------IAP-----GVFVVV : 286
Eubacterium_limosum_KIST612                  : AFKDGTKTMV-EMTAMANAT-GFVPDVTGAHGAES-D---------VAHLNDVLSLKSEGRGGVLDNYGVIEYING------VAP-----GVFVII : 289
Finegoldia_magna_ATCC_29328                  : SFVDGTNTMI-ELTAVANAL-GFTPDVLGCHGITT-N---------IHDIADKFKLKEQ--GGILNNYNIVDFAFG------VAP-----GVFAIV : 286
Halanaerobium_hydrogeniformans               : CFMDGTKSML-EMAAVSNAT-GLVPDVSGMHGPKT-T---------IEDLPKVYSLEED--GGILQNEGIVDFALGN-----VAP-----GVFVIV : 287
Listeria_innocua_Clip11262                   : AFQDGTKTMA-EMNLLSNAI-GYVPDVVGMHGISG-D---------VDSVIKDLDLKEQ--GGILNQFGVVEYVDG------LAP-----GVFVIV : 270
Listeria_ivanovii                            : AFQDGTKTMA-EMNLLSNAI-GYVPDVVGMHGISG-D---------VDSVIKDLDLKEQ--GGILNKFGVVEYVDG------LAP-----GVFVIV : 270
Listeria_monocytogenes                       : AFQDGTKTMA-EMNLLSNAI-GYVPDVVGMHGISG-D---------VDSVIKDLDLKDQ--GGILNKFGVVEYVDG------LAP-----GVFVIV : 270
Listeria_monocytogenes_08-5923               : AFQDGTKTMA-EMNLLSNAI-GYVPDVVGMHGISG-D---------VDSVIKDLDLKDQ--GGILNKFGVVEYVDG------LAP-----GVFVIV : 270
Listeria_monocytogenes_EGD-e                 : AFQDGTKTMA-EMNLLSNAI-GYVPDVVGMHGISG-D---------VDSVIKDLDLKDQ--GGILNKFGVVEYVDG------LAP-----GVFVIV : 270
Listeria_monocytogenes_HCC23                 : AFQDGTKTMA-EMNLLSNAI-GYVPDVVGMHGISG-D---------VDSVIKDLDLKDQ--GGILNKFGVVEYVDG------LAP-----GVFVIV : 270
Listeria_monocytogenes_serotype_4b_str._     : AFQDGTKTMA-EMNLLSNAI-GYVPDVVGMHGISG-D---------VDSVIKDLDLKDQ--GGILNKFGVVEYVDG------LAP-----GVFVIV : 270
Listeria_monocytogenes_serotype_4b_str._     : AFQDGTKTMA-EMNLLSNAI-GYVPDVVGMHGISG-D---------VDSVIKDLDLKDQ--GGILNKFGVVEYVDG------LAP-----GVFVIV : 270
Listeria_welshimeri_serovar_6b_str._SLCC     : AFQDGTKTMA-EMNLLSNAI-GYVPDVVGMHGISG-D---------VDSVIKDLDLKEQ--GGILNKFGVVEYVDG------LAP-----GVFVIV : 270
Mesorhizobium_ciceri_biovar_biserrulae_W     : EFVDGSKTMV-EMAAIANAT-GLVPDKAGMHGPAA-T---------LGELSKVLVPQKD--GGVLSKVGVVDYSIG----KGVAP-----GVFVVA : 294
Mesorhizobium_loti_MAFF303099_1              : EFVDGSKTMV-EMAAIANAT-GLVPDKAGMHGPAA-T---------LGELSKVLVPEKD--GGVLSRVGVVDYSIG----KGVAP-----GVFVVA : 294
Mesorhizobium_loti_MAFF303099_2              : EFVDGSKTMV-EMCAIANAT-GLVPDVPGMHGPKA-D---------RDDLVKVLIPRED--GGLLLKKGVVDYTIG----KGVAP-----GVFVIV : 287
Mesorhizobium_opportunistum_WSM2075          : EFVDGSKTMV-EMAAIANAT-GLVPDKAGMHGPAA-T---------LGELSKVLVPQKD--GGVLSRVGVVDYSIG----KGVAP-----GVFVVA : 294
Ochrobactrum_anthropi_ATCC_49188_1           : EFVDGSKTMV-EMAAIANAT-GLVPDIAGMHGPKA-A---------IDELNRTLIPKED--GGVLNKTGVVDYSIG----RGVSP-----GVFVIA : 294
Ochrobactrum_anthropi_ATCC_49188_2           : EFVDGSKTMV-EMAAIANAT-GLVPDKAGMHGPAA-T---------LDQLNKTLIPEKD--GGVLSKVGVVDYSIG----KGVAP-----GVFVVA : 294
Pelagibacterium_halotolerans_B2              : EFIDGSKTMV-EMAAIANAT-GLVPDRPGMHGPNA-D---------RETLPKVLIPRAD--GGVLERSGVVDYTIG----KGVAP-----GVFVVA : 295
Roseobacter_litoralis_Och_149                : EFVDGSKTAV-EMCAIGNAT-GLIPDCDGMHGPAA-G---------PKDLAKTLIPKKD--GGLLEGIGRVDYSIG----KGVSP-----GVFVII : 294
Sebaldella_termitidis_ATCC_33386             : SFKEGSKTMV-ELAVMSNST-GYIPDVRGAHGIKG-E---------VNDVPKVFSLKEE--GGILSKYGVVDFVDG------IAP-----GVFVVV : 286
Sinorhizobium_fredii_NGR234                  : EFVDGSKTMV-EMAAIANAT-GLVPDKPGMHGPAA-T---------LDKLSSVLIPKKD--GGVLSKAGVVDYSVG----KGVAP-----GVFVVA : 294
Starkeya_novella_DSM_506                     : EFVDGSKTMV-EMTALANAT-GLVPDVPGMHGPHA-D---------RDVLAQVLCTKED--GGILNRAGVVDFTLG----KGVAP-----GVFCIV : 296
Tepidanaerobacter_sp._Re1                    : SFVDATNTMI-ELTAVSNST-GFVPSQRGLIGPYA-T---------LKDIPKLFSLKEQ--GGLLDKHGVVDYAFG------IAP-----GVFVVI : 286
Thermosediminibacter_oceani_DSM_16646        : SFKDGTKTMV-ELTAVSNAT-GFLPTRRGLIGPKA-T---------VKELPEIFRLKEE--GGILDRYQVVEYVNG------VAP-----GVFVIV : 286
Verminephrobacter_eiseniae_EF01-2            : EFVDGSKTAV-EMSAIANAT-GLVPDIPGMHGPAA-S---------LDDLHKVLCPVAD--GGLLQRKGVVDYTIG----KGVAP-----GVFVVA : 293
Anabaena_variabilis_ATCC_29413               : SFADGSKISF-EQAIVANAT-GMKVAKRGMLGYDFTG--------HVDEMTNMYDVEQL-----KQLGGIVDYVVG----AKPGP-----GVYVFA : 285
Chloroflexus_aggregans_DSM_9485              : SFADGTKISF-EQAVVANAT-GMRVARRGMFGPTVPS------GTPLADVVHDLYPLEA----LIEGPGIVDYVVG----ATPGP-----GVFVLG : 290
Coraliomargarita_akajimensis_DSM_45221       : SFADGTKVSL-EQASIANAT-GMQVARRGMIAIEYDG--------MVDDLTGRYDVDEL-----KALGGIVEMVVG----GKPGP-----GVFVFA : 285
Coxiella_burnetii_CbuG_Q212                  : AYSDGTKVNI-EMALIANGT-NLIPTKPGMEGPVAKH---------VSEAVNLFDFDAY------GEKGRVDYILG----AQPGG-----GVYVIG : 262
Coxiella_burnetii_CbuK_Q154                  : AYSDGTKVNI-EMALIANGT-NLIPTKPGMEGPVAKH---------VSEAVNLFDFDAY------GEKGRVDYILG----AQPGG-----GVYVIG : 262
Coxiella_burnetii_Dugway_5J108-111           : AYSDGTKVNI-EMALIANGT-NLIPTKPGMEGPVAKH---------VSEAVNLFDFDAY------GEKGRVDYILG----AQPGG-----GVYVIG : 262
Coxiella_burnetii_RSA_331                    : AYSDGTKVNI-EMALIANGT-NLIPTKPGMEGPVAKH---------VSEAVNLFDLDAY------GEKGRVDYILG----AQPGG-----GVYVIG : 262
Coxiella_burnetii_RSA_493                    : AYSDGTKVNI-EMALIANGT-NLIPTKPGMEGPVAKH---------VSEAVNLFDFDAY------GEKGRVDYILG----AQPGG-----GVYVIG : 262
Cyanothece_sp._PCC_7425                      : SFADGTKISF-EQAIVANAT-GMQVAQRGMLGYDHPG--------HVDELVHRYDIEQL-----QSLGGIVDYVVG----AQPGP-----GIFVLA : 285
Cyclobacterium_marinum_DSM_745               : SFADGSKISF-EQAIIANAT-GMGVAKRGMHGPTVAS------GKPITECVDEYPLEDI-----LAGDGIVDYIVG----ASPGP-----GVFVLG : 287
Deinococcus_maricopensis_DSM_21211           : SFADGTKISF-EQAIVANGT-GMRVAQRGMRGLDFPG--------HVDDLKALYDADEL-----RRLGGVVDYVVG----AKPGP-----GVFVFA : 285
Desulfococcus_oleovorans_Hxd3                : SYTDGTKLNI-EMAIIANAC-GLITTTPGMHGPRAAH---------VQDVFNCFDFDAL----WKDRRPFVDYILG----AEPGG-----GVFVIG : 277
Frankia_sp._EuI1c                            : SFTDGTKLQI-EQALVANGL-GATIARRGLLGPRT------------DSVPTGALPLAE--AAATLGTPIADYVLP----ERGNP-----GVFVVA : 264
Gloeobacter_violaceus_PCC_7421               : SFADGTKISF-EQAIVANAT-GMGVAQRGMLGYEHRG--------HVDELTRVYDIAQL-----KAWGGIVDYVVG----ASPGP-----GVFVLA : 285
Hirschia_baltica_ATCC_49814                  : SFADGTKVAF-EQALVANAF-DMRVAKRGMIGPDFSGGDPYAPKVPLEECLDVYKPYLG-----PDKPALVEYVVG----AAPPA-----GVFVLG : 292
Kineococcus_radiotolerans_SRS30216           : SFADGTKVSF-EQALVANAA-GFSVEKRGMRGADHRG--------HVDELTTHYDVDQL-----KELGGVVDYVVG----SKPGP-----GVYVLG : 285
Methanosphaerula_palustris_E1-9c             : AFTDGTKVQI-EQALVANGL-GAQILQDGMLGPEA------------DDIQSGGLILAE--AAKKMGTKVSDYILA---PGAPA------GVFIVA : 241
Nakamurella_multipartita_DSM_44233           : SFADGTKVSF-EQAIVANAT-GMTVERRGMRGADHRG--------HVDELTGAYDVEQL-----RELGGVVDYVVG----AKPGP-----GVFCLA : 285
Nostoc_azollae_0708                          : SFADGTKISF-EQAIVANAT-GMKVAKRGMLGYDYTG--------HVDEMTKMYDVDQL-----KELGGIVDYVVG----TKPGP-----GVFVFA : 285
Nostoc_punctiforme_PCC_73102                 : SFADGTKISF-EQAIVANAT-GMKVAKRGMLGYNFNG--------YVDEMAHIYDVEQL-----KELGGIVDYVVG----AKPGP-----GVYVFA : 285
Nostoc_sp._PCC_7120                          : SFADGSKISF-EQAIVANAT-GMKVAKRGMLGYDFTG--------HVDEMTNMYDVEQL-----KQLGGIVDYVVG----AKPGP-----GVYVFA : 285
Pseudomonas_stutzeri_A1501                   : SFTDGTKVQI-EQVIIGNGL-GATITRQGMEGLASTN---------LDDSASLLGMMAE-----RVGQPIVDYVIP---SGYPAG-----GVFLVG : 230
Pseudomonas_stutzeri_ATCC_17588__LMG_111     : SFTDGTKVQI-EQVIIGNGL-GATITRQGMEGLASTN---------LDDSASLLGMMAE-----RVGQPIVDYVIP---SGYPAG-----GVFLVG : 230
Pseudoxanthomonas_spadix_BD-a59              : SFADGTKISF-EQAIVANGT-GMSILKRGMTGLDHRG--------HVDELTRSYDIDAL-----KAVGGAVDYVVG----AQPGP-----GVYVLA : 283
Ramlibacter_tataouinensis_TTB310             : SFADGTKISF-ENAIVANGT-GMRVGQRGMFGPTVPA------GTPIQQVASLYPVDAL-----LEGPGIVDYVVG---AM-PGP-----GVYVLG : 287
Rhodobacter_sphaeroides_2.4.1                : SFADGTKISY-EQAIVANGT-GMRVAKRGMIGIDPTNKNPTLPLRPLEDYVAMFAPHLD-----PEGPGIVDYIVG----ARPGP-----GVFVIG : 294
Rhodobacter_sphaeroides_ATCC_17025           : SFADGTKISY-EQAIVANGT-GMRVARRGMIGIDPTGKNPTLPLRPLEEYVAMFAPHLD-----PEGPGIVDYVVG----ARPGP-----GVFVIG : 294
Rhodobacter_sphaeroides_ATCC_17029           : SFADGTKISY-EQAIVANGT-GMRVAKRGMIGIDPTNKNPTLPLRPLEDYVAMFAPHLD-----PEGPGIVDYIVG----ARPGP-----GVFVIG : 294
Rhodobacter_sphaeroides_KD131                : SFADGTKISY-EQAIVANGT-GMRVAKRGMIGIDPTNKNPTLPLRPLEDYVAMFAPHLD-----PEGPGIVDYIVG----ARPGP-----GVFVIG : 294
Rhodothermus_marinus_DSM_4252                : AFTDGTKVQI-EQALVANGL-GADIAVRGMLGIQS------------PDVEVGARTLAD--GARRLGHPISDYVLS---PGAPA------GVFIAA : 241
Rhodothermus_marinus_SG0.5JP17-172           : AFTDGTKVQI-EQALVANGL-GADIAVRGMLGIQS------------PDVEVGARTLAD--GARRLGHPISDYVLS---PGAPA------GVFIAA : 245
Sphingomonas_wittichii_RW1                   : SFADGTKISY-EQAIVANGT-GFSVARRGMIGPDFSGGDPTAPLVPLEETVSAFADALD-----GASAGLVDYVVG----ARPGP-----GVFVLG : 292
Streptomyces_griseus_subsp._griseus_NBRC     : SFTDGTKVSF-EQAIVANAF-GFTVPRRGMYGRDHSG--------HIDELTQMYDIDEL-----RAYGGIVDYVVG----AKPAP-----GVYVLG : 284
Xanthomonas_campestris_pv._campestris_st     : SFADGTKISF-EQAIVANGT-GMSILQRGMTGYEHHG--------HVDQLTQRYDIDAL-----RAVCGAVDYVVG----ALPSP-----GVFVLA : 283
Xanthomonas_campestris_pv._campestris_st     : SFADGTKISF-EQAIVANGT-GMSILQRGMTGYEHHG--------HVDQLTQRYDIDAL-----RAVCGAVDYVVG----ALPSP-----GVFVLA : 283
Xanthomonas_campestris_pv._campestris_st     : SFADGTKISF-EQAIVANGT-GMSILQRGMTGYEHHS--------HVDQLTQRYDIDAL-----RAVGGAVDYVVG----ALPSP-----GVFVLA : 283
Achromobacter_xylosoxidans_A8                : SFLDGTKSAI-EMAAVSNAT-GLLPSPSGLHFPPC-G---------VDDLARVLRPREN--GGILHHRGQVEVISSLERDGRPVFRDLRWGVYVTL : 300
Acidiphilium_cryptum_JF-5                    : SFLDGTKSAI-EMAAIANAT-GLAVPSSGLHFPAC-G---------VDDLPHLLRPRAA--GGVLERAGMVEVVSSLERDGRPVFRDLRWGVYAVF : 289
Acidiphilium_multivorum                      : SFLDGTKSAI-EMAAIANAT-GLAVPSSGLHFPAC-G---------VDDLPHLLRPRAA--GGVLERAGMVEVVSSLERDGRPVFRDLRWGVYAVF : 289
Acidovorax_ebreus_TPSY                       : SFLDGSKPSI-ESTAVSNAT-GLAVPSNGLQYPPA-S---------VEDIPYVTRPVGE--GGVLEKKGMVEVISSLEKDGRRIPYDIRMGVWVTV : 300
Acidovorax_sp._JS42                          : SFLDGSKPSI-ESTAVSNAT-GLAVPSNGLQYPPA-S---------VEDIPYVTRPVGE--GGVLEKKGMVEVISSLEKDGRRIPYDIRMGVWVTV : 300
Actinosynnema_mirum_DSM_43827                : SLKRSAAADLCEMTVVATRC-GAVPDREDLHYPVA-R---------VTELADVYAAREH--GGLLGRPGVIDVFSA---LRLPDEASFAGGVFAVV : 288
Agrobacterium_sp._H13-3                      : SFTDGTKAAI-EMAAVANGT-GLDCPDDGLAFPPA-G---------LHDLARVFRPAAD--GGRMSRSGLVDIAASQEPDGREVFNNIRYGVFVTF : 300
Agrobacterium_tumefaciens_str._C58           : SFTDGTKAAI-EMAAVANGT-GLDCPDDGLAFPPA-G---------LHDLARVFRPAAD--GGRMSRSGLVDIAASQEPDGREVFNNIRYGVFVTF : 337
Anaeromyxobacter_sp._Fw109-5                 : SFLDGTKSAI-EMAAVSNAT-GLTPAPEGLAFPPA-G---------ADDLARVLRPRAA--GGVLHHAGQVEVVSSLARDGSPVPRDLRWGVYVVI : 300
Arthrobacter_sp._FB24                        : SFRDGTKASI-EMAAVANGA-GLVPSDTGLTFTPG-D---------VEEIATICRPADA--GGVLAHEGTVDVMSSVKRDGTWIPHNTQEGVFVVV : 298
Azorhizobium_caulinodans_ORS_571             : SFTDGTKAAI-EMAAVANGT-GLDCPDDGLAFPPT-G---------LHDLARVFRPVSD--GGRLPRSGLVDIAASQEPDGREVFNNIRYGVFVTF : 300
Bordetella_avium_197N                        : SFLDGTKSAL-EMASVANAT-GLTPAPQGLFYPPC-G---------VDDLARVLRPREH--GGILHHRGQVEVVSSLERDGRSVFRDLRWGVFVTL : 300
Bordetella_bronchiseptica_RB50               : SFLDGTKSAI-EMAAVSNAT-GLLPSPTGLHFPPC-G---------VDDLARVLRPREE--GGMLHHRGQVEVVSSLERDGRPVFRDLRWGVYVTL : 300
Bordetella_parapertussis_12822               : SFLDGTKSAI-EMAAVSNAT-GLLPSPTGLHFPPC-G---------VDDLARVLRPREE--GGMLHHRGQVEVVSSLERDGRPVFRDLRWGVYVTL : 300
Bordetella_petrii_DSM_12804                  : SFLDGTKSAI-EMAAVANAT-GLLPAPSGLHFPPC-G---------VDDLARVLRPRDD--GGMLHHRGQVEVISSLERDGRPVFRDLRWGVYVTL : 300
Bradyrhizobium_japonicum_USDA_110            : SFLDGTKSAI-EMAAIANAC-GLDVPAEGLLFPPC-G---------VDDLPHIMRPRSR--GGVLERSGVVEVVSSLERDGRPVFRDLRWGVYVVL : 292
Bradyrhizobium_sp._BTAi1                     : SFLDGTKSAI-EMAAIANAT-GLDVPNDGLLFPPC-G---------VDDLPHVLRPRER--GGVLERSGMVEVVSSLERDGRPVFRDLRWGVYVVL : 288
Bradyrhizobium_sp._ORS278                    : SFLDGTKSAI-EMAAIANAT-GLDVPSDGLLFPPC-G---------VDDLPQVLRPRER--GGVLERSGMVEVVSSLERDGRPVFRDLRWGVYVVL : 294
Candidatus_Pelagibacter_ubique_HTCC1062      : SFLCGDKSAI-EMCAVSNAS-DLKCPSNGLTFPPV-G---------VYDIAKKLIPKVE--GGLIDYEGQVEVISSIDLNQKDIPNDLRWGVYIVI : 286
Cupriavidus_necator_N-1                      : AFLDGSKPAI-ESTAIANAT-GLQVQAEGITFAPA-S---------VEQIPTVMRLKSD--GGVLSQKGVVDVVSSLNTDGTSVGYDIRKGVWVCV : 300
Cupriavidus_taiwanensis                      : AFLDGSKPAI-ESAAIANAT-GLQVQAEGITFAPA-S---------VEKIPTVMRLQSD--GGVLSAKGVVDVVSSLNPDGSPVGYDIRKGVWVCV : 300
Methylibium_petroleiphilum_PM1               : SFLDGTKSAI-EMAAVSNAC-GLTPADDGLAFPPC-G---------VDDLARLLKPRAD--GGLLQHRGQVEVISSLERDGRPVFRDLRWGVYVTF : 300
Methylobacterium_nodulans_ORS_2060           : SFTDGTKAAI-EMAAVANGT-GLDCPDDGLAFPPT-G---------LHDLARVFRPVAD--GGRLPRSGLVDIAASQEPDGREVFNNIRYGVFVTF : 300
Methylobacterium_radiotolerans_JCM_2831      : SFLDGTKSAL-EMAAVANAC-GLTPPRDGLAFPPC-G---------VDDLPGILKPAAD--GGLLTERGTVEVVSSVERDGRPVFRDLRWGVYAVF : 300
Methylobacterium_sp._4-46                    : SFTDGTKAAI-EMAAVANGT-GLDCPDDGLAFPPT-G---------LHDLARVFRPVAD--GGRLPRSGLVDIAASQEPDGREVFNNIRYGVFVTF : 300
Mycobacterium_smegmatis_str._MC2_155         : SFLDGTKSAI-EMAAVANAT-GLLPQEQGLLFPPA-S---------KDDLPTVLRENRF-PGGSLMRRGTVEIASSMYRDGQEVPDNLRWGVYVTF : 301
Nocardiopsis_dassonvillei_subsp._dassonv     : GLSRSAAADYCEMGVVANHT-GLVPDTEELHYPVA-R---------VAELADVYSLAED--GGILSRSGAVDVFSV---LRLPEEASFAGGVFAVV : 309
Paracoccus_denitrificans_PD1222              : ALPQRAVPDLCELTVVANAT-GFAPDAAPLHAPIA-R---------ITEVPTLFSTRDE--GGILDGRRVLDVFHC---LREPGEVSFAGGVFVVI : 310
Polaromonas_sp._JS666                        : SFLDGSKPSI-ECTAVANAT-GLTVPSNGLLYPPA-S---------VEDIPFVTRPIQE--GGVLERKGMVEVISSLEANGRKIPYDIRMGVWVTV : 300
Polymorphum_gilvum_SL003B-26A1               : SFLDGTKSGI-EMTAVCNAT-GLIPQDDGLGFPPA-S---------RFELSSVCRPKEY--GGTLSRTGTTEVVSSLARDGSDVPHHLAMGTYVVI : 300
Polynucleobacter_necessarius_subsp._asym     : SFLDGTKSAL-EMAAVSNGC-DLTPPSNGLEFPPC-G---------VDDLPHIFRPISE--GGILKQKGTVEVVSSVERDGRPVFRDLRWGVFAVF : 300
Pusillimonas_sp._T7-7                        : SFLDGSKPSI-ESSAVANAT-GLRVPSNGLLYPPA-S---------IEDIPFVTRPISE--GGVLESKGMVEVISSLEADGRHIPYDIRMGVWVTV : 300
Rhodopseudomonas_palustris_BisB5             : SFLDGTKSAI-EMAAIANAT-GLDVPAAGLAFPPC-G---------VDDLPHVLRPRGD--GGVLERSGMVEVVSSLERDGRPVFRDLRWGVYVVI : 288
Rhodospirillum_rubrum_ATCC_11170             : RLPQSAVPDYCEMNVVADST-GLTPACDALSYPLC-R---------ADELADVFAPRAD--GGLLDRSGVVDVFNC---LRRPDEASFGGGVFVVV : 311
Spirochaeta_smaragdinae_DSM_11293            : DFGQRAIPDLAEMSIVCNHLPEFTPDISSFHCPIA-R---------AVEIPDLMCPETM--GGLFSGEARIDVINC---LRRPDEQSLEGGVYVVV : 311
Spirochaeta_sp._Buddy                        : DFPQFAVPDYCEMNVVSNAT-ALRPSCDRFHYPVC-R---------VEELADIYIPIED--GGVLTKTGVVDVFNN---LRRPDEASFAGGVFVIV : 312
Streptomyces_flavogriseus_ATCC_33331         : ALPTGATADYCEMAVVATGT-GFRPDVERLHYPVA-R---------IAELADVYSLRED--GGVLHRSGAVDVFSA---LRLPDEASFAGGVFVVV : 311
Streptomyces_sp._SirexAA-E                   : ALPTGATADYCEMAVVATNT-GFRPDTERLHYPVA-R---------IAELADVYALRED--GGLLHRTGAVDVFSA---LRLPDEASFAGGVFVVV : 311
Variovorax_paradoxus_EPS                     : SFLDGSKPSI-ESSAVANAT-GLGVPSDGLLYPPA-S---------VEDIPFVTRPKSE--GGVLERKGMVEVISSLEADGRKIPYDIRMGVWVTV : 300
Variovorax_paradoxus_S110                    : SFLDGSKPSI-ESSAVANAT-GLTVPSDGLLYPPA-S---------VEDIPFVTRPRSE--GGVLERKGMVEVISSLEADGRKIPYDIRMGVWVTV : 300
Xanthobacter_autotrophicus_Py2               : SFLDGTKSAL-EMAAVANGC-DLEPAADGLSFPPC-G---------KDDLPTILRPRAD--GGILPHKGTVEVVSSLERDGRPVFRDLRWGVYATF : 300
```

```
                                                     *       400         *       420         *       440         *       460         *       480
Anaerococcus_prevotii_DSM_20548                : TSDQEDVRDLMKFL---GFGDGPNYLMYRPYHLTSLETPITIYKAVVENEATIVPLHGQVADTVTIAKRDIKAGERL-EGVGSK----TVYGKLTS : 374
Bacillus_clausii_KSM-K16                       : KSELEPVDEELRYLLKVNANHGPHYTLYRPYHLASLETPITIAKAVLHHDTSIHPLGAPVSETVAVAKRAIQPGERL-DGIGGY----SVRGMLET : 361
Bacillus_halodurans_C-125                      : KSDKEEVNHEMTYL---SMGEGPNYVLYRPYHLTSLETPISIARAHLYHEATIAPWQGLQAETVAVAKKDLAAGEFL-DSIGGF----TVYGTILT : 374
Bacillus_pumilus_SAFR-032                      : KSDLEPVDEELRYL---KVGKGPHYTLYRPFHLASLETPVTIAKAVLQHDSSIHPIGVPVSETVAVAKRDIKAGESL-DGIGGY----SVRGVLET : 357
Bartonella_bacilliformis_KC583                 : KIAHPRLRERMEDL---KIGRGPYFTFHRPYHLTAMEVPLTCARVMLYGKADMAPLSHPVAEVCAVAKKDLHPGDQL-DFIGLH----TYRAWTMS : 382
Bartonella_clarridgeiae_73                     : EITHPRLRERMEDL---KIGKGPYFTFHRPYHLTAMEIPLTCARIMLYGKTDMAPLKQPVAEVCAIAKKDLHPGEQL-DFIGLY----SYRAWTMS : 382
Bartonella_grahamii_as4aup                     : EIAHPRLRERMEDL---KIGQGPYFTFHRPYHLTAMEVPLTCARAMLYGKKDMVPLSHPVVEVCAVAKKDLHPGDKL-DFIGLY----TYRAWIMN : 382
Bartonella_henselae_str._Houston-1             : EIAHPRLRERMEDL---KMGQGPYFTFHRPYHLTAMEVPLTCARIMLYGKKDMAPLNHPVVEVCAVAKKDLYPGDQL-DFIGLY----SYRAWIMN : 382
Bartonella_quintana_str._Toulouse              : EIVHPRLRERMEDL---KMGQGPYFTFHRPYHLTAMEVPLTCARVMLYGKKDMAPLNHPVVEVCAVAKKNLYPGDQL-DFIGLY----SYRAWIMD : 382
Bartonella_tribocorum_CIP_105476               : EIAHPRLRERMEDL---KIGQGPYFTFHRPYHLTAMEVPLTCARVMLYGKKDMVPLSHPVVEVCAVAKKDLHPGDQL-DFIGLY----SYRAWIMN : 382
Brucella_abortus_bv._1_str._9-941              : KMDHPRLNERLEDL---KIGKGPYFTFHRPYHLTSLEVPLTVARVVLHGKTDMVPLPKPVAEVCAVAKKDMQPGEHL-DAIGQY----CYRSWIMT : 382
Brucella_abortus_S19                           : KMDHPRLNERLEDL---KIGKGPYFTFHRPYHLTSLEVPLTVARVVLHGKTDMVPLPKPVAEVCAVAKKDMQPGEHL-DAIGQY----CYRSWIMT : 382
Brucella_canis_ATCC_23365                      : KMDHPRLNERLEDL---KIGKGPYFTFHRPYHLTSLEVPLTVARVVLHGKTDMVPLPKPVAEVCAVAKKDMQPGEHL-DAIGQY----CYRSWIMT : 382
Brucella_melitensis_ATCC_23457                 : KMDHPRLNERLEDL---KIGKGPYFTFHRPYHLTSLEVPLTVARVVLHGKTDMVPLPKPVAEVCAVAKKDMQPGEHL-DAIGQY----CYRSWIMT : 382
Brucella_melitensis_biovar_Abortus_2308        : KMDHPRLNERLEDL---KIGKGPYFTFHRPYHLTSLEVPLTVARVVLHGKTDMVPLPKPVAEVCAVAKKDMQPGEHL-DAIGQY----CYRSWIMT : 382
Brucella_melitensis_bv._1_str._16M             : KMDHPRLNERLEDL---KIGKGPYFTFHRPYHLTSLEVPLTVARVVLHGKTDMVPLPKPVAEVCAVAKKDMQPGEHL-DAIGQY----CYRSWIMT : 378
Brucella_microti_CCM_4915                      : KMDHPRLNERLEDL---KIGKGPYFTFHRPYHLTSLEVPLTVARVVLHGKTDMVPLPKPVAEVCAVAKKDMQPGEHL-DAIGQY----CYRSWIMT : 382
Brucella_ovis_ATCC_25840                       : KMDHPRLNERLEDL---KIGKGPYFTFHRPYHLTSLEVPLTVARVVLHGKTDMVPRPKPVAEVCAVAKKDMQPGEHL-DAIGQY----CYRSWIMT : 382
Brucella_pinnipedialis_B2/94                   : KMDHPRLNERLEDL---KIGKGPYFTFHRPYHLTSLEVPLTVARVVLHGKTDMVPLPKPVAEVCAVAKKDMQPGEHL-DAIGQY----CYRSWIMT : 382
Brucella_suis_1330                             : KMDHPRLNERLEDL---KIGKGPYFTFHRPYHLTSLEVPLTVARVVLHGKTDMVPLPKPVAEVCAVAKKDMQPGEHL-DAIGQY----CYRSWIMT : 382
Brucella_suis_ATCC_23445                       : KMDHPRLNERLEDL---KIGKGPYFTFHRPYHLTSLEVPLTVARVVLHGKTDMVPLPKPVAEVCAVAKKDMQPGEHL-DAIGQY----CYRSWIMT : 382
Chelativorans_sp._BNC1                         : EMSHPRIRERMEDL---KMGEGPYFTFHRPYHLTSLEVPLTCARVVLYGKPDMVPLPKPVAEVCAVAKKDLNVGERL-DAIGEY----CYRAWIMR : 382
Chloroflexus_aurantiacus_J-10-fl               : TTDQPKIIRDLRYLRLLGHADHNYWTLYRPYHLANLEAPITVARVALEKERFLATSRSPVAETVAYAKRDLYPGEVI-DALGGF----TVYGMIEQ : 378
Chloroflexus_sp._Y-400-fl                      : TTDQPKIIRDLRYLRLLGHADHNYWTLYRPYHLANLEAPITVARVALEKERFLATSRSPVAETVAYAKRDLYPGEVI-DALGGF----TVYGMIEQ : 378
Clostridium_difficile_630                      : AHKLKAVNDELKYL---SMGDGPNYILYRPYHLCSLETPLSAAMAVLDHKATIVPKAGLVAEVMTIAKKDLKKGEFM-DGYGAY----TCYGTIEK : 374
Clostridium_difficile_CD196                    : AHKLKAVNDELKYL---SMGDGPNYILYRPYHLCSLETPLSAAMAVLDHKATIVPKAGLVAEVMTIAKKDLKKGEFM-DGYGAY----TCYGTIEK : 374
Clostridium_difficile_R20291                   : AHKLKAVNDELKYL---SMGDGPNYILYRPYHLCSLETPLSAAMAVLDHKATIVPKAGLVAEVMTIAKKDLKKGEFM-DGYGAY----TCYGTIEK : 374
Eubacterium_limosum_KIST612                    : GTDQPDIAAELTYL---SMGPGPNYTLYRPYHLTSLETPMSVAKACIDHEPTIVPRCGRVAETIAVAKKDMKAGEML-DGIGGY----TIRGTFMA : 377
Finegoldia_magna_ATCC_29328                    : THDTNEVHDLMEYL---SMGKGPNYTLYRPYHLTSLETPLTIYDAIVEKEPTIYPKYGQVSDTITVAKRDIKKGQKI-EGIGGH----DCFGKITS : 374
Halanaerobium_hydrogeniformans                 : TTDNERIKFSMNYS---KMGEGPNYLLYRPYHLASLETPLSAVRAVLYNEATIAPIGDIVTEVTTIAKKDLKAGEEI-DGIGGF----AVYGSVDK : 375
Listeria_innocua_Clip11262                     : KGQNEGVSHELSYL--MKKGERDHHILYRPYHLASLETPLTIAKAVLNHDHAIVPMGAPVSETVAVAKKDIAAGERL-DGIGGF----CVRGVLET : 359
Listeria_ivanovii                              : KGQNEGVSHELSYL--MKKGDRDHHILYRPYHLASLETPLTIAKAVLNHDHAIVPKGAPISETVAVAKKDIPSGERL-DGIGGF----CVRGVLET : 359
Listeria_monocytogenes                         : KGQNEGVSHELSYL--MKKGDRDHHILYRPYHLASLETPLTIAKAVLNHDHAIVPMGAPVSETVAVAKKDIAAGEKL-DGIGGF----CVRGVLET : 359
Listeria_monocytogenes_08-5923                 : KGQNEGVSHELSYL--MKKGDRDHHILYRPYHLASLETPLTIAKAVLNHDHAIVPMGAPVSETVAVAKKDIAAGEKL-DGIGGF----CVRGVLET : 359
Listeria_monocytogenes_EGD-e                   : KGQNEGVSHELSYL--MKKGDRDHHILYRPYHLASLETPLTIAKAVLNHDHAIVPMGAPVSETVAVAKKDIAAGEKL-DGIGGF----CVRGVLET : 359
Listeria_monocytogenes_HCC23                   : KGQNEGVSHELSYL--MKKGDRDHHILYRPYHLASLETPLTIAKAVLNHDHAIVPMGAPVSETVAVAKKDISTGEKL-DGIGGF----CVRGVLET : 359
Listeria_monocytogenes_serotype_4b_str._       : KGQNEGVSHELSYL--MKKGDRDHHILYRPYHLASLETPLTIAKAVLNHDHAIVPMGAPVSETVAVAKKDIAAGEKL-DGIGGF----CVRGVLET : 359
Listeria_monocytogenes_serotype_4b_str._       : KGQNEGVSHELSYL--MKKGDRDHHILYRPYHLASLETPLTIAKAVLNHDHAIVPMGAPVSETVAVAKKDIAAGEKL-DGIGGF----CVRGVLET : 359
Listeria_welshimeri_serovar_6b_str._SLCC       : KGQNEGVSHELSYL--MKKGDRDHHILYRPYHLASLETPLTIAKAVLNHDHAIVPMGAPVSETVAVAKKDIAAGERL-DGIGGF----CVRGVLET : 359
Mesorhizobium_ciceri_biovar_biserrulae_W       : DMSHPRISERMEDL---KMGKGPYFTFHRPYHLTSLEVPLTCARVVLYGKADMVPLAKPVAEVAAMAKKDMKPGEKL-DAIGEY----CYRAWIMT : 382
Mesorhizobium_loti_MAFF303099_1                : DMSHPRISERMEDL---KMGKGPYFTFHRPYHLTSLEVPLTCARVVLYGKADMVPLAKPVAEVAAVAKKDMQPGEKL-DAIGEY----CYRAWIMT : 382
Mesorhizobium_loti_MAFF303099_2                : EATHPRIIERMDDL---HIGHGPYYSLFRPYHLTSLEVPLTAARIVLFGKPDMVPLPRPVAEVCAVAKRDLAAGETF-DAIGET----CYRSWTMT : 375
Mesorhizobium_opportunistum_WSM2075            : DMSHPRISERMEDL---KMGKGPYFTFHRPYHLTSLEVPLTCARVVLYGKADMVPLAKPVAEVCAVAKKDMQPGEKL-DAIGEY----CYRAWIMM : 382
Ochrobactrum_anthropi_ATCC_49188_1             : KMEHPRLVERLEDL---KMGKGPYFTFHRPFHLTSLEVPLTVARVVLHGKTDMVPLPKPVAEVCAVAKKDLQPGDHL-DAIGQY----CYRSWIMT : 382
Ochrobactrum_anthropi_ATCC_49188_2             : DMSHPRISERMEDL---KMGKGPYFTFHRPYHLTSLEVPLTCARVVLYGKPDMVPLSKPVAEVAAVAKKDMQPGEKL-DAIGEY----CYRAWIMT : 382
Pelagibacterium_halotolerans_B2                : KATHPRVVERMDDL---HVGIGPYYGFYRPYHLTSLEVPLTAARIMLYGKPDMVPLANPVAEVCALAKRDLAVGEVF-DAIGET----CYRSWTMT : 383
Roseobacter_litoralis_Och_149                  : EAEHPRIRERLKDL---KMGDGPYFEFIRPYHLTSLEVPLTCARAVLYGKADMVPLDKPVAEVAAVAKRDLKAGETL-DQIGEY----TYRAWAME : 382
Sebaldella_termitidis_ATCC_33386               : SSKLEEVRKEMKYL---DMGDGPNYILYRPYHLCSLETPLSAAKAVIDHEATIAPMGGLVSEVITIAKKDLKAGENL-DGIGGY----TVYGSVEV : 374
Sinorhizobium_fredii_NGR234                    : DMSHPRISERMEDL---KMGKGPYFTFHRPYHLTSLEVPLTCARVVLYGKPDMVPLSTPVAEVCAVAKKDLAPGETL-DAIGEY----CYRAWIMT : 382
Starkeya_novella_DSM_506                       : KPRHPRVIERMADL---KVGPGPCFALIRPFHLTSLEVPLSAVRAVLHKRPDMEVLDHPVAECAAVAKRDLKPGDTL-GKIGEY----DYRGYAMG : 384
Tepidanaerobacter_sp._Re1                      : NSDYPVTKEIMSYI---SMGDGPNYVLYRPYHLVCIETPLSAARAFLYGEPTISPAGAPVSETVAVAKKDLKAGESL-DGIGGY----TVYGIIDT : 374
Thermosediminibacter_oceani_DSM_16646          : TTKLEAVREEMAYL---SMGEGPNYVFYRPYHLTSIETPLSAARAYIYGEPTIAPKGAPVSETIAVAKKDLKAGEHL-DGIGGF----TVYGVIDT : 374
Verminephrobacter_eiseniae_EF01-2              : EMAHPRLRERMDYL---QLGTGPYYTFYRPYHLCSLEVPLTCARAVLYGRADMAPIAKPTAEVCAVAKRDLYPGGRL-DAIGEY----TYRAWAMT : 381
Anabaena_variabilis_ATCC_29413                 : THDDPKQRHYLNLY---KLGEGPLYSFYTPYHLCHFEVPLSVARAVLFSDAVMAPLAGLSVDVITTAKIDLKAGETL-DGIGYY----MTYGQCEN : 373
Chloroflexus_aggregans_DSM_9485                : THDHPRMQHYLNLY---KLGKGPLYLFYTPYHLCHFEVPNSIARVALFGDQVLAAAGRPMVEVITSAKTDLHAGQTL-DGLGGY----MTYGLAEN : 378
Coraliomargarita_akajimensis_DSM_45221         : TTDDPVLQRFMEYG---KLGKGPLYSFYTPYHLLFIDLPFSIARLVDFDDGTLDARDQLSVEVLSTAKRDLKAGEVL-DGIGGF----TVYGECEN : 373
Coxiella_burnetii_CbuG_Q212                    : KCEDRLQESYLNYY--KVHNRYPYYVFYRPYHLCHLETPRAVALAALWNKPVLRPTYGRLTDVYAYAKQPLKTGDLFADGIGGD----GAYGLIHE : 352
Coxiella_burnetii_CbuK_Q154                    : KCEDRLQESYLNYY--KVHNRYPYYVFYRPYHLCHLETPRAVALAALWNKPVLRPTYGRLTDVYAYAKQPLKTGDLFADGIGGD----GAYGLIHE : 352
Coxiella_burnetii_Dugway_5J108-111             : KCEDRLQESYLNYY--KVHNRYPYYVFYRPYHLCHLETPRAVALAALWNKPVLRPTYGRLTDVYAYAKQPLKTGDLFADGIGGD----GAYGLIHE : 352
Coxiella_burnetii_RSA_331                      : KCEDRLQESYLNYY--KVHNRYPYYVFYRPYHLCHLETPRAVALAALWNKPVLRPTYGRLTDVYAYAKQPLKTGDLFADGIGGD----GAYGLIHE : 352
Coxiella_burnetii_RSA_493                      : KCEDRLQESYLNYY--KVHNRYPYYVFYRPYHLCHLETPRAVALAALWNKPVLRPTYGRLTDVYAYAKQPLKTGDLFADGIGGD----GAYGLIHE : 352
Cyanothece_sp._PCC_7425                        : SHDDPKQRHYLNLY---KLGEGPLYSFYTPYHLCHFEVPLSVARVVLFNDPVLTPQAGPQVDVVATAKIDLKAGETL-DGMGYY----MTYGQCEN : 373
Cyclobacterium_marinum_DSM_745                 : TIDDPIQKHYLNLY---KVGEGPLYCFYNPYHLCHFEVPITVARAVLFQDASIAP-EKPMVDVVTTAKRDLKKGEKI-DGIGHY----MTYGLCEN : 374
Deinococcus_maricopensis_DSM_21211             : THDDPKQQHYLNLY---KLGEGPLYSFYTPYHLCHFEVPLSAARAVLFRDAVLQPLGAPVVDVVTTAKRDLKAGEVI-DGLGGY----MTYGQCEN : 373
Desulfococcus_oleovorans_Hxd3                  : HCDNPYQREMLAYY---KMGPGPFYLFYRPYHLCHIEAMGTVLQAARRQTPFLVPDYGFQTQVYAYAKRDLKAGEVL-DGIGGY----CCYGLIEN : 365
Frankia_sp._EuI1c                              : TFEEE-LGEFLRYY-KVRLGDSPYVLLERPFHLCNLEVPLTVDRLLRGDPVAFNNGPRPRVGVAAVAKRPLAAGERIETAIGGF----EVRG--EA : 352
Gloeobacter_violaceus_PCC_7421                 : GCDDPTQRHYLNLY---KLGEGPLYSFYTPYHLCHFEVPFSVARAVLFADAVLAPLGAPVVEVVATAKRPLEAGETL-DGIGHY----LSYGQCEN : 373
Hirschia_baltica_ATCC_49814                    : WQEDPIQQHYYNYY---KLGKGPLYVFYDPYHLCHMQVPISIGRVVLFNDAVISPDGAPKVGVVAIAKKDLSEGDTI-IDMGGY----EVYGETEN : 380
Kineococcus_radiotolerans_SRS30216             : THDDPKQQHYLNLY---KLGTGPLYSFYTPYHLCHFEVPLTVARAVLFADAAIVPGDEVKVEVVTTAKTDLAAGKEI-DRLGGY----DSYGVAET : 373
Methanosphaerula_palustris_E1-9c               : EHEE-RLIPYLRYY---KMGEGPWYTLVRNFHLCHLEVIKTIRRVLDGGEVLLNNSTAPTVSVGAIAKRDLEPGQILKRGIGSF----DVRGTALT : 329
Nakamurella_multipartita_DSM_44233             : TIDDPVQAHYLNLY---KLGAGPLYSFYTPYHLCHFEVPASLARAVDFGDAAIAAAGRPMVEVVATAKTDLSPGQVI-DDLGGY----LTYGEAER : 373
Nostoc_azollae_0708                            : THDDPKQRHYLNLY---KLGEGPLYSFYTPYHLCHFEVPLSVARAVLFHDAVLSPIAGPIIDVVATAKIDLKAGETL-DGIGYY----MTYGQCEN : 373
Nostoc_punctiforme_PCC_73102                   : THDDPKQQHYLNLY---KLGEGPLYSFYTPYHLCHFEVPLSVARAVLFGDAVMSPLAGPLVDVVTTAKIDLKAGETL-DGIGYY----MTYGQCEN : 373
Nostoc_sp._PCC_7120                            : THDDPKQRHYLNLY---KLGEGPLYSFYTPYHLCHFEVPLSVARAVLFSDAVMAPLAGPSVDVITTAKIDLKAGETL-DGIGYY----MTYGQCEN : 373
Pseudomonas_stutzeri_A1501                     : RHDEDQARA-IEYF---KLGRGPFYTLVRPFHLCSLEVGKTVRRVLSGGGVLLNNSTEPTLGVAAIAKRAMKPGELIERGIGGF----QFRG--EA : 316
Pseudomonas_stutzeri_ATCC_17588__LMG_111       : RHDEDQARA-IEYF---KLGRGPFYTLVRPFHLCSLEVGKTVRRVLSGGGVLLNNSTEPTLGVAAIAKRAMKPGELIERGIGGF----QFRG--EA : 316
Pseudoxanthomonas_spadix_BD-a59                : THDDPKQRHYLNLY---KLGEGPLYSFYTPYHLCHFEVPLSVARVVLLRDAVLQPLDGPLVEVVATAKRDLEAGETL-DALGGY----LTYGQCER : 371
Ramlibacter_tataouinensis_TTB310               : THDHPQQRHYLNLY---KLGEGPLYCFYTPYHLCHFEVPNTVARAVLFQDAALAPLAGPLVEVVATAKTDLKAGDTL-DGLGGY----LTYGLAEN : 375
Rhodobacter_sphaeroides_2.4.1                  : THDDPRQKHYLNLY---KLGEGPYYLFYTPYHLCHFEVPMTVARAVLFGDAALAPLGAPQVGVVAVAKRDLKAGEVL-DGIGGF----TAYGQCEN : 382
Rhodobacter_sphaeroides_ATCC_17025             : THDDPRQKHYLNLY---KLGEGPYYLFYTPYHLCHFEVPMTVARAVLFGDAALTPLGAPQVGVVAVAKRDLKAGEVL-DGIGGF----TAYGQCEN : 382
Rhodobacter_sphaeroides_ATCC_17029             : THDDPRQKHYLNLY---KLGEGPYYLFYTPYHLCHFEVPMTVARAVLFGDAALAPLGAPQVGVVAVAKRDLKAGEVL-DGIGGF----TAYGQCEN : 382
Rhodobacter_sphaeroides_KD131                  : THDDPRQKHYLNLY---KLGEGPYYLFYTPYHLCHFEVPMTVARAVLFGDAALAPLGAPQVGVVAVAKRDLKAGEVL-DGIGGF----TAYGQCEN : 382
Rhodothermus_marinus_DSM_4252                  : EHDD-YQRDYLQYL---KLGDGPYYVLLRPFHLCHLEIQKTIRRVLFERRILLNNTSHPRISVATIAKRRLPKGTRIERGIGSF----EVRG--EA : 327
Rhodothermus_marinus_SG0.5JP17-172             : EHDD-YQRDYLQYL---KLGDGPYYVLLRPFHLCHLEIQKTIRRVLFERRILLNNTTHPRISVATIAKRRLPKGTRIERGIGSF----DVRG--EA : 331
Sphingomonas_wittichii_RW1                     : TQDDPRQRHFLNLY---KLGTGPYYCFSTPYHLCHFEVPTSIARAALFGDAVLAPLGAPRVGVIAVAKKALRPGDVI-GEFGGF----EAYGVAEN : 380
Streptomyces_griseus_subsp._griseus_NBRC       : THDDPAQHPYLELY---KLGPGPLYSFYTPYHLCHFEVPNTVVRAVDFGDAALTPPAGPHVDVVATAKRDLPAGTTL-DGIGGY----DSYGVAES : 372
Xanthomonas_campestris_pv._campestris_st       : THDDPKQRHYLNLY---KLGEGPLYSFYTPYHLCHFEVPLSVSRVVLLGDAVLQPLGAPLVDVIATAKRDLAAGETI-DGLGGY----MSYGQCER : 371
Xanthomonas_campestris_pv._campestris_st       : THDDPKQRHYLNLY---KLGEGPLYSFYTPYHLCHFEVPLSVSRVVLLGDAVLQPLGAPLVDVIATAKRDLAAGETI-DGLGGY----MSYGQCER : 371
Xanthomonas_campestris_pv._campestris_st       : THDDPKQRHYLNLY---KLGEGPLYSFYTPYHLCHFEVPLSVARVVLLGDAVLQPLGAPLVEVIATAKRDLAAGETI-DGLGGY----MSYGQCER : 371
Achromobacter_xylosoxidans_A8                  : AADSDYVRRCFKEYGLVTDPSGNYTAMYKPYHLIGLELGISVASVGLRREPTGAP-AGWHGDVVATAKRDLPAGQIL-DGEGGY----TVYGRLMP : 390
Acidiphilium_cryptum_JF-5                      : EAPNDYAAACFAQYGLKTDASGRYAAMYKPYHLIGLELNVSVLSVALRGEPTGRP-RGFAGDVVAIAKRDLAAGEVL-DGEGGY----TVWGKLLP : 379
Acidiphilium_multivorum                        : EAPNDYAAACFAQYGLKTDASGRYAAMYKPYHLIGLELNVSVLSVALRGEPTGRP-RGFAGDVVAIAKRDLAAGEVL-DGEGGY----TVWGKLLP : 379
Acidovorax_ebreus_TPSY                         : EGETDYIRHCFEEYNAHTDPTGRYFTLYKRWHLIGLEVGMSVASVALRGEPTGVP-QGWNADVVATAKRDLRPGDLL-DGEGGY----TVWGKLLP : 390
Acidovorax_sp._JS42                            : EGETDYIRHCFEEYNAHTDPTGRYFTLYKRWHLIGLEVGMSVASVALRGEPTGVP-QGWNADVVATAKRDLRPGDLL-DGEGGY----TVWGKLLP : 390
Actinosynnema_mirum_DSM_43827                  : RTGDPVTWELLRGKGHVVSRDGRYACLYWPYHAMGVETPLTVHAAVDGTGPAHAP--SPSVLLAARTATALDAGTAF-RVAGHH----HEIDGVTP : 377
Agrobacterium_sp._H13-3                        : KAHNEYARACFKQYGLLTDPSGWYASMWRPFHIIGLETSISVLSAVLRNEATGCS-KEFRGDAVATAKKDMQPGEML-DGEGGY----AVWANAIP : 390
Agrobacterium_tumefaciens_str._C58             : KAHNDYARACFKQYGLLTDPSGWYASMWRPFHIIGLETSISVLSAVLRNEPTGCS-KEFRGDAVATAKKDMQPGEML-DGEGGY----AVWANAIP : 427
Anaeromyxobacter_sp._Fw109-5                   : EAESPYVERCFREYGLVTDESGRYTATYKPFHLIGLELGISVASVALRREPTGQP-RRWLADVMAVAKRDLAAGEVL-DGEGGY----LVFGKLAP : 390
Arthrobacter_sp._FB24                          : KATNAYVSGCFTEYPWHPDPTGQYAALYRPYHYVGLELNMSIANAALRGISTGSP-IGFFGDVVATAKKNLKAGEFL-DGEGGY----TVWGQLVS : 388
Azorhizobium_caulinodans_ORS_571               : KATSEYTKACFKQYGLLTDPSGWYGSMWRPFHMIGLETSISVLSAVLRHEPTGCS-KEFRGDAVATAKRDLKPGEML-DGEGGY----AVWANAIP : 390
Bordetella_avium_197N                          : AADSDFVRSSFRDFGLVTDPSGNYAAMYRPYHLMGMELGMSVASIGLRREPTGMP-TAWHADVIATAKRDMAAGEVL-DGAGGY----SVYGRLVP : 390
Bordetella_bronchiseptica_RB50                 : AGDSDYVRRCFKEYGLVTDPSGNYSAMYKPYHLIGLELGISVASVGLRREPTGSP-AGWHGDVVATAKRDMAAGQEL-DGEGGY----TVYGRLMP : 390
Bordetella_parapertussis_12822                 : AGDSDYVRRCFKEYGLVTDPSGNYSAMYKPYHLIGLELGISVASVGLRREPTGSP-AGWHGDVVATAKRDMAAGQEL-DGKGGY----TVYGRLMP : 390
Bordetella_petrii_DSM_12804                    : AADSDYVRRCFKEYGLITDPTGNYAAMYKPYHLIGLELGISVASVGLRREPTGSP-SGWHGDVVATAKRDLPAGQEL-DGEGGY----TVYGKLMP : 390
Bradyrhizobium_japonicum_USDA_110              : EAPNDYAADCFKQYGLKTDSSGRFAAMYKPYHLIGLELNISVLSAALRGEPTGQP-HGFRGDVAAVAKRALRAGEML-DGEGGY----TVWGKLVP : 382
Bradyrhizobium_sp._BTAi1                       : EAPNDYAADCFRQYGLKTDTSGRYAAMYKPYHLIGLELNVSVLSAALRKEPTGQP-RGFRGDVVAVAKKTLRAGEML-DGEGGY----TVWGKVFP : 378
Bradyrhizobium_sp._ORS278                      : EAPNDYAADCFKQYGLKTDATGRYAAMYKPYHLIGLELNVSVLSAALRSEPTGQP-LCFRGDVVAVAKKDLRAGEIL-DGEGGY----TVWGKVMP : 384
Candidatus_Pelagibacter_ubique_HTCC1062        : KAQNQYVKNCFKDYGMVTDLSGSYSAIWRPYHYIGLELAQSIYAIALDQKATGQT-INYNADVAAYAKKDLKIGERL-DGEGGF----CARGKLIT : 376
Cupriavidus_necator_N-1                        : EAPTEYIRRCFKEYQVFTDPNGQCMVQYKKWHLIGLELGISVASVGLRREPTGVG-QHFNADVVATAKRDLKAGEML-DGEGGY----TVFGKLFP : 390
Cupriavidus_taiwanensis                        : EAPTDYIRRCFREYQVFTDPDGRCMVQYKKWHLIGLELGISVASVGLRREPTGAG-RHFNADVVATAKRDLKAGEML-DGEGGY----TVFGKLFP : 390
Methylibium_petroleiphilum_PM1                 : AADSEYVRRCFKEYGLVTDPSGEYSSMYKPFHLIGLELGISVASAGLRREATGAA-TGWRGDVVATAKRALRAGEVL-DGEGGY----TVYGKLMP : 390
Methylobacterium_nodulans_ORS_2060             : KATSEYTRACFRQYGLLTDPSGWYGSMWRPFHMIGLETSVSVLSAVLRNEPTGSS-KEFRGDAVATAKRDLKPGEML-DGEGGY----AVWANAIP : 390
Methylobacterium_radiotolerans_JCM_2831        : EAPSPYVRDCFAQYGLKTDSSGRFAATYKPYHLIGLELGISVASIAVRGEATGAT-GEWRGDVAATAKRALRAGERL-DGEGGF----TVYGKLMP : 390
Methylobacterium_sp._4-46                      : KATSEYTRACFRQYGLLTDPSGWYGSMWRPFHMIGLETSVSVLSAVLRQEPTGCS-KEFRGDAVATAKRDLKAGEML-DGEGGY----AVWANAIP : 390
Mycobacterium_smegmatis_str._MC2_155           : EAVTDYAVQCFAEYGVHTDETGRYGSLYRPYHMIGLELAVSIASAVLRNEATGSP-TGFRGDVMTTAKRDLKAGEKL-DGEGGF----TVFGKLAP : 391
Nocardiopsis_dassonvillei_subsp._dassonv       : RTGDPVSWALLAQKGHVVSRSGRYACLYLPYHLMGVETPLSLLDAVDRRRAVTPRHPAPHAVLAGRARRDLPAGTRL-DMGGHHHDVQGVAPVLLD : 404
Paracoccus_denitrificans_PD1222                : RCDDAETWKMLEGKGHVVARDRNTAMIYNPRHLLGLEAATTILSLGLLARGTGGHAPAHHIDLVAFADRDLAAGEAL-VMGGHHHSIPGVSARMVA : 405
Polaromonas_sp._JS666                          : EAETDYIKNCFEEYNAHTDPSGRYFTLYKRWHLIGLEVGVSVASVALRGEPTGVA-TCWNADVVATAKRDLKPGDML-DGEGGY----TVWGKLLP : 390
Polymorphum_gilvum_SL003B-26A1                 : EAQTDYARQCFTEYHMLEDDSGRFAALYRPTHMIGMELGVSVASAALRREPTGAP-VGFHSDVVATAKKPMKAGDML-DGEGGF----TVWGKQCP : 390
Polynucleobacter_necessarius_subsp._asym       : EAPSQYVIDCFSQYGLKTDSTGKYAAMYKPYHLIGLELGISVASIAVRGEATGAT-GDWRGDVVATTKRALKAGEKL-DGEGGF----TVYGKLMT : 390
Pusillimonas_sp._T7-7                          : EAETDYIKNCFEEYNAHTDPSGRYFTLYKRWHLIGLEVGMSVASVALRQEATGVA-TCWQADVVATAKRDLQPGDIL-DGEGGY----TVWGKLLP : 390
Rhodopseudomonas_palustris_BisB5               : EAPNDYAADCFKQYGLKTDSSGRYAAMYKPYHLIGLELGISVLSAALRREPTGQP-RDFRGDVVAVAKRDLKAGEML-DGEGGY----TVWGKLMR : 378
Rhodospirillum_rubrum_ATCC_11170               : RVKDADSWEVLRQKGHVVSRNGKYACLYLPYHIMGLETPISLFSAVLHGRPSGGMKIVRHAVMAMRTDRAFRAGERL-AMGGHHHTVADVTALLLP : 406
Spirochaeta_smaragdinae_DSM_11293              : ACDDEETWQVLQDKGVPVSRNKKTALLYYPAHYLGFEALFSVLSVGLLGLPTSSPDPRPRYDLVARSVKPMTKGTEL-QAKGHHHIIEGFEGILLP : 406
Spirochaeta_sp._Buddy                          : RCHDEKVWGLLKAKGHIVSQNGKYACMYLPYHFMGVEAPLSVVNAFRLGLSTYDA-CTHQSIMLATAERDFKQGERM-DLRGHHRLLDDVYVELLS : 406
Streptomyces_flavogriseus_ATCC_33331           : RTGDPVTWETLRGKGHVVSRDGRYAAVYLPYHLMGVETPVSLLSAVLHGRPSGGTDPQAHAVLAGRAERDLPAGTVL-DMGGHHHDVTGVQAVLLR : 406
Streptomyces_sp._SirexAA-E                     : RTGDPVTWETLRGKGHVVSRDGRYAAIYLPYHLMGVETPVSLLSAVLHGRPSGGTDPRAHAVLAGRARRDLPAGTVL-EMGGHHHDVTGVQAVLLR : 406
Variovorax_paradoxus_EPS                       : EAETDYIKNCFEEYNAHTDPSGRYFTLYKRWHLIGLEVGMSVASVALRGEATGVA-TCWNADVVATAKRDLAAGEML-DGEGGY----TVWGKLLP : 390
Variovorax_paradoxus_S110                      : EAETDYIKNCFEEYNAHTDPSGRYFTLYKRWHLIGLEVGMSVASVALRGEPTGVA-TCWNADVVATAKRDLAPGEML-DGEGGY----TVWGKLLP : 390
Xanthobacter_autotrophicus_Py2                 : EAPSDYVKQCFAQYGLKTDSTGRYATMYKPYHLIGLELGISIASAALRGEATGST-QGFRGDVVATAKRDLKPGEKL-DGEGGF----MVYGKLMP : 390
```

```
                                                      *       500         *       520         *       540         *       560         *
Anaerococcus_prevotii_DSM_20548               : HDRSLAEDLLPIAL------ITDKTKAVKDIEKGTVIDMSMVELDEK----ATITRLRRRQNS-MK-----L---------------------- : 430
Bacillus_clausii_KSM-K16                      : HQDMKRNGHIPIGL------ISGNVVAKRAIEEGKFLTMDDVELDTM----TTVWKERKLQDH-LF-----AE--------------------- : 418
Bacillus_halodurans_C-125                     : VTDAKEKGALPIGL------VDAHVQVKRPIKKGTLITYDDVEQKKE----STIWRLRIQDETLV-----G--KRELEAKV------------ : 439
Bacillus_pumilus_SAFR-032                     : HQDMKTNGHIPIGL------ISGKVVAKKDIKLGKFLTHDDVELDSN----TTVWKERKLQDH-LF-----QS--------------------- : 414
Bartonella_bacilliformis_KC583                : VAEARSQQAIPCGL------LEKATVT-AEIKKNEFITMHNTAIREN----QWIANERTKQDL-LL------N--LHAS--------------- : 441
Bartonella_clarridgeiae_73                    : AEEARAHQAIPCGL------LEKAIVT-TAIKKNEFITKHNTTIREN----QWIARERNKQDL-LL------R---------------------- : 437
Bartonella_grahamii_as4aup                    : IAEARAHHAIPCGL------LEKATVT-AAIKKNEFITIHNTAIRED----QWIARERAKQDL-LL------H--NPSSDKL------------ : 444
Bartonella_henselae_str._Houston-1            : IAEARMHQAIPCGL------LENATVT-AEIKKNEFITVHNTAIRED----QWIARERAKQDL-LL------N--ASSLPSHA----------- : 445
Bartonella_quintana_str._Toulouse             : IAEARAHQAIPCGL------LEKATVT-AEIKKNEFITVHNTAIREN----QWIARERNKQDL-LL------N--CPPPFAHA----------- : 445
Bartonella_tribocorum_CIP_105476              : IAEARAHHAIPCGL------LEKATVT-AAIKKNEFITVHNTAIRED----QWIARERAKQDL-LL------H--CPSLG-------------- : 442
Brucella_abortus_bv._1_str._9-941             : VPEARAAKAIPCGL------LQNGTVI-APIKKGELITYANAAPQPG----SRIAEERALQDA-ML------G--Q------------------ : 438
Brucella_abortus_S19                          : VPEARAAKAIPCGL------LQNGTVI-APIKKGELITYANAAPQPG----SRIAEERALQDA-ML------G--Q------------------ : 438
Brucella_canis_ATCC_23365                     : VPEARAAKAIPCGL------LQNGTVI-APIKKGELITYANAAPQPG----SRIAEERALQDA-ML------G--Q------------------ : 438
Brucella_melitensis_ATCC_23457                : VPEARAAKAIPCGL------LQNGTVI-APIKKGELITYANAAPQPG----SRIAEERALQDA-ML------G--Q------------------ : 438
Brucella_melitensis_biovar_Abortus_2308       : VPEARAAKAIPCGL------LQNGTVI-APIKKGELITYANAAPQPG----SRIAEERALQDA-ML------G--Q------------------ : 438
Brucella_melitensis_bv._1_str._16M            : VPEARAAKAIPCGL------LQNGTVI-APIKKGELITYANAAPQPG----SRIAEERALQDA-ML------G--Q------------------ : 434
Brucella_microti_CCM_4915                     : VPEARAAKAIPCGL------LQNGTVI-APIKKGELITYANAAPQPG----SRIAEERALQDA-ML------G--Q------------------ : 438
Brucella_ovis_ATCC_25840                      : VPEARAEKAIPCGL------LQNGTVI-APIKKGELITYANAAPQPG----SRIAEERALQDA-ML------G--Q------------------ : 438
Brucella_pinnipedialis_B2/94                  : VPEARAAKAIPCGL------LQNGTVI-APIKKGELITYANAAPQPG----SRIAEERALQDA-ML------G--Q------------------ : 438
Brucella_suis_1330                            : VPEARAAKAIPCGL------LQNGTVI-APIKKGELITYANAAPQPG----SRIAEERALQDA-ML------G--Q------------------ : 438
Brucella_suis_ATCC_23445                      : VPEARAAKAIPCGL------LQNGTVI-APIKKGELITYANAAPQPG----SRIAEERALQDA-ML------G--Q------------------ : 438
Chelativorans_sp._BNC1                        : AEEARSAKAIPCGL------LQGGTVL-KPVRKGEIITYDNAAPPPD----SKIAEERAIQDR-LI----NKT--------------------- : 439
Chloroflexus_aurantiacus_J-10-fl              : FEKAHRDGEVPLGL------IVGATVI-RPIKAHCPIHYDEVELNKS----QVIFQLRQEQDQ-LL------G--C------------------ : 434
Chloroflexus_sp._Y-400-fl                     : FEKAHRDGEVPLGL------IVGATVI-RPIKAHCPIHYDEVELNKS----QVIFQLRQEQDQ-LL------G--C------------------ : 434
Clostridium_difficile_630                     : YDVAKAMNAVPIGL------ISKKTKVVKDIKKGEVITYDMVEIEKD----TTLYHLRQLQEK-IF------G---------------------- : 430
Clostridium_difficile_CD196                   : YDVAKAMNAVPIGL------ISKKTKVVKDIKKGEVITYDMVEIEKD----TTLYHLRQLQEK-IF------G---------------------- : 430
Clostridium_difficile_R20291                  : YDVAKAMNAVPIGL------ISKKTKVVKDIKKGEVITYDMVEIEKD----TTLYHLRQLQEK-IF------G---------------------- : 430
Eubacterium_limosum_KIST612                   : ASEADRQNALPMGL------VDKKTQLVHDKKAGEVITYDDVVLNND----NLIVQLRKLQDE-LF------I---------------------- : 433
Finegoldia_magna_ATCC_29328                   : HKHQMENNLLPMPI------ITEKTTAKVDIPKDTLITYDMVNLDED----HIITKLRKRQDE-LG------L---------------------- : 430
Halanaerobium_hydrogeniformans                : AEVAREKNLLPLGL------SEGATLK-EDVKQGKKLTLDMVDLNKD----SVIYELWKLQKR-VF------N---------------------- : 430
Listeria_innocua_Clip11262                    : HVDMAAEGHIPIGL------ISGEVVARRNIKAGTFITDEDVSLDES----TTVWKLRKLQDE-TF-----SN--------------------- : 416
Listeria_ivanovii                             : HTDMATNGHIPIGL------ISGEVVARRNIKAGTFITTEEDVTLDES----TTVWKLRKLQDE-TF-----SN--------------------- : 416
Listeria_monocytogenes                        : HVDMATNGHIPIGL------ISGEVVARRNIKAGTFITDEDVSLDES----TTVWKLRKLQDE-TF-----SK--------------------- : 416
Listeria_monocytogenes_08-5923                : HVDMATNGHIPIGL------ISGEVVARRNIKAGTFITDEDVSLDES----TTVWKLRKLQDE-TF-----SN--------------------- : 416
Listeria_monocytogenes_EGD-e                  : HVDMATNGHIPIGL------ISGEVVARRNIKAGTFITDEDVSLDES----TTVWKLRKLQDE-TF-----SK--------------------- : 416
Listeria_monocytogenes_HCC23                  : HVDMATNGHIPIGL------ISGEVVARRNIKAGTFITDEDVSLDES----TTVWKLRKLQDE-TF-----SK--------------------- : 416
Listeria_monocytogenes_serotype_4b_str._      : HVDMATNGHIPIGL------ISGEVVARRNIKAGTFITDEDVSLDES----TTVWKLRKLQDE-TF-----NK--------------------- : 416
Listeria_monocytogenes_serotype_4b_str._      : HVDMATNGHIPIGL------ISGEVVARRNIKAGTFITDEDVSLDES----TTVWKLRKLQDE-TF-----NK--------------------- : 416
Listeria_welshimeri_serovar_6b_str._SLCC      : HLDMKTNGHIPIGL------ISGEVVARRNIKAGTFITDEDVSLDES----TTVWKLRKLQDE-TF-----NN--------------------- : 416
Mesorhizobium_ciceri_biovar_biserrulae_W      : TPEARAAKAIPCGL------LQGGSVT-APIKKGELITYANAAPAAG----SKIAEERARQDK-LV------Y--GTVGA-------------- : 442
Mesorhizobium_loti_MAFF303099_1               : APEAHAAKAIPCGL------LQGGSVT-APIKKGELITYANAVPAPG----SKIAEERARQDK-LV------Y--GTVGA-------------- : 442
Mesorhizobium_loti_MAFF303099_2               : VGEARAQHAVPVGL------LEGGKVL-KPVRKGELIADNAAPDQT----TRLFAERLQDE-ML------Y--GAN---------------- : 433
Mesorhizobium_opportunistum_WSM2075           : APEAHAARAIPCGL------LQGGSVT-APIKKGELITYANAAPAPG----SKIAEERARQDK-LV------Y--GTVGA-------------- : 442
Ochrobactrum_anthropi_ATCC_49188_1            : TPEAHAAKAIPCGL------LQNGTVI-APIRKGELITYANAAPQPG----SKIAEERARQDK-MI------Y--G------------------ : 438
Ochrobactrum_anthropi_ATCC_49188_2            : SGEARDAHAIPCGL------LQGGSVT-KPIKKGELITYDNAAVAPG----SKIAEERARQDK-LV------Y--GA----------------- : 439
Pelagibacterium_halotolerans_B2               : IADARTARAIPVGL------LEGGRVT-APVKKGELITAANATPDIS----TKLFAERQRQDA-MF------D--LGPLAVGA----------- : 446
Roseobacter_litoralis_Och_149                 : TSRARTARALPAGL------LTGAVTT-AAIAKGELITAHNTTL-PD----ARIVEERQRQDE-MI------Y--GKDAANV------------ : 443
Sebaldella_termitidis_ATCC_33386              : YETAKEMNALPVGL------ITKKTKLVKDVKKGEVITYDMVELDES----SFILQLRQVQDK-LF------G---------------------- : 430
Sinorhizobium_fredii_NGR234                   : APEARSSSAIPCGL------LQGGTIT-RPILKGELITYQNASVAPG----SRIAEERARQDA-LV------Y--GREA--------------- : 441
Starkeya_novella_DSM_506                      : WRDARNSFALPLGL------AERSRVL-KPIKAGERITYDNCAPDEQ----LLVTQLRRRLDQ---------A--DTQYLAA------------ : 444
Tepidanaerobacter_sp._Re1                     : AENARKQNALPIGL------INDKTVLKTDVKKGEVICYDMVNLDDD----SIVLQLRRIQDK-FF-----KN--------------------- : 431
Thermosediminibacter_oceani_DSM_16646         : YENAKRENALPIGL------VNNNVVMTKDVKKGEVITYDAVKLDEN----SLILQLRMQEK-LI------G---------------------- : 430
Verminephrobacter_eiseniae_EF01-2             : VADARRQRAVPCGL------LEQGTVL-QPIRKGELITYSAVDAG----SRIVEERADQDR-LI------H--GKQGAAA------------ : 443
Anabaena_variabilis_ATCC_29413                : SDIVQQQNLLPMGL------AEGCRLK-RDIPKDEVLTYDDVELPEG----RLCDQLRAEQDK-YF------APEKVLVTVG------------ : 437
Chloroflexus_aggregans_DSM_9485               : ADVVYAERLLPIGL------AEGCTLR-RDIPKDGIITYDDVELPTD----RLSDRLRAEQDA-LF------W--GKPATAGASEGQYQRIAHTE : 453
Coraliomargarita_akajimensis_DSM_45221        : HVAVQSEKLLPIAF------SKGKTLL-RDIPKDSAVTVADVAEKPS----TAVELAYQSICS-NS------------------------------ : 427
Coxiella_burnetii_CbuG_Q212                   : AESADQANYLPIGLLEAEETHEKPRVL-HDLERDQPIRWSDVELPDT----EL-LRLYRIQQK-LL------K--TEVVTV------------- : 418
Coxiella_burnetii_CbuK_Q154                   : AESADQANYLPIGLLEAEETHEKPRVL-HDLERDQPIRWSDVELPDT----EL-LRLYRIQQK-LL------K--TEVVTV------------- : 418
Coxiella_burnetii_Dugway_5J108-111            : AESADQANYLPIGLLEAEETHEKPRVL-HDLERDQPIRWSDVELPDT----EL-LRLYRIQQK-LL------K--TEVVTV------------- : 418
Coxiella_burnetii_RSA_331                     : AESADQANYLPIGLLEAEETHEKPRVL-HDLERDQPIRWYDVELPDT----EL-LRLYRIQQK-LL------K--TEVVTV------------- : 418
Coxiella_burnetii_RSA_493                     : AESADQANYLPIGLLEAEETHEKPRVL-HDLERDQPIRWYDVELPDT----EL-LRLYRIQQK-LL------K--TEVVTV------------- : 418
Cyanothece_sp._PCC_7425                       : AAVVRSQRLLPMGL------AEGCRLK-RSIPRDEVLTYDDVELPPG----RLSDQLRAEQDA-YF------A--SPKSLATVV---------- : 437
Cyclobacterium_marinum_DSM_745                : MSTSLEEGLLPIGL------AVDCVLR-RDIPKDEVITYEDVILPEN----RFSDQLRAEQNK-HF------F----DL--------------- : 431
Deinococcus_maricopensis_DSM_21211            : ADVTRAERLLPMGL------AEGCRVL-RDIPKDTVLTYADVDLPAG----RLAAQLRAQQDR-HF------A--PVEVL---G---------- : 434
Desulfococcus_oleovorans_Hxd3                 : FKENHASPGLPIGL------ADNVALR-RDVPEQGRIISLDDVSYDPA----RLDFALFDRAFG-LP------A--NAAV--------------- : 424
Frankia_sp._EuI1c                             : VLIADEPDHVPIGL------LAGATVE-RPVAAGEPVRFADVSVPES----LALRAWRWTLDAACS------T--PGVAGQGRAASLPLPQGVRP : 428
Gloeobacter_violaceus_PCC_7421                : AEAAAAGRLLPMGL------AEGCRLR-RDIAQDEVLCYDDVVLPPG----RLCDRLRAEQDA-CF------Q--RSAVPLTAC---------- : 437
Hirschia_baltica_ATCC_49814                   : QTTINEENLLPIGI------AMDCKLK-RAVAKDEAITFEDVELPEG----RIIDAIYQEQIA-YF------SN--------------------- : 436
Kineococcus_radiotolerans_SRS30216            : YAITKAQNLLPMGV------AEGAVLK-HAVKKDEVLTYDDVVLPQG----RLIDALRAEQAA-LL------A-----H----------------- : 429
Methanosphaerula_palustris_E1-9c              : I--ANNSQHVPIAL------LTGAVLT-EQVKAGAQITFDDVEVPAS----LACDIWLKNN------------------------------- : 377
Nakamurella_multipartita_DSM_44233            : ADVTAAQRLLPMGV------AAGCTVV-RPVAKDATLTYDDVAVPQG----RLIDKLRDEQTA-VF------G---------------------- : 428
Nostoc_azollae_0708                           : SEIVHQENLLPMGI------AEGCTLK-RDISKDKVLTYDCVELPEG----RLCDKLRAEQLT-YF------V--PVKTLVLAK---------- : 437
Nostoc_punctiforme_PCC_73102                  : YPIVQQQNLLPMGL------AEGCRLK-RDISKDEVLTYEDVELPEG----RLCDQLRTEQNN-YF------A--PEKILVAVG---------- : 437
Nostoc_sp._PCC_7120                           : SDIVQQQNLLPMGL------AEGCRLK-RDVPKDEVLTYDDVELPEG----RLCDQLRAEQDK-YF------A--PEKVLVTVG---------- : 437
Pseudomonas_stutzeri_A1501                    : IKLAQHPDHVPIGL------LRKTALK-RAVEPGEIITFDDIDILPSRALDIVMEQRKPRLVE-VE------S--KSEGWTAPCINTMGMLALGG : 395
Pseudomonas_stutzeri_ATCC_17588__LMG_111      : IKLAQHPDHVPIGL------LRKTALK-RAVEPGEIITFDDIDILPSRALDIVMEQRKPRLVE-VE------S--KSEGRTAPCINTMGMLALGG : 395
Pseudoxanthomonas_spadix_BD-a59               : ADVTAREALLPMGV------AEGCVLK-RAIARDAVITYDDVTLPHG----RLIDRLRAEQAA-LF------A----TTADAVAA--------- : 434
Ramlibacter_tataouinensis_TTB310              : APLAREQDLLPLGL------AEGCTLR-RDVAKDAVITFADVTLPAG----RLCDRLWAEQMR-RF------W--PRHSTTTQGPTA-------- : 442
Rhodobacter_sphaeroides_2.4.1                 : METFVSEQLLPMGL------AEGGVLL-RDIPRDQALTFADVRLPAG----RRIDALYEEMER-EF------G--LARPAEAHALTV-------- : 449
Rhodobacter_sphaeroides_ATCC_17025            : IEAVAAERLLPMGL------AEGGVLL-RDIPRDETLTFADVRLPEG----RRIDALYEEMEQ-EF------G--LAQPAETFALIG-------- : 449
Rhodobacter_sphaeroides_ATCC_17029            : METFVSEQLLPMGL------AEGGVLL-RDIPRDQALTFADVRLPVG----RRIDALYEEMER-EF------G--LARPAEAHALTV-------- : 449
Rhodobacter_sphaeroides_KD131                 : METFVSEQLLPMGL------AEGGVLL-RDIPRDQALTFADVRLPAG----RRIDALYEEMER-EF------G--LLRPAEARAVTV-------- : 449
Rhodothermus_marinus_DSM_4252                 : VRILDEPDHVPIGL------MADVVLT-RDVEPGEILRFDDVELPDT----LALRAWRWQAILERVRLKKAVEEQ--PAPTASR---------- : 396
Rhodothermus_marinus_SG0.5JP17-172            : VRILDEPDHVPIGL------MVDAVLT-RDVEPGEILRFEDVELPDT----LALRAWQAILERVRLKKAVEEQ--PAPTASR---------- : 400
Sphingomonas_wittichii_RW1                    : ADVIDRERLLPIGL------ALGCTLV-RPVAEDAPIGRDDVFVPAG----RLIDRLYAEQQE-HF------A--GKRQAA------------ : 441
Streptomyces_griseus_subsp._griseus_NBRC      : HAVTRADNLLPMGI------SEGCVLR-RAVAKDSVLGYADVTLPPG----RLVDVLRAEQDK-QF-----------PV--------------- : 428
Xanthomonas_campestris_pv._campestris_st      : ADVTARARLLPMGV------AEGCVLR-HAVTKDEVLTYDDVQLPQH----RLIDQLRAEQAA-VF------A--SAELAA------------ : 432
Xanthomonas_campestris_pv._campestris_st      : ADVTAREALLPMGV------AEGCVLR-HAVTKDEVLTYDDVQLPQH----RLIDQLRAEQAA-VF------A--SAELAA------------ : 432
Xanthomonas_campestris_pv._campestris_st      : ADVTARERLLPMGV------AEGCVLR-HAVTKDEVLTYDDVQLPQH----RLIDQLRAEQAA-VF------A--PAGLAA------------ : 432
Achromobacter_xylosoxidans_A8                 : ARDSVDEGYLPIGL------SHQVKLK-NPVRQSQAIRWSDVEYDNS----STAVQFRREMEQ-TF------A---------------------- : 445
Acidiphilium_cryptum_JF-5                     : AQASLAAGALPIGL------AHHARLR-RGIAEGEVVGWDDVDFAAGSALQTAIAARREMER-RF------A--APRAEAGAA----------- : 447
Acidiphilium_multivorum                       : AQASLAAGALPIGL------AHHARLR-RGIAEGEVVGWDDVDFAAGSALQTAIAARREMER-RF------A--APRAEAGAA----------- : 447
Acidovorax_ebreus_TPSY                        : AATSRSLRGLPLGL------AHSARVV-RPVAKGQAISWDDVEMDAT----THAYRVRREMEA-MF-----PA--------------------- : 446
Acidovorax_sp._JS42                           : AATSRQIKGLPLGL------AHSARVV-RPVAKGQAISWDDVAMDTT----THAYRVRREMEA-MF-----PA--------------------- : 446
Actinosynnema_mirum_DSM_43827                 : VMVAPEPGAAPYYL------LDGARLR-RPVAAGELVRLADIEGVAP----GPLDAHLFAQAQ-VA------A--AATEGAPA----------- : 440
Agrobacterium_sp._H13-3                       : ATRSLDIKAIPIGL------AHNVKLK-RAIKKDEIVSFDDVELVSD----LDVVKLRQNMEN-QF------R--PQKDAAA------------ : 452
Agrobacterium_tumefaciens_str._C58            : ATRSLDIKALPIGL------AHNVKLK-RPIKKDEIVSFDDVELVSD----LDVVKLRQDMEH-QF------R--PQKDAAA------------ : 489
Anaeromyxobacter_sp._Fw109-5                  : AADARRAGAIPLGL------AHGVRLR-APVRAGDPVRWTDVEVDEG----AEAVRLRRAALEA-TL------D--PPLHPRPASG--------- : 455
Arthrobacter_sp._FB24                         : AKHSVATGALPVAL------AHHVELR-NDVAKGGIVGWDDVIMDDS----LSQALEVRRETEA-LV------A--EFALNA------------ : 450
Azorhizobium_caulinodans_ORS_571              : ASKSLALRGLPIGL------AHNVKLK-RPVARDQIVSIDDVELEAD----LDVVALRRQMED-AA------R--PKAARSAA----------- : 453
Bordetella_avium_197N                         : ARISTDDAYLPIGL------AEGAVLK-HAVRQSQPIRTLDVSYDEE----TLAARLRREMEQ-AF------A---------------------- : 445
Bordetella_bronchiseptica_RB50                : ARDSVADGCLPIGL------AHNVRLK-HPVRQSQAIRWSDVEYDER----SPAVQFRRLMEQ-TF------A---------------------- : 445
Bordetella_parapertussis_12822                : ARDSVADGCLPIGL------AHNVRLK-HPVRQSQAIRWSDVEYDER----SPAVQFRRLMEQ-TF------A---------------------- : 445
Bordetella_petrii_DSM_12804                   : ARDSVKDGYLPIGL------AHKVKLK-NPVRQSQAIRWHDVDFDEQ----TPAVQFRREMEQ-SF------A---------------------- : 445
Bradyrhizobium_japonicum_USDA_110             : AASLKAGALPIGL------AHRVKLK-HDVAHGTVVRWDDVEFDAG----NETIRTRKAMEA-AF------A--KEG--------------- : 440
Bradyrhizobium_sp._BTAi1                      : AATSLQLGALPIGL------AHRVKLT-RDVAHGEVVRWSDVAIDTA----DETVSVRKQMEA-VF-----AR--------------------- : 434
Bradyrhizobium_sp._ORS278                     : ASASLQLGALPIGL------AHRVKLV-NDVAHGHVVRWSDVAADAA----DETVKYVRKQMET-AF------A--R------------------ : 440
Candidatus_Pelagibacter_ubique_HTCC1062       : SKKSKDEKILPIGL------TDGAIVK-KDINKDQSIRLDDVELDLP----EDVVKARQYQYD-LI------------------------------ : 430
Cupriavidus_necator_N-1                       : AKESLRVGSLPLGL------AHHLKLR-RDVAKDQSLTWEDVVIDAE----QPAYRVRKEMEA-LF-----QK--------------------- : 446
Cupriavidus_taiwanensis                       : ARESLRVGSLPLGL------AHHLKLR-RDVRKDQSLTWDDVVIDAE----LPAYRVRKEMEA-LF-----QK--------------------- : 446
Methylibium_petroleiphilum_PM1                : AATSLAGGYLPIGL------AHKVRLK-RDIADGRPVGWNDVEFDAR----SEAVQFRREMEA-AF------R---------------------- : 445
Methylobacterium_nodulans_ORS_2060            : ASKSLALGALPIGL------AHNVKLK-RPVGKDEVVRFDDVELEND----LDVVALRRQMED-GL------R--RATQKAA------------ : 452
Methylobacterium_radiotolerans_JCM_2831       : AAESLAQDALPIGL------AHNMVLK-TDVPAGRAVRWSDVAYDAG----QEAIRVRREMED-LF------R--GATGRPAG----------- : 453
Methylobacterium_sp._4-46                     : AAKSLALGALPIGL------AHNVALK-RPVGKDEVVRFDDVELVND----LDVVALRRQMED-AA------R--QAPPKAA------------ : 452
Mycobacterium_smegmatis_str._MC2_155          : AEISLDRNALPLGL------AHGATLV-RDIAKDEISWDDVRVDES----QFAVKLRRQLES-EF------R--SEHIAATA------------ : 454
Nocardiopsis_dassonvillei_subsp._dassonv      : AADAPDDVA-PLYL------AAHAALG-RDVAAGALVRLDDADASA----PLLDAWRHARTH-------------L--------------------- : 456
Paracoccus_denitrificans_PD1222               : AGPLSDESPAPFYL------ASNQTLR-RPVAAGSYLRMGDLEHRSN----TELLTERQRQDA-IF------A--KAKGMQG------------ : 467
Polaromonas_sp._JS666                         : AETSLQMGGLPIGL------AHQVKVV-RAVKKGQSLSWADVAMDTS----TPAYKVRHEMET-MF------S--PAIKKAA------------ : 452
Polymorphum_gilvum_SL003B-26A1                : ASVSLARGHLPIGL------AHHVTLT-RDVAEGEMLTWDDVVIDET----DDAYRFRREMEQ-AF------A---------------------- : 445
Polynucleobacter_necessarius_subsp._asym      : AADSLKLGALPIGL------AHNMALK-RDIPAGKPVCWSDVDYDAT----KQAIAFRREMET-VF-----GK--------------------- : 446
Pusillimonas_sp._T7-7                         : AQKSVQLGGLPIGL------AHGIKLL-RPVAKGQAISWSDVAIDTG----TPAYRVRKEMES-IF------V--PQAQ--------------- : 449
Rhodopseudomonas_palustris_BisB5              : ASDSLTAGALPIGL------AHRVRLT-SDVGHGGVVRWCDVEIDKS----DPTVATRAMEQ-AF----SGR--------------------- : 435
Rhodospirillum_rubrum_ATCC_11170              : ADPQSTALA-PFYL------AANRTLV-TDVAAGTLITPAMLDLEGS----ALYDLWRSPV---------------------------------- : 455
Spirochaeta_smaragdinae_DSM_11293             : AQGISAHTQLPYYL------ADGARLK-EDIPAGTLINADMLERPDD----AILWKLREEQDT-IF------A--L------------------- : 462
Spirochaeta_sp._Buddy                         : AQEAPIDAA-PYYL------IAGKKLT-KDVQKGEVITLDMLDLAGS----ELERMYLREGKNI-------------------------------- : 457
Streptomyces_flavogriseus_ATCC_33331          : DEDAPADTA-PLYL------AAHATLG-RDVPAGALITLGDLAPDDA----DLLHAWTSGRGS-RA------T--GTTDAPHASR--------- : 470
Streptomyces_sp._SirexAA-E                    : DEDTPSDTA-PLYL------AAHATLG-RDVRAGDLITLGDLADADT----ELLHAWASGRAS-RT------T---HSPVPHASR--------- : 469
Variovorax_paradoxus_EPS                      : AERSLRLGGLPLGL------AHNVKLV-RPVKKGQSLSWADVAIDTS----TAAYKLRSEMES-MF------S--AAVQTRAA----------- : 453
Variovorax_paradoxus_S110                     : AERSLGLGGLPLGL------AHSVKLV-RPVKKGQSLSWADVAMDTS----TSAYRLRSEMEA-MF------A--PAAQAKAA----------- : 453
Xanthobacter_autotrophicus_Py2                : ASLSLAHGGLPIGL------AHNVVLK-NPVAGGQPVRWADVTFDET----QQAVKVRREMES-LF------R--AADSAGQKAPAAA------- : 458
```
