# Supplementary figures and images for: Evolutionary diversification and characterization of the eubacterial gene family encoding DXR type II, an alternative isoprenoid biosynthetic enzyme
Source: BMC Evol Biol. 2013 Sep 3;13:180. doi: 10.1186/1471-2148-13-180 (PMC3847144; doi:10.1186/1471-2148-13-180)

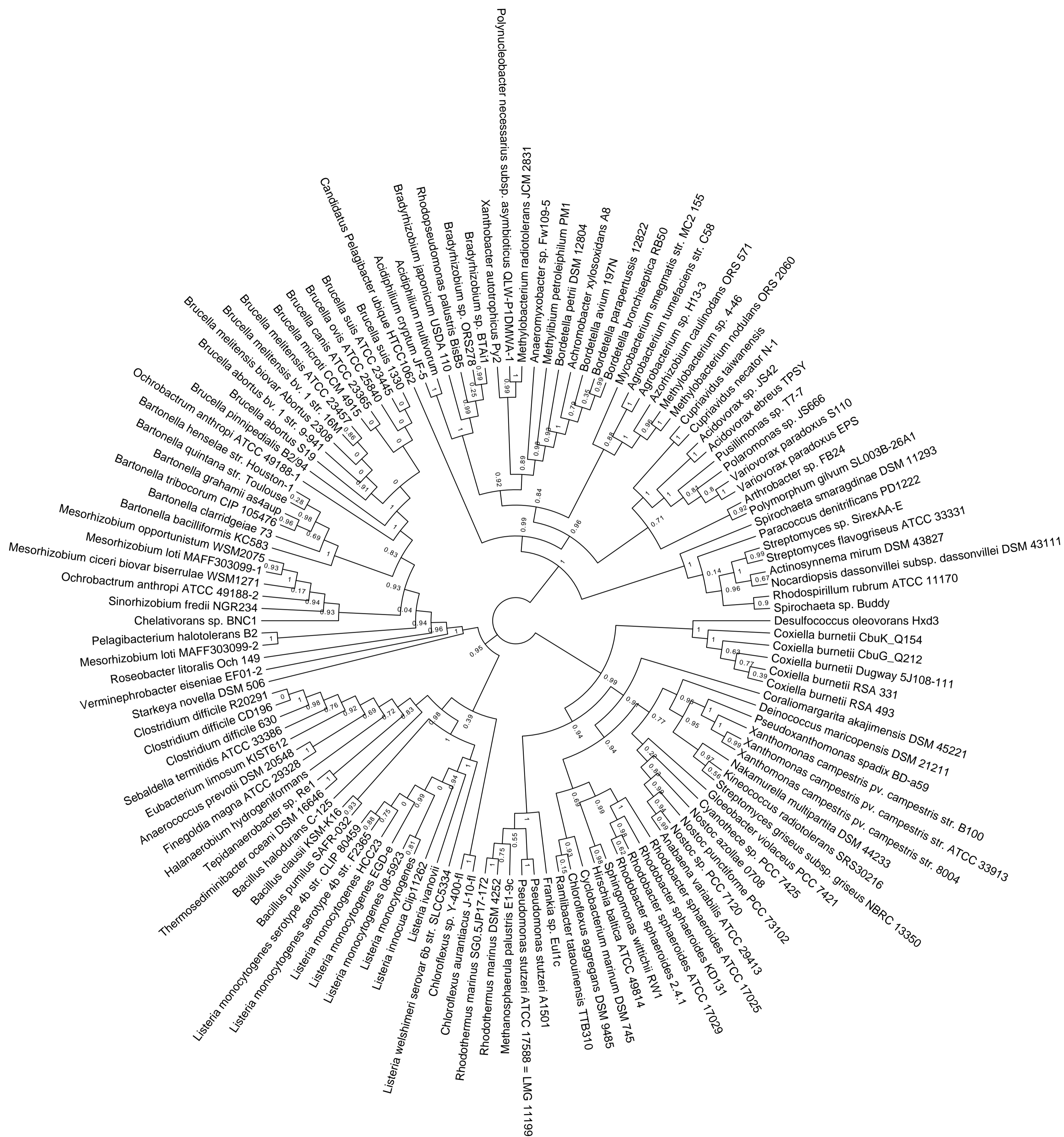

Supplement: Additional file 2 — ML phylogeny of DXR-II and DLO related sequences. ML cladogram depicting the evolutionary relationships among 53 DXR-II and 77 related protein sequences. Statistical support for clades (ML aLRT support values) is indicated next to nodes. [file 1471-2148-13-180-S2.pdf]

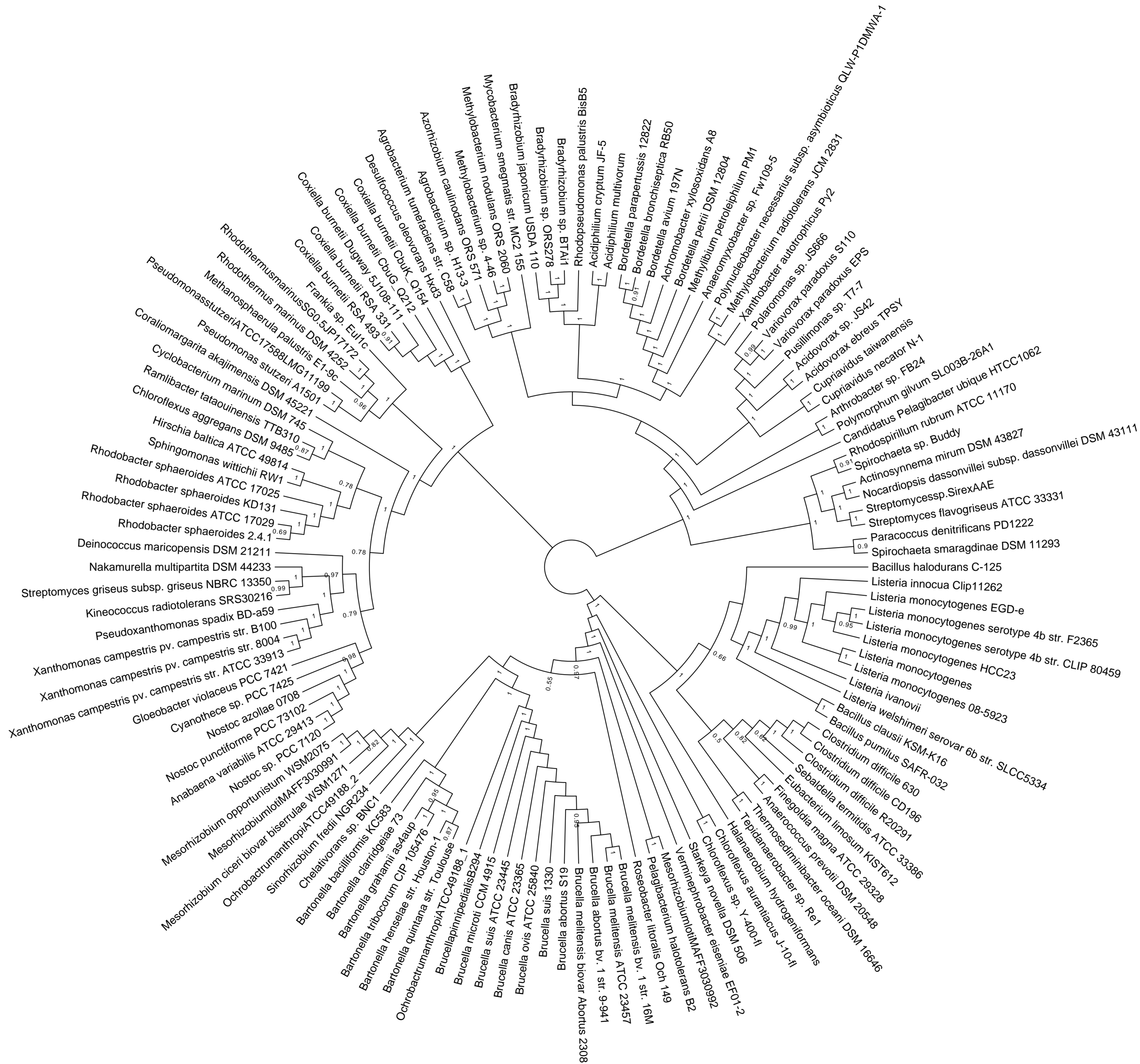

Supplement: Additional file 3 — Bayesian phylogeny of DXR-II and DLO related sequences. Bayesian cladogram depicting the evolutionary relationships among 53 DXR-II and 77 related protein sequences. Statistical support for clades (posterior probabilities) is indicated next to nodes. [file 1471-2148-13-180-S3.pdf]

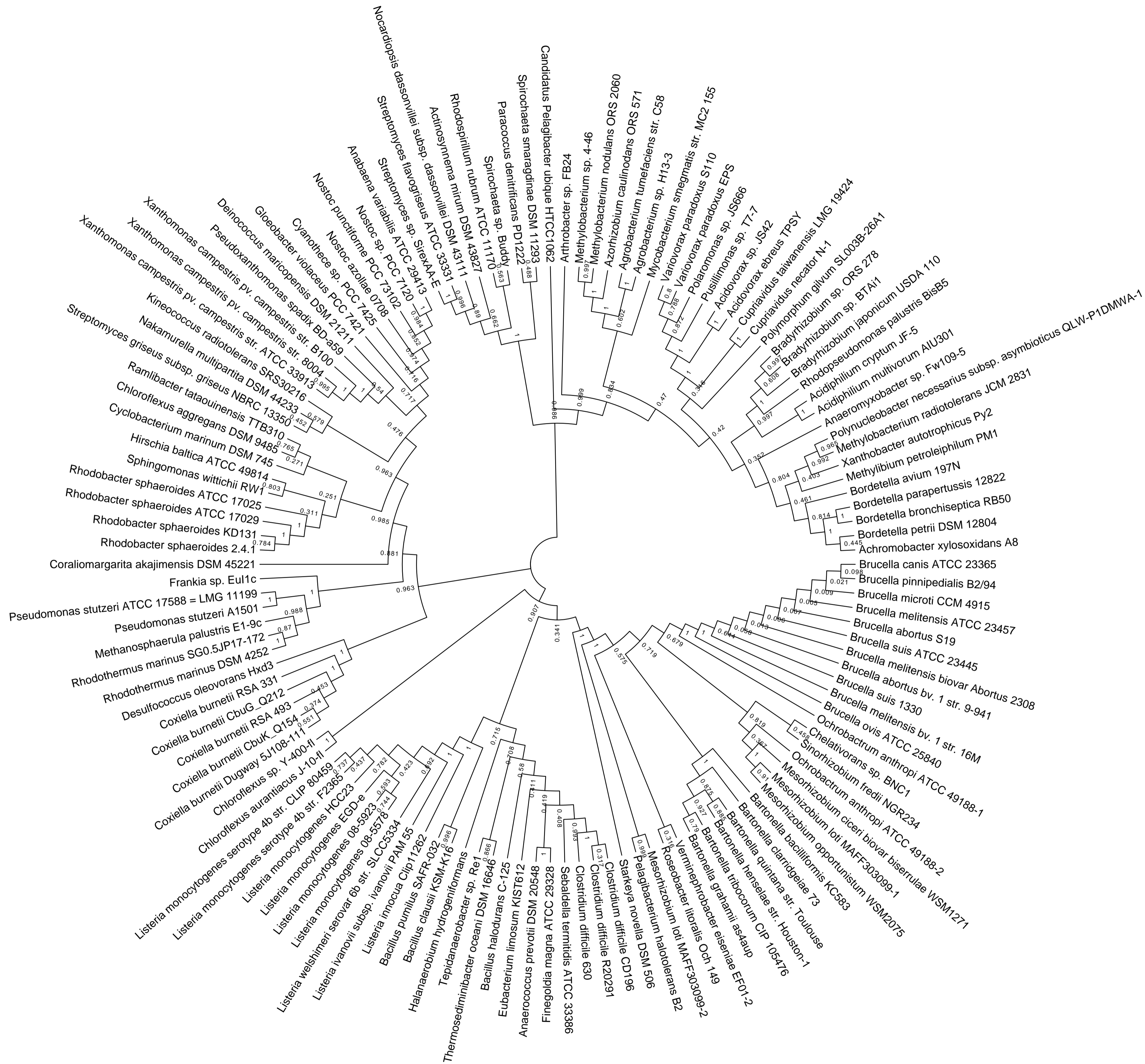

Supplement: Additional file 4 — Neighbor Joining phylogeny of DXR-II and DLO related sequences. Neighbor Joining cladogram depicting the evolutionary relationships among 53 DXR-II and 77 related protein sequences. Statistical support for clades (bootstrap values) is indicated next to nodes. [file 1471-2148-13-180-S4.pdf]

+ MVA

- MVA

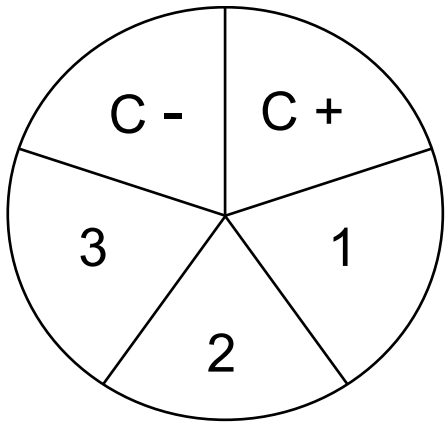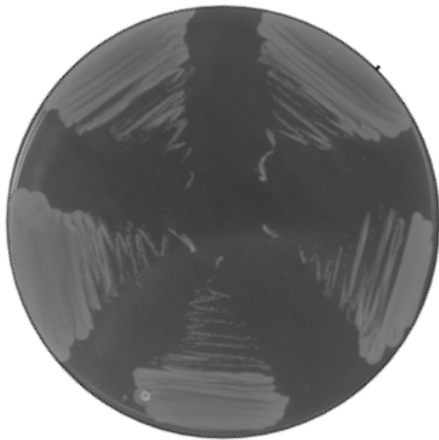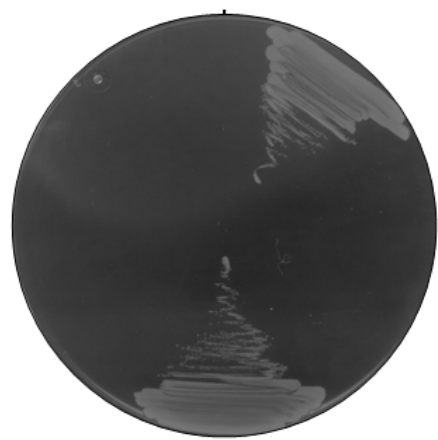

Supplement: Additional file 5 — Complementation of DXR-deficient E. coli cells with putative DXR-II sequences from Chloroflexus auranticus J-10-fl. The putative DXR-II sequences were PCR-amplified from genomic DNA and cloned into pJET1.2. The corresponding constructs and positive and negative controls (C-, empty vector; C+, DXR-II (YP_418479.1) from B. melitensis biovar abortus 2308) were used to transform EcAB4-10 cells [23]. Ability of the cloned gene to rescue growth of this DXR-deficient mutant strain was ascertained by monitoring growth on plates either supplemented (+) or not (−) with 1 mM MVA as indicated. 1) YP_001634831.1, 2) YP_001634944.1, and 3) YP_001636771.1. [file 1471-2148-13-180-S5.pdf]

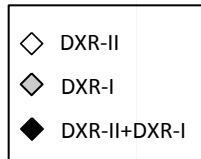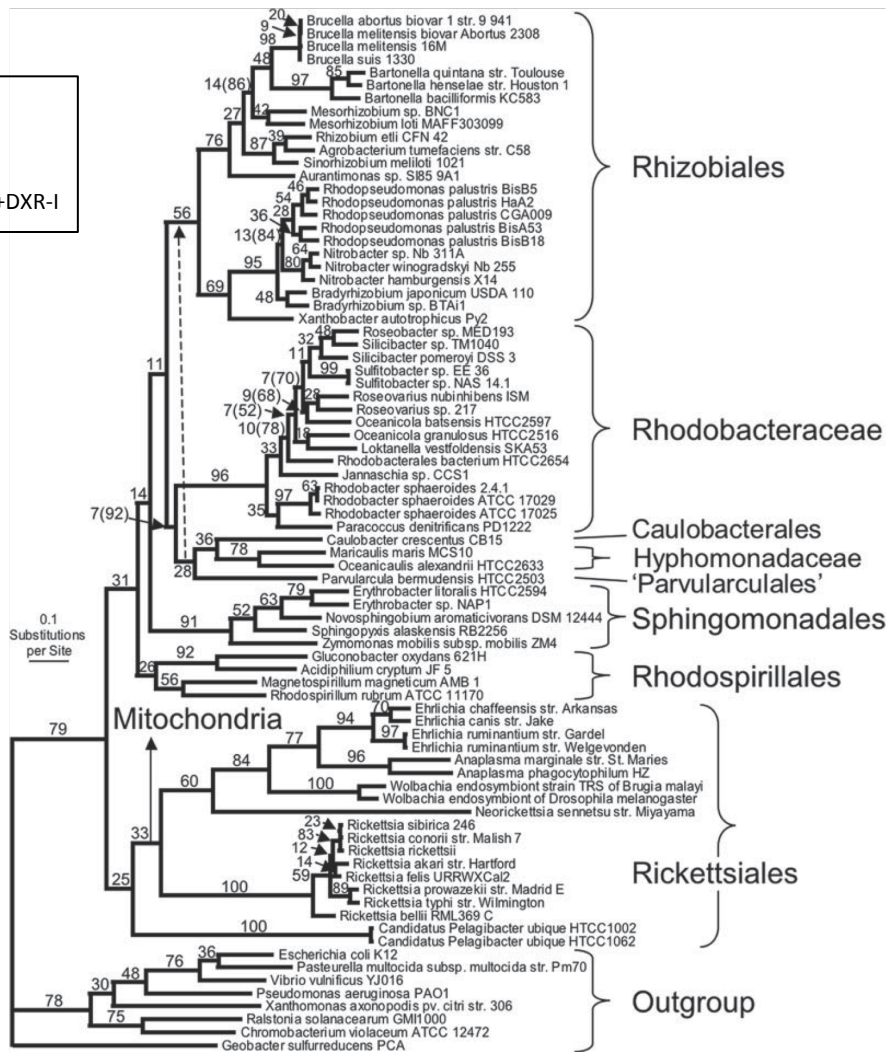

Supplement: Additional file 7 — Distribution of DXR-I and DXR-II in Alphaproteobacteria. The occurrence of DXR-I and DXR-II is represented for alphaproteobacterial strains with full sequenced genomes in a phylogenetic context, according to the robust species tree reported in [30]. [file 1471-2148-13-180-S7.pdf]

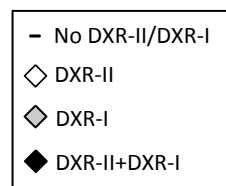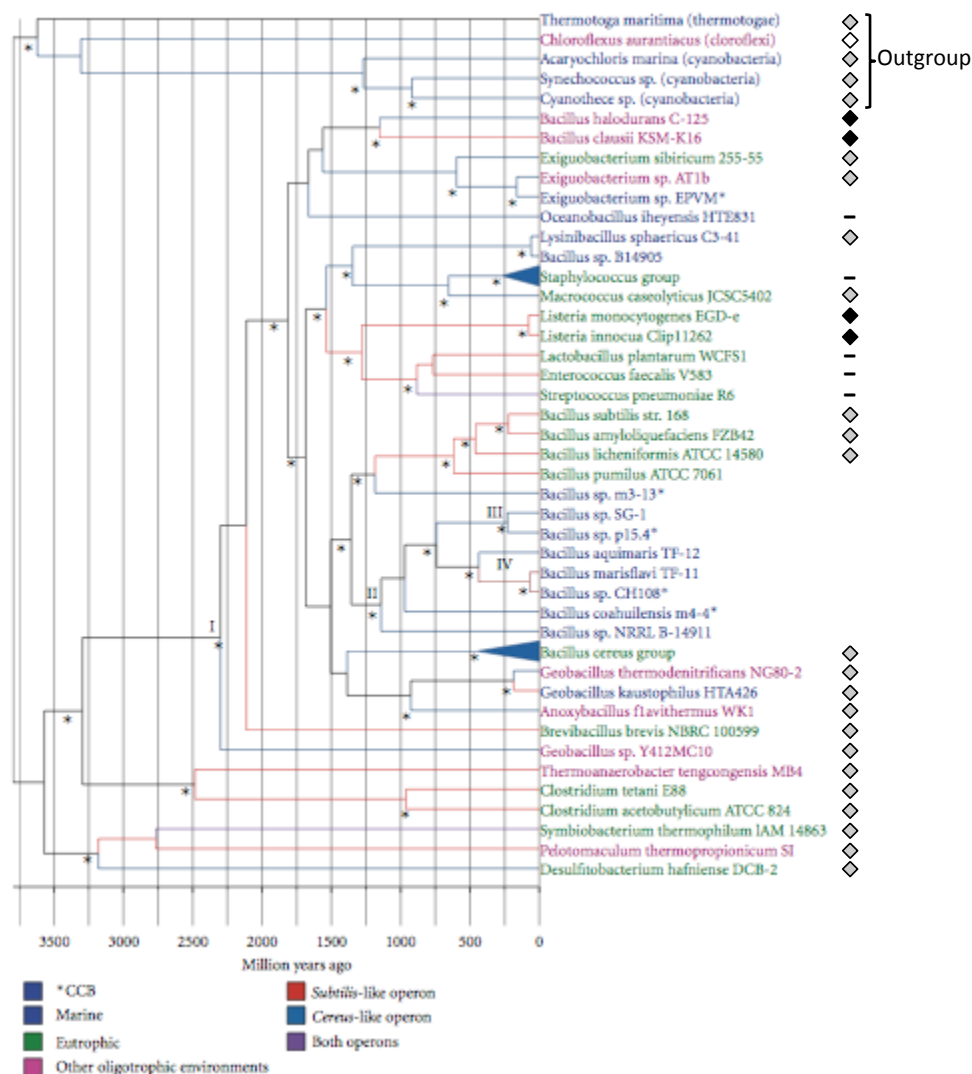

Supplement: Additional file 8 — Distribution of DXR-I and DXR-II in Firmicutes. The occurrence of DXR-I and DXR-II is represented for strains with full sequenced genomes in a phylogenetic context, according to the robust species tree for Firmicutes reported in [32]. [file 1471-2148-13-180-S8.pdf]
